# Brain Endothelial Gap Junction Coupling Enables Rapid Vasodilation Propagation During Neurovascular Coupling

**DOI:** 10.1016/j.cell.2025.06.030

**Published:** 2025-07-16

**Authors:** Trevor Krolak, Luke Kaplan, Kathleen Navas, Lujing Chen, Austin Birmingham, Daniel Ryvkin, Victoria Isza, Megan Powell, Zhuhao Wu, Benjamin E. Deverman, Chenghua Gu

**Affiliations:** 1 Department of Neurobiology, Howard Hughes Medical Institute, Harvard Medical School, Boston, MA, USA.; 2 Stanley Center for Psychiatric Research, Broad Institute of MIT and Harvard, Cambridge, MA, USA.; 3 Weill Cornell Medicine Helen & Robert Appel Alzheimer’s Disease Research Institute, New York, NY, USA.; 4 Lead contact.

## Abstract

To meet the brain’s moment-to-moment energy demand, neural activation rapidly increases local blood flow. This process, known as neurovascular coupling, involves rapid, coordinated vasodilation of the brain’s arterial network. Here, we demonstrate that endothelial gap junction coupling enables long-range propagation of vasodilation signals through the vasculature during neurovascular coupling. The molecular composition of these gap junctions is zonated along the arterio-venous axis, with arteries being the most strongly coupled segment. Using optogenetics and visual stimuli in awake mice, we found that acute, arterial endothelial cell type-specific deletion of Cx37 and Cx40 abolishes arterial gap junction coupling and results in impaired vasodilation. Specifically, we demonstrated that arterial endothelial gap junction coupling determines both the speed and the spatial extent of vasodilation propagation elicited by neural activity. These findings indicate that endothelial gap junctions serve as a signaling highway for neurovascular coupling, enabling flexible and efficient distribution of limited energetic resources.

## In Brief

Vasodilatory signals are rapidly communicated across long distances by endothelial-endothelial gap junctions, enabling coordinated dilation of the arterial network during neurovascular coupling.

## Graphical Abstract

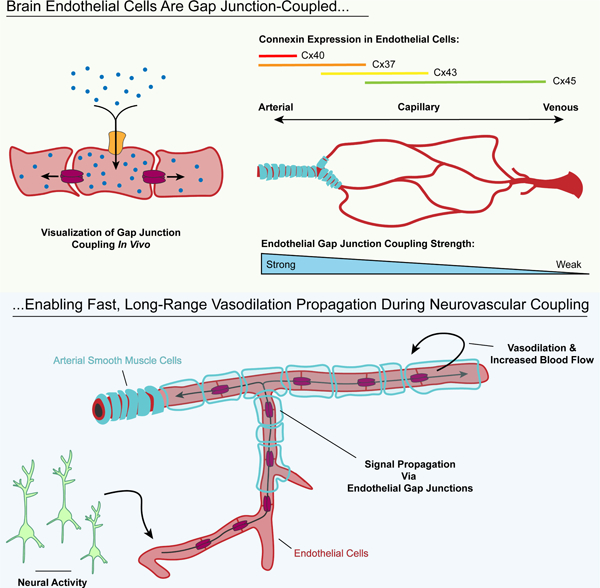

## Introduction

A highly regulated energy supply is of fundamental importance for brain health and performance. The neural activity underlying cognition is energetically demanding: the human brain consumes 20% of the body’s cardiac output at rest, despite constituting only 2% of its mass ^1,2^. Moreover, this budget is thought to be fixed – regardless of attentional state or task performance, total cerebral perfusion remains roughly constant ^3,4^. To meet moment-to-moment changes in regional energy demand, the brain has evolved a finely tuned mechanism whereby neural activation rapidly increases local blood flow, a process known as neurovascular coupling. This functional coupling between neural activity and local blood flow is not only important for the daily function of the brain; it is also the basis for functional brain imaging in humans ^5^. Impaired neurovascular coupling is increasingly associated with aging and neurodegenerative disease ^6,7^. Despite the importance of neurovascular coupling, the underlying cellular and molecular mechanisms are not clear.

During neurovascular coupling, neural signals ultimately lead to the relaxation of smooth muscle cells (SMCs) that enwrap brain arteries and arterioles, producing vasodilation and subsequent increases in blood supply to active regions ^8^. However, because vascular resistance is broadly distributed, dilation of any given vessel segment exerts limited control over downstream perfusion ^9^. As a result, millimeter-scale stretches of the brain’s arterial network must be concurrently mobilized to produce robust changes in local blood flow ^10^. Despite the large distances involved, activity-evoked dilation occurs within hundreds of milliseconds with remarkable spatial precision, selectively engaging elements of the arterial network upstream of the active site ^11,12^. This fast and flexible recruitment of the arterial network is a key feature of neurovascular coupling and ensures that the brain spends its limited energy budget efficiently and adequately. However, partially hampered by a lack of experimental tools and paradigms to visualize and manipulate this process in awake animals, the mechanistic basis of this rapid arterial recruitment remains largely unknown.

Here, we demonstrate that endothelial gap junction coupling enables rapid, long-range propagation of vasodilation through arteries during neurovascular coupling. By developing a non-invasive *in vivo* tracing method, we found that brain endothelial cells are functionally coupled by gap junctions – clusters of intercellular channels made of connexin proteins that enable direct diffusion of ions and small molecules between adjacent cells ^13^. Moreover, we observed that four connexin isoforms are differentially expressed by endothelial cells across the arterio-venous axis. To elucidate the role of endothelial gap junctions in neurovascular coupling, we developed visual and optogenetic stimulation paradigms to evoke spatially defined patterns of neural activity in awake mice while imaging the resulting vascular responses. We found that acute, cell type-specific deletion of Cx37 and Cx40 abolishes arterial gap junction coupling and results in impaired vasodilation. Specifically, we demonstrated that arterial endothelial gap junction coupling determines both the speed and the spatial extent of vasodilation propagation elicited by neural activity. These findings indicate that endothelial gap junctions along the cerebrovasculature serve as a signaling highway for neurovascular coupling, enabling flexible and efficient distribution of limited energetic resources.

## Results

### Non-invasive visualization of gap junction coupling *in vivo*

Previous studies have shown that stimulation of brain capillaries can drive dilation of upstream arterioles ^14–17^ and that arterial endothelial cells are indispensable for neurovascular coupling ^18,19^. Anatomically, brain endothelial cells are optimally positioned to serve as a highway bridging fast communication between neurons and arterial smooth muscle cells. Gap junctions, known for their role in fast electrical communication among neurons and muscles, might be the molecular substrate connecting neighboring endothelial cells to enable rapid signaling over long distances. However, due to technical limitations, the extent of functional cell-cell coupling throughout the brain endothelium is largely unexplored. Conventional methods used to measure gap junction coupling, such as intracellular injection of small-molecule tracers, are challenging to implement in these extremely thin, physically inaccessible cells.

To overcome these obstacles, we sought to develop a non-invasive gap junction tracing strategy that we could deploy in the cerebrovasculature *in vivo*. Previous work in cell culture has shown that serotonin, a small gap junction-permeable molecule (~175 Da), is rapidly internalized by cells exogenously expressing the serotonin transporter (SERT) and readily diffuses to neighboring SERT-negative cells via gap junctions ^20^. Because serotonin is aldehyde-fixable and can be detected with specific antibodies, immunostaining can be used to visualize this intercellular transmission.

**Figure 1.**
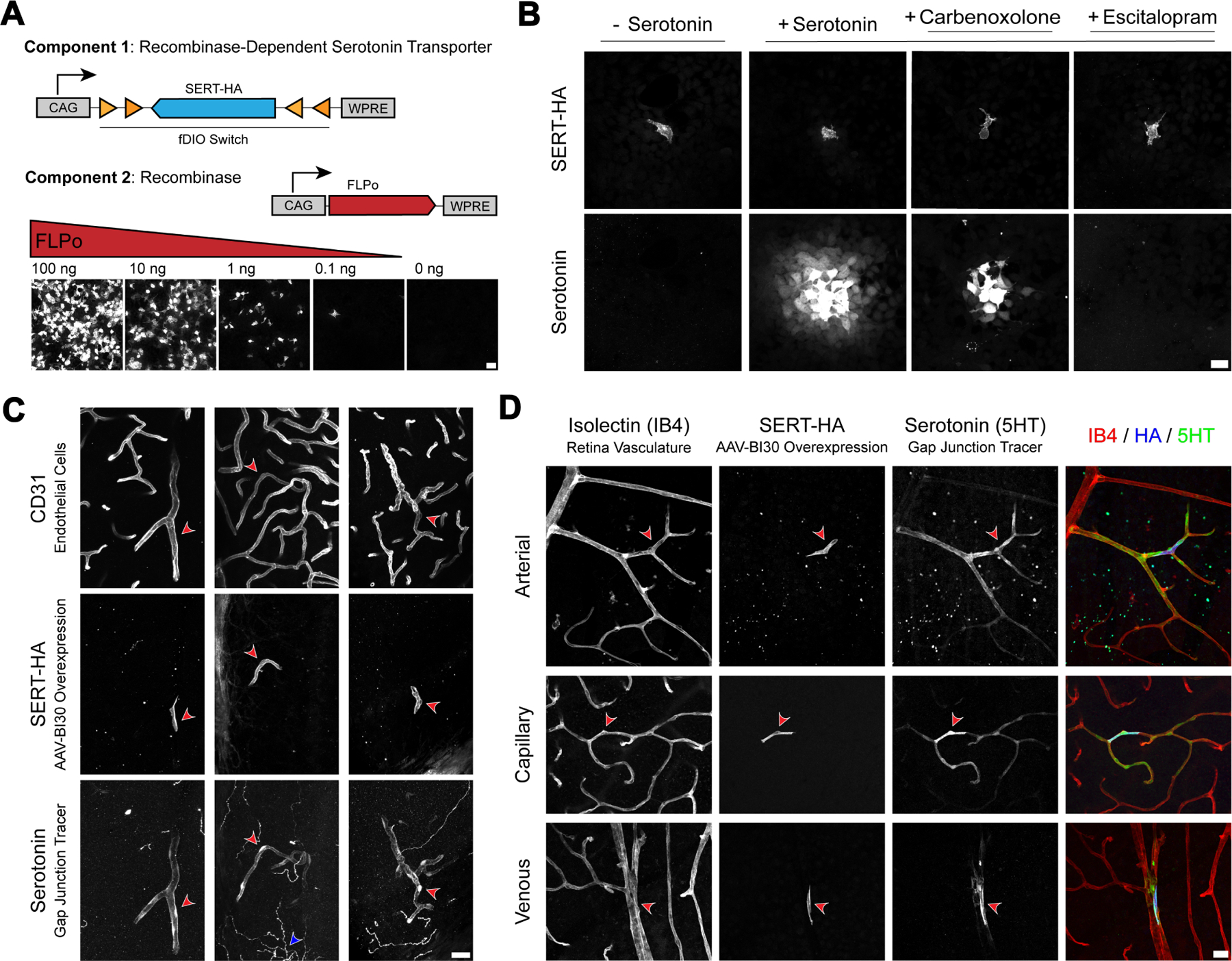
Non-invasive gap junction tracing reveals robust endothelial-endothelial coupling in the CNS vasculature. **(A)** Strategy for sparse-but-strong delivery of the serotonin transporter (SERT) using a two-component recombinase-based expression system. As the dose of FLPo delivered in conjunction with a constant quantity of FLP-dependent SERT is progressively titrated, the frequency of SERT-expressing cells decreases without compromising expression strength. Images in dose curve show transiently transfected HEK293 cells immunostained for SERT-HA. **(B)** Demonstration of non-invasive, SERT-based gap junction tracing *in vitro*. Cells were co-transfected with a high dose of FLP-dependent SERT and a low dose of FLPo recombinase to achieve sparse expression. A brief extracellular pulse of serotonin was delivered immediately prior to fixation, and anti-serotonin immunolabeling was used to visualize tracer spread. (**C, D**) To implement this tracing strategy *in vivo*, a cocktail of AAV-BI30 capsids packaged with FLPo or FLP-dependent SERT was intravenously administered to adult mice. After allowing >6 weeks for overexpression, animals were perfused with a serotonin-containing solution to achieve cell loading. Immunostaining reveals gradients of serotonin emanating from SERT-positive endothelial cells (red arrowheads) in brain (**C**) and retina (**D**). Note extravascular serotonergic fibers in brain (blue arrowhead). Scale bars are as follows: 50µm in (**A**); 25µm in (**B**), (**C**), and (**D**). Images are representative of *n* = 3 experimental replicates for (**A**) and (**B**) and endothelial cell-fills observed across *n* = 4 or 5 mice for (**C**) and (**D**), respectively.

Inspired by this *in vitro* assay, we developed a two-component strategy ^21^ wherein SERT expression was contingent on the co-expression of a recombinase to achieve strong-but-sparse overexpression of the transporter needed for *in vivo* tracing. By titrating the amount of recombinase delivered to cells, we achieved strong expression in individual cells with controllable sparseness (Figure 1A). As a proof-of-concept, we delivered a transient pulse of serotonin to the media of HEK293T cells co-transfected with these constructs and observed robust serotonin gradients emanating outwards from SERT^+^ cells to their SERT^−^ neighbors. This intercellular transfer was significantly blunted by carbenoxolone, a gap junction inhibitor, or eliminated altogether by escitalopram, a selective serotonin reuptake inhibitor (Figure 1B).

### CNS endothelial cells are functionally coupled

To examine endothelial cell coupling in the CNS vasculature, we next deployed this two-component system *in vivo*. A cocktail of CNS endothelial cell-specific viruses (AAV-BI30) ^22^ carrying either FLPo recombinase or FLP-dependent SERT was intravenously delivered to adult mice, producing high expression of SERT in a sparse subset of endothelial cells. Next, we transcardially perfused mice with a serotonin-containing solution, an approach that allowed us to simultaneously capture numerous cell-loading events within the intact vasculature of a single animal. Using this strategy, we observed robust serotonin uptake into SERT-expressing endothelial cells in the cerebellum, a brain region with comparatively few serotonergic inputs, facilitating visualization of our tracer (Figure 1C). Strikingly, serotonin readily passed from these SERT-expressing cells into neighboring endothelial cells and diffused hundreds of microns along the vasculature, demonstrating the existence of extensive endothelial-endothelial gap junction coupling. Importantly, serotonin uptake was restricted to cells exogenously overexpressing the transporter, a result consistent with undetectable levels of SERT (*Slc6a4*) expression in CNS endothelial cells ^23^.

We observed similar coupling events in the retina, where planar organization of the vasculature and a complete absence of serotonergic innervation allowed us to precisely resolve segment-specific features. Here, an arterio-venous gradient of endothelial gap junction connectivity became apparent. Arterial and capillary endothelial cells exhibited robust serotonin transfer to their neighbors, revealing extensive gap junction-coupled networks. By contrast, coupling was comparatively suppressed in venous segments, with serotonin largely restricted to SERT-expressing cells (Figure 1D, Figure S1A–C). Interestingly, variation in the strength of gap junction coupling exhibited by these endothelial subtypes paralleled their functional relevance to neurovascular coupling; while arterial ^18,19^ and capillary ^14–16^ endothelial cells have been shown to play active roles in neurovascular coupling, venous endothelial cells do not appear to participate in the process. By developing a non-invasive gap junction tracing method, we established the existence of endothelial-endothelial gap junction coupling in the CNS. Intriguingly, the coupling strength shows an arterio-venous gradient.

### Connexin isoforms are differentially expressed in CNS endothelial cells along the arterio-venous axis

We next sought to identify the connexins expressed by CNS endothelial cells. Gap junctions are formed by connexins, a large family of ~20 integral membrane proteins. Among them, Cx37 (*Gja4*), Cx40 (*Gja5*), Cx43 (*Gja1*), and Cx45 (*Gjc1*) are generally thought to be expressed in the vasculature ^13^. To identify the specific connexins that form gap junctions in CNS endothelial cells, we examined endogenous locus knock-in reporter mice targeting each of these isoforms: Cx40 (ref. ^24^), Cx43 (ref. ^25^), and Cx45 (ref. ^26^) as well as a Cx37 reporter we generated for this study (Figure S2).

**Figure 2.**
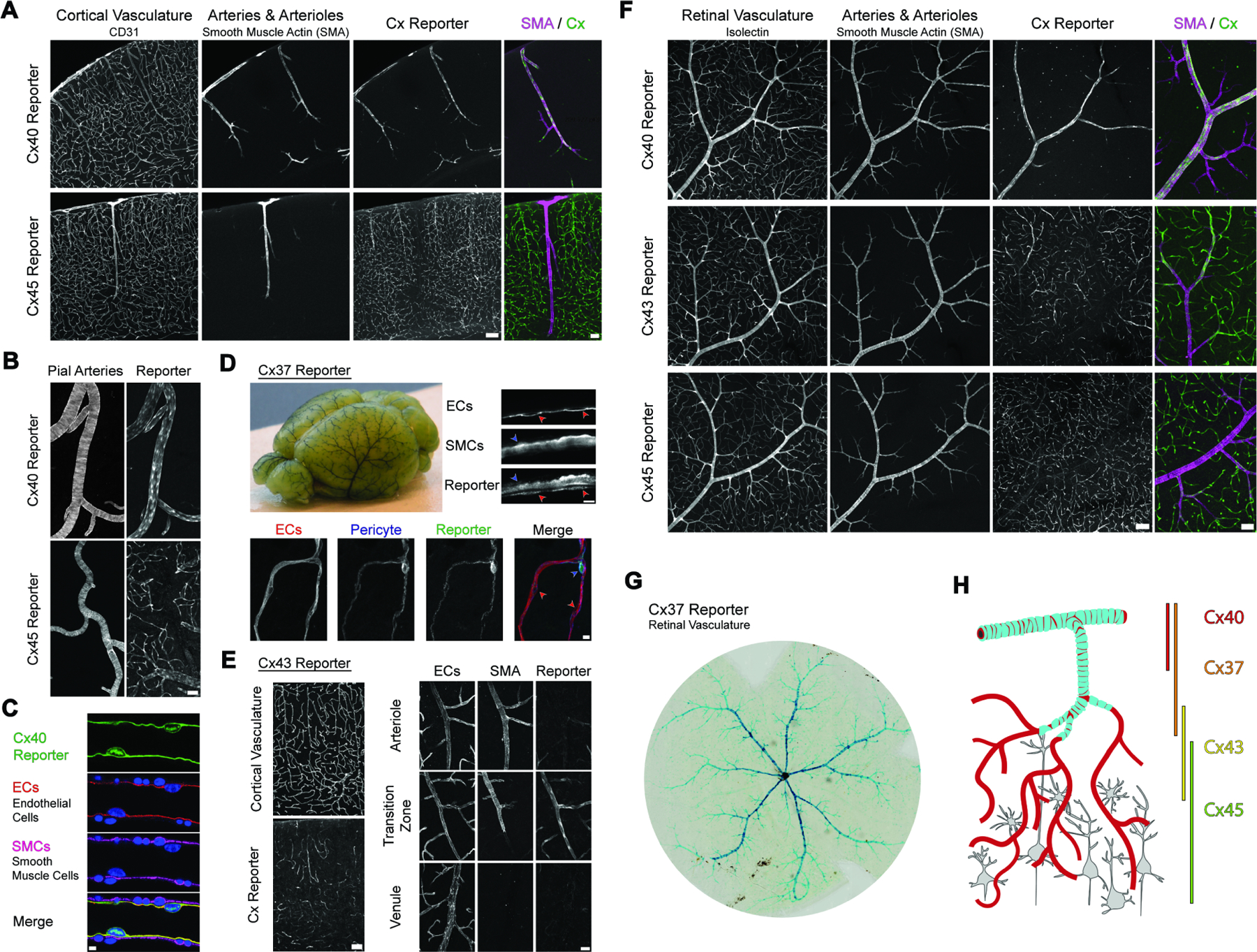
Expression of endothelial connexin isoforms is organized along the arterio-venous axis in brain and retina. (**A**) Tissue clearing and immunolabeling of cortical microvasculature reveals complementary expression of Cx40 (*Gja5*^*GFP/+*^) and Cx45 (*Tie2:Cre Gjc1*^*fl-KI-GFP/+*^). (**B**) Images of pia vasculature from the same reporter lines. (**C**) Cross section of an individual arteriole from a Cx40 reporter animal, showing exclusive co-localization of GFP transgene with endothelium. (**D**) Top left: whole-brain expression of enzymatic Cx37 (*Gja4*^*LacZ/+*^) reporter; note robust LacZ signal in the middle cerebral artery and its branches emanating across the pial surface. Top right: immunodetection of ß-Galactosidase transgene in thin tissue sections, revealing expression of Cx37 in both the endothelium (red arrowheads) and smooth muscle (blue arrowhead) layers of the arterial wall. Bottom: with progression into capillary microvasculature, ß-Galactosidase immunostaining becomes restricted to the mural cell layer (pericyte; blue arrowhead) and is no longer detectable in the endothelium (red arrowheads). (**E**) Left: low-magnification image of Cx43 (*Tie2:Cre Gja1*^*fl-KI-CFP/+*^) reporter expression in cortical microvasculature. Right: high-magnification images reveal that Cx43 expression peaks at arterio-capillary transitions and is absent in larger arterioles and venules. (**F, G**) The same patterns of expression are observed in the retina using both fluorescent (**F**) and enzymatic (**G**) connexin reporters. (**H**) Schematic illustrating stepwise zonation of connexin expression in CNS endothelial cells. Scale bars are as follows: 100µm second column from right, 50µm rightmost column in (**A**); 50µm in (**B**); 5µm in (**C**); 5µm both panels in (**D**); 75µm leftmost panel, 25µm rightmost column in (**e**); 100µm second column from right, 50µm rightmost column in (**f**). Images shown are representative of *n* = 3 animals per genotype. Pial Arteries (**B**) and SMCs (smooth muscle cells) (**D**) identified with anti-SMA (smooth muscle actin); ECs (endothelial cells) (**D, E**) and Cortical Vasculature (**E**) identified with anti-CD31; Pericyte (**D**) identified with anti-PDGFRß.

Surprisingly, we found that the expression of connexin isoforms in brain endothelial cells is markedly zonated along the arterio-venous axis. Signal from a Cx40 reporter line (*Gja5* ^*GFP/+*^) was exclusively expressed in arterial endothelial cells and was even excluded from small, SMA^+^ terminal arterioles (Figure 2A–C). Conversely, an endothelial-specific Cx45 reporter (*Tie2:Cre Gjc1* ^*flox-KI-GFP*^) exhibited a complementary pattern of expression, with strong signal in veins, venules, and capillaries - but minimal expression in the arterial network (Figure 2A, B). A Cx37 reporter (*Gja4* ^*LacZ/+*^) displayed a pattern of arterial expression reminiscent of Cx40 but appeared to extend into smaller arterioles and was also detectable in vascular mural cells – pericytes and smooth muscle cells (Figure 2D). Finally, signal from an endothelial-specific Cx43 reporter (*Tie2:Cre Gja1* ^*flox-KI-CFP*^) abruptly emerged at arteriole-to-capillary transitions and extended through the capillary bed before again diminishing in venules (Figure 2E). This tiling of connexin isoforms along the arterio-venous axis was conserved in the retina (Figure 2F, G), where planar organization of the vasculature readily revealed a stepwise cascade of expression, with arterial Cx40 followed by Cx37, Cx43, and finally Cx45 (Figure 2H). This expression pattern was further supported by single-cell sequencing data from recent efforts to transcriptionally profile the CNS vasculature ^23,27^ (Figure S3A, B).

This arterio-venous organization of connexin expression provocatively paralleled the gradation of cell-cell coupling strength revealed by our non-invasive tracing assay, indicating that shifting utilization of connexin isoforms might underlie distinctions in endothelial gap junction coupling. Moreover, this zonation of isoform expression was endothelial-specific rather than a generic property of the vasculature: we found that mural cells associated with CNS arteries, capillaries, and veins invariantly expressed Cx37 and Cx45 (Figure S3C–G). Thus, these results demonstrate that brain endothelial cells are provisioned with the molecular machinery required for long-range, gap junction-mediated communication – and that diversity in the genes utilized to produce such connectivity could give rise to segment-specific features.

### Acute, cell type-specific deletion of Cx37 and Cx40 abolishes gap junction coupling in arterial endothelial cells

**Figure 3.**
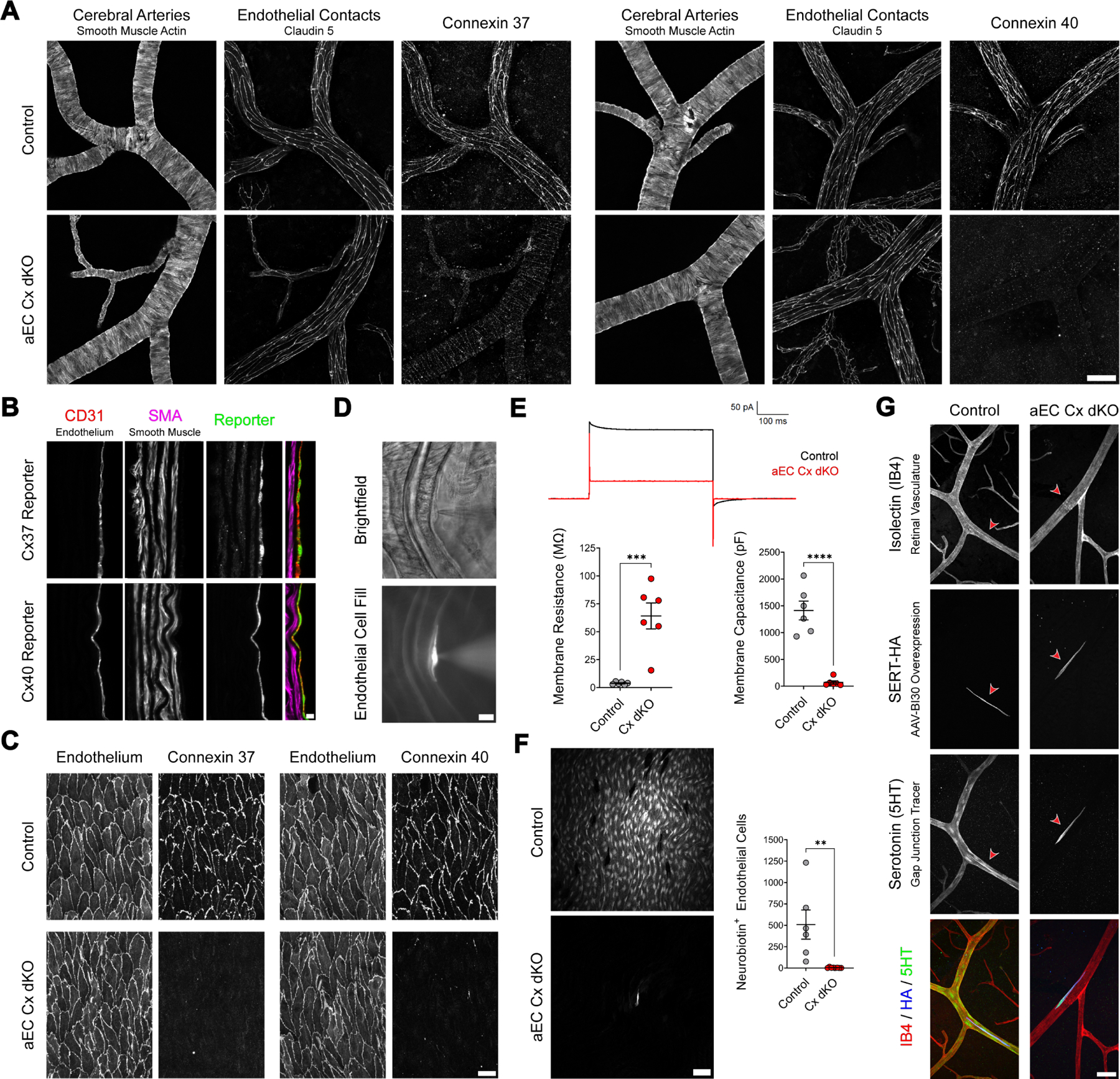
Acute ablation of Cx37 and Cx40 severely impairs arterial endothelial cell gap junction coupling. (**A**) Images of pial arteries demonstrate efficient removal of arterial endothelial cell gap junctions in tamoxifen-treated connexin conditional-knockout animals (*BMX:Cre*^*ERT2*^ *Gja4*^*fl/fl*^ *Gja5*^*fl/fl*^). Note that smooth muscle expression of Cx37, organized in punctate contacts running orthogonal to artery’s course, becomes readily evident following loss of the brighter endothelial signal. (**B**) Cryosections of the aortic wall taken from Cx37 (*Gja4*^*LacZ/+*^) and Cx40 (*Gja5*^*GFP/+*^) reporters show that arterial endothelial cell co-expression of these isoforms is conserved between brain and aorta. (**C**) *En face* preparations of the aorta demonstrate that both connexin proteins can be efficiently deleted from aortic endothelial cells using the pan-arterial *BMX:Cre*^*ERT2*^ driver. CD31 immunostaining identifies endothelium. (**D**) Using *en face* preparation, individual aortic endothelial cells can be accessed via whole-cell patch clamp. (**E**) Above: representative current traces obtained from control and connexin-knockout endothelial cells following application of a 10mV voltage step. Below: quantification of membrane resistance and membrane capacitance (*n* = 6 control / 6 connexin-knockout cells obtained from *n* = 2 animals per genotype). An average membrane resistance of 3.9 ± 0.5MΩ versus 64.1 ± 11.6MΩ was recorded in control cells and connexin-knockouts, respectively (p = 0.0004). Similarly, an average membrane capacitance of 1412 ± 175pF versus 71 ± 30pF was measured across the groups (p < 0.0001). (**F**) Left: representative images of gap junction tracing performed by incorporating neurobiotin into the patch pipette’s internal solution. Right: quantification of tracer spread (*n* = 6 control / 8 connexin-knockout cells obtained from *n* = 2 animals per genotype). An average of 509 ± 171 versus 4 ± 2 tracer-positive cells were counted in controls and connexin-knockouts, respectively (p = 0.0047). (**G**) SERT-based, non-invasive gap junction tracing in the retinal vasculature. The positions of SERT-HA expressing probe cells are demarcated with red arrowheads. For additional details, see Figure S5B. For quantification: mean ± s.e.m.; two-sample t-test (** p < 0.01, *** p < 0.001, **** p < 0.0001). Scale bars are as follows: 50µm in (**A**), (**F**), and (**G**); 5µm in (**B**); 25µm in (**C**); 10µm in (**D**). Unless otherwise specified, images shown are representative of *n* = 3 animals.

To elucidate the role of endothelial gap junctions in rapid vasodilation propagation through a large arterial network, we developed a genetic loss-of-function mouse line enabling acute ablation of arterial endothelial cell-cell coupling. Our expression data suggested that ablation of just two connexin genes – Cx37 and Cx40 – would be sufficient to eliminate gap junction coupling of the arterial endothelium (Figure S4). To this end, we generated a conditional double knockout targeting these isoforms under the control of an arterial endothelial cell-specific driver – *BMX:Cre*^*ERT2*^ *Gja4*^*fl/fl*^ *Gja5*^*fl/fl*^, hereafter referred to as aEC Cx dKO (Figures S2, S5A). Tamoxifen administration yielded efficient deletion of both proteins throughout the brain’s arterial network in these mutant mice compared to *Gja4*^*fl/fl*^ *Gja5*^*fl/fl*^ controls, as demonstrated by immunostaining (Figure 3A).

To ensure that arterial endothelium gap junction coupling was abolished in aEC Cx dKO animals, we performed several functional tests. Given the difficulty of gaining physical access to CNS arterial endothelial cells, we first conducted electrophysiological and tracing experiments in the aortic endothelium, which can be readily accessed using an *en face* preparation ^28,29^. Like arterial endothelial cells in the CNS, we found that aortic endothelial cells express Cx37 and Cx40 (Figure 3B) – both of which were efficiently ablated in aEC Cx dKO mice (Figure 3C, Figure S5A). Patch-clamp recordings measured dramatically increased membrane input resistance and decreased capacitance in mutant endothelial cells relative to controls, indicative of a loss of gap junction coupling (Figure 3D, E). Consistent with this result, delivery of a small-molecule tracer, neurobiotin, through the patch pipette revealed networks of hundreds of gap junction-coupled endothelial cells in control mice. In aEC Cx dKO animals, however, neurobiotin spread was almost completely eliminated: in half of all measurements from these mice, the tracer was confined to a single cell (Figure 3F). Thus, cell type-specific deletion of Cx37 and Cx40 successfully abolished arterial gap junction coupling.

Finally, to ensure that gap junction coupling was abolished in the CNS vasculature of aEC Cx dKO mice, we performed non-invasive SERT-based tracing in these animals. To best visualize arterio-venous zonation, we examined the retinas of these animals, where the stereotyped topology of the vascular network facilitated the unambiguous identification of large arteries expressing Cx37 and Cx40. In control mice, serotonin readily spread to neighboring cells from SERT-expressing arterial endothelial cells, labeling substantial stretches of the arterial network. By contrast, in aEC Cx dKO animals serotonin uptake was sharply confined to individual SERT-expressing endothelial cells (Figure 3G), indicating that arterial coupling was effectively abolished. Importantly, as we progressed down the vascular tree to examine smaller arterioles and capillaries, we found that robust intercellular serotonin spread was preserved in aEC Cx dKO animals (Figure S5B) – demonstrating that our genetic strategy specifically abolished arterial endothelial gap junction coupling while sparing other vascular segments. Together, these experiments establish Cx37 and Cx40 as essential components for gap junction coupling in arterial endothelial cells.

### A visual stimulation paradigm reveals long-range vasodilation propagation in awake mice

**Figure 4.**
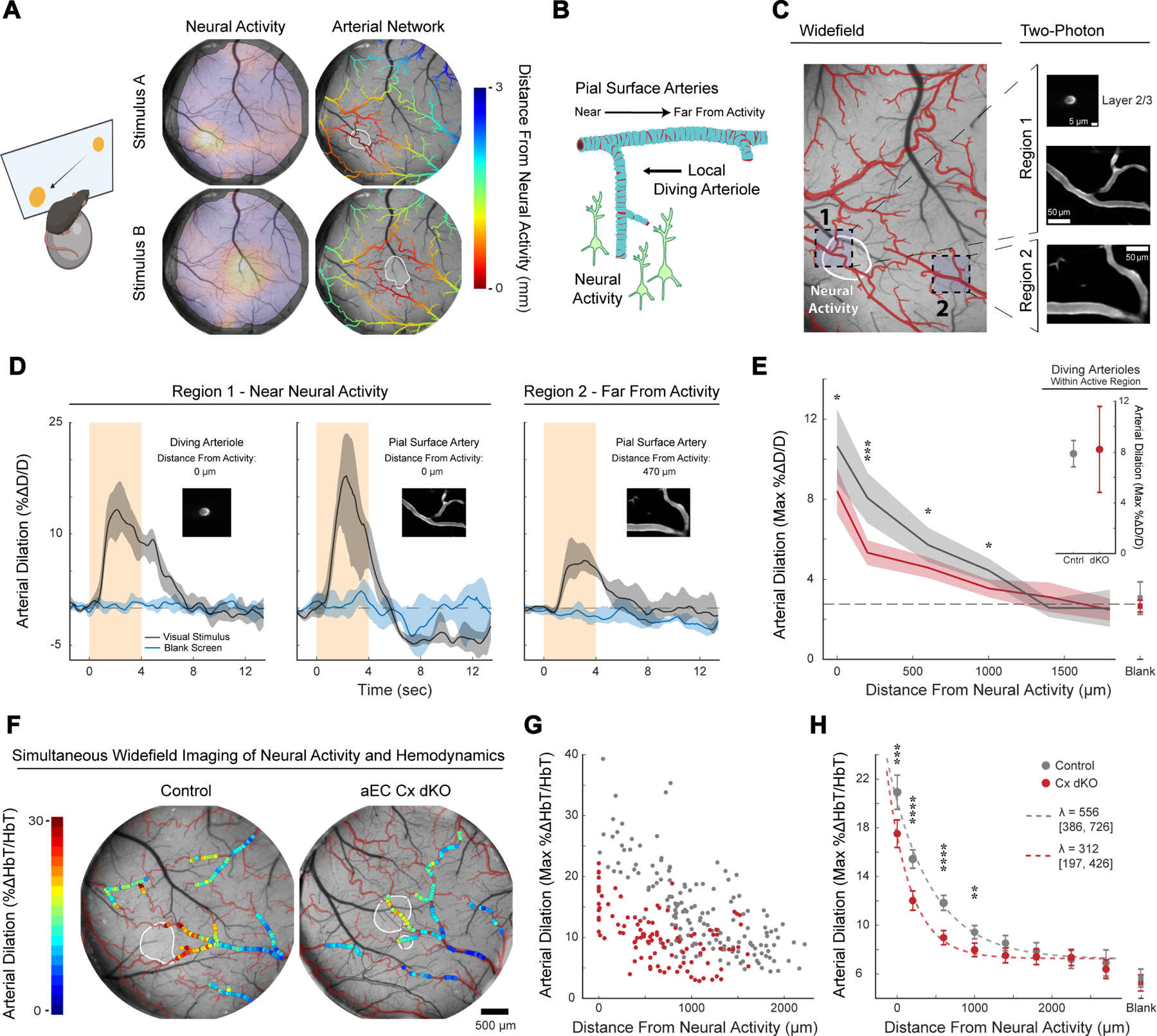
Endothelial gap junction coupling is required for long-range propagation of vasodilation during neurovascular coupling. (**A**) Left: visual stimuli are presented to head-fixed, awake mice. Center: Cranial window over contralateral visual cortex allows visualization of stimulus-evoked neural activity via widefield GCaMP imaging. Visually evoked responses are shown for two stimulus positions, with lighter colors indicating stronger activation. Right: GCaMP signal can then be binarized (white) and used to compute a distance-to-stimulus metric for positions in the arterial network (complete architecture identified via histology; see Figure S7I–J). (**B**) Schematic illustrating the anatomy of the cortical vasculature relative to neural activity. (**C**) Regions mapped in widefield microscopy (left) can be found in two-photon microscopy (right). Arterial network (red) and neural activity (white) shown. (**D**) Average stimulus-evoked (gray) and blank screen control (blue) dilation responses of arteries depicted in (**C**) (*n* = 12 trials; bootstrapped trajectories; mean ± 68% CI). Duration of stimulus presentation is demarcated in orange. (**E)** Aggregated single-vessel dilation responses of arteries at the pial surface measured using two-photon imaging, plotted as a function of distance from neural activity (linear mixed-effect model; mean ± 95% CI; *n* = 10 control [gray] / 9 connexin-knockout [red] mice except for blank-screen control data, collected from *n* = 7 / 4 of these animals). Responses recorded during blank screen presentation are pooled into a single datapoint, irrespective of position in arterial network. Dotted black line reports average dilation measured on blank-screen trials across both genotypes. See Figure S6A–B for details on how this value relates to average-response trajectories. Inset: responses of diving arterioles within active regions (linear mixed-effect model; mean ± 95% CI; *n* = 7 control / 4 connexin-knockout mice; p = 0.8399). (**F**) Simultaneous GCaMP / IOS imaging with widefield microscopy enables resolution of network-level hemodynamics. Representative examples of each genotype are shown, with average stimulus-evoked activity shown in white and full extent of the arterial network overlaid in red (also see Supplementary Movie 1). A scatterplot of these responses is shown in (**G**). (**H**) Aggregated dilation responses of pial arteries measured with widefield imaging (linear mixed-effect model; mean ± 95% CI; *n* = 9 control / 8 connexin-knockout). Blank screen as described in (**E**). The decay constant of a single exponential fitted to the data (λ) acquired from each genotype is displayed alongside a bracketed 95% CI for this metric. For quantification: * p < 0.05, ** p < 0.01, *** p < 0.001, **** p < 0.0001. Exact statistical test results for (**E, H**) reported in Supplementary Table 1.

To study the role of arterial endothelial gap junction coupling in vasodilation propagation, we needed to develop an experimental paradigm to evoke neural activity in a spatiotemporally precise manner in awake mice. We turned to the visual system because visual cortex in mammals is retinotopically organized – discrete points in the visual field drive activity in discrete locations of visual cortex ^30^. By shifting the position of a small visual stimulus on a screen presented to a head-fixed mouse, we could readily control the position of visually evoked neural activity relative to the arterial network within the brain (Figure 4A, Left). The precise location of neural activity could next be mapped via widefield imaging of neuronally-expressed GCaMP through a cranial window placed over visual cortex (Figure 4A, Center). With this information, we could assign a distance-to-stimulus metric to the entirety of the arterial network captured within the 4-millimeter cranial window (Figure 4A, Right).

Widefield GCaMP mapping allowed us to then pick specific regions of interest in two-photon microscopy with an understanding of the spatial relationship between the artery segment and the pattern of neural activity (Figure 4B, C). For example, when we imaged arterial vessels close to areas with evoked neural activity (**i.e. Region 1**), we observed robust dilation of diving arterioles and their pial surface-level counterparts (Figure 4D, Left). When we imaged arterial vessels hundreds of microns away from the center of neural activation (**i.e. Region 2**), we still observed appreciable dilation (Figure 4D, Right), demonstrating long-distance vasodilation propagation throughout the arterial network. Altogether, this visual stimulation paradigm offered unprecedented spatiotemporal control of sensory-evoked neural activity, allowing us to precisely resolve the spatial dynamics of neural activity and corresponding vascular responses across large brain areas.

### Arterial endothelial cell gap junction coupling is essential for sensory-evoked vasodilation propagation

Next, we leveraged our visual paradigm to investigate the function of arterial endothelial gap junction coupling in vasodilation propagation by comparing the neurovascular coupling responses of aEC Cx dKO and control mice. For comprehensive unbiased analysis of vasodilation propagation, we imaged single-vessel dilation responses to multiple different focal visual stimuli across many pial arteries using two-photon microscopy (examples as shown in Figure 4D). In control animals, we observed rapid, long-distance vasodilation propagation through the arterial network in response to focal visual stimuli. Stimulus-evoked vasodilatory responses gradually decayed as they spread outwards from active regions, stretching > 1mm across the surface of the brain before approaching the magnitude of spontaneous vasomotion captured in control trials where the monitor remained blank (Figure 4E, Figure S6A–B). In contrast, stimulus-evoked vasodilation in aEC Cx dKO mice decayed steeply with increasing distance from neural activity, resulting in a blunted, spatially constrained dilation response. Importantly, at sites immediately adjacent to neural activity, the vasodilation responses of mutants were intact: diving arterioles within areas of neural activity retained strong, stimulus-locked dilations statistically indistinguishable from those seen in control animals (Figure 4E, Inset). Interestingly, the pial vessels that are directly overlying neural activity (several hundred microns above these diving vessels) already exhibited dilation deficits in mutants relative to controls. This result indicates that arterial gap junctions are specifically required for vasodilation propagation in neurovascular coupling.

To further characterize the role of arterial gap junction coupling in the regulation of network-level blood flow distribution, we complemented our two-photon-based measurements with mesoscale imaging of activity-evoked hemodynamics across the entirety of the visual cortex. Using multispectral LED-based illumination, we adapted our high-resolution widefield imaging setup to simultaneously record stimulus-evoked neural activity and intrinsic optical signal (IOS) at 530nm, the isosbestic point of hemoglobin’s absorption spectra ^31^. This latter measurement was used to calculate the change in total concentration of hemoglobin (HbT), a measurement of blood content known to closely track with vessel diameter. Widefield IOS imaging revealed that sensory-evoked arterial responses were significantly blunted in aEC Cx dKO animals relative to controls, with deficits becoming more pronounced with increasing distance from neural activity (Figure 4F, G, Supplementary Movie 1). To consider these observations in aggregate, we fit a single exponential to our data and calculated a decay length constant (λ) reflecting the decay of arterial dilation with progressively increasing distance from neural activity (Figure 4H). Comparing this value across genotypes revealed that the spatial extent of stimulus-evoked vasodilation measured in connexin-knockouts was reduced to approximately half that seen in controls. Collectively, these results demonstrate that gap junction-mediated communication along the arterial endothelium is required for efficient propagation of vasodilation during neurovascular coupling.

Of note, over the course of our *in vivo* imaging experiments we observed an increase in the resting arterial diameter of mutant animals relative to controls (Fig S6C). However, resting cerebral perfusion (Figure S6D) and systemic blood pressure (Figure S6E, F) were indistinguishable between mutants and controls, indicating that the physiological consequences of this shift were limited. Importantly, this change did not underlie the distance-dependent neurovascular coupling deficits we measured in connexin-knockouts - these trends persisted even after including a correction term to our analyses to account for differences in baseline diameter (Figure S6G).

### Arterial endothelial gap junction coupling determines the speed and extent of vasodilation propagation during neurovascular coupling

To further understand the kinetics of vasodilation propagation and the precise role of endothelial gap junction coupling in this process, we virally overexpressed ChRmine – an excitatory opsin ^32^ – in neurons, allowing us to generate temporally precise, spatially focused patterns of neural activity in awake mice. Using our mesoscale widefield imaging platform, we were able to optogenetically stimulate a small population of neurons in somatosensory cortex while simultaneously measuring vascular dynamics across large portions of the arterial network. By modulating the laser power used to drive neural activity, we could reliably scale the extent of propagated vasodilation; stronger optogenetic stimulation not only produced larger arterial responses in the immediate vicinity of opsin-expressing neurons, but also gave rise to robust long-range recruitment of the arterial network (Figure S7A–C, Supplementary Movie 2).

**Figure 5.**
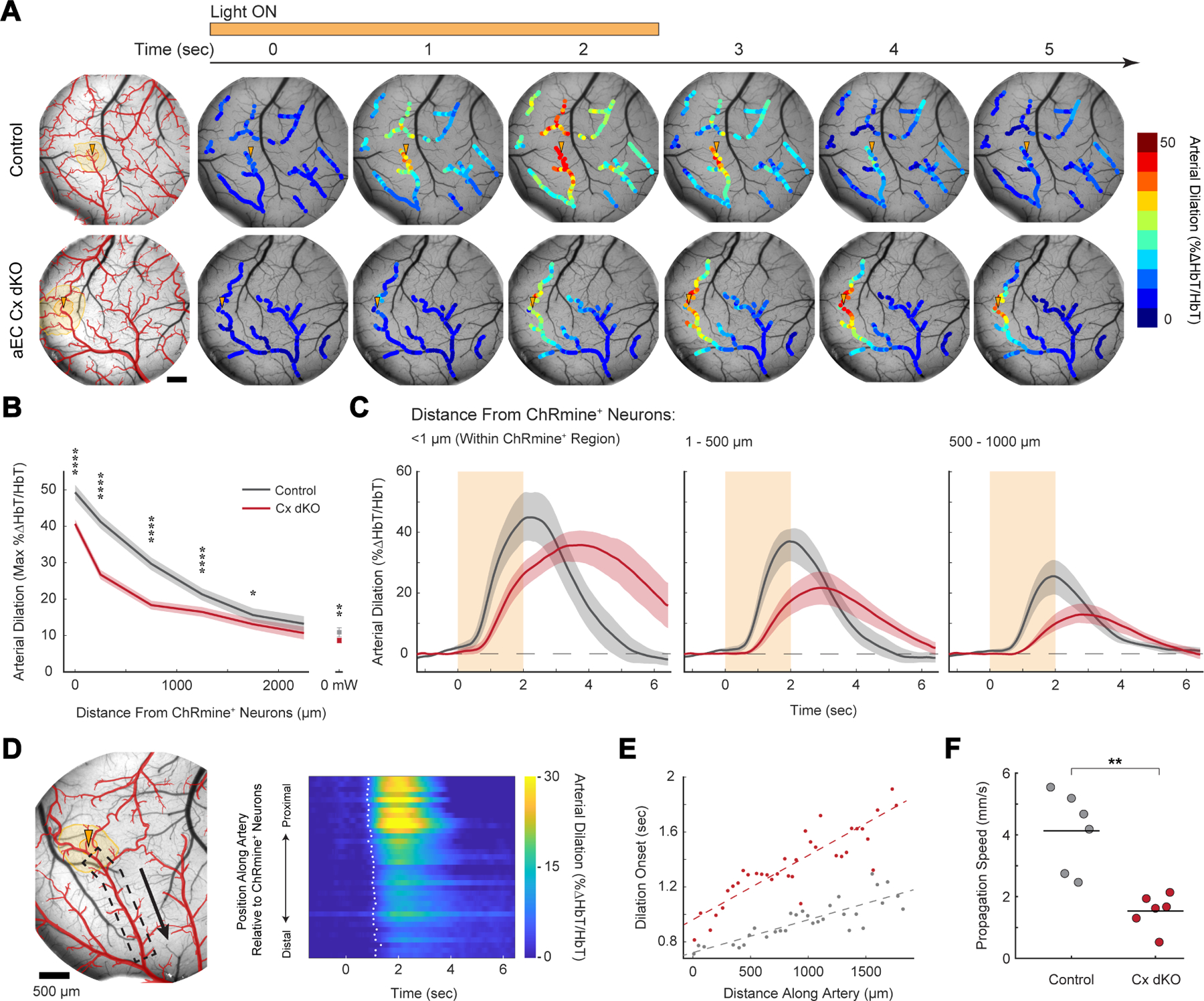
Loss of endothelial gap junction connectivity produces spatially restricted and temporally disordered neurovascular coupling. (**A**) Representative timecourses of optogenetically-evoked arterial dilation across genotypes (also see Supplementary Movie 3). In leftmost images, arterial network is demarcated in red and the extent of ChRmine expression is shown in orange. Arrowhead demarcates the center of transduction. See Figure S7I–J for additional details. Scale bar shown is 500µm. (**B**) Aggregated dilation responses plotted as a function of distance from ChRmine expression (linear mixed-effect model; mean ± 95% CI). Control (0 mW/cm^2^) responses are pooled into a single datapoint, irrespective of position in arterial network. Residual dilation observed in these trials likely reflects a combination of spontaneous vasomotion and low-grade opsin activation by wavelengths used for IOS imaging. (**C**) Arterial dilation trajectories at progressively increasing distances from ChRmine-expressing neurons (hierarchical bootstrap; mean ± 95% CI ). Duration of light pulse is demarcated in orange. (**D**) Strategy for calculating velocity of propagated vasodilation. Left: in each animal, an arterial segment ramifying into the ChRmine-expressing region (boxed region) was selected for analysis. Right: a dilation onset time for that vessel was calculated at progressively increasing distances from ChRmine transduction. (**E**) A propagation speed was then computed as the slope of the best-fit line describing the relationship between dilation onset and physical displacement along the artery. Representative examples of a control and connexin-knockout animal are shown. (**F**) Propagation speeds measured across genotypes (two-sample t-test; p = 0.0010). Vasodilation propagated through the arterial network at 4.13 ± 0.52 mm/sec in controls versus 1.53 ± 0.23 mm/sec in connexin-knockouts (mean ± s.e.m.). High-power optogenetic stimulus (10 mW/cm^2^) was used for all data shown; *n* = 6 animals of each genotype analyzed. For quantification: * p < 0.05, ** p < 0.01, **** p < 0.0001. Exact statistical test results for (**B**) reported in Supplementary Table 1.

Using this approach, we next compared the optogenetically-evoked neurovascular coupling responses of aEC Cx dKO animals and controls (Figure 5A, Supplementary Movie 3). Recapitulating results we obtained using naturalistic visual stimuli, we observed that gap junction-deficient mutants recruited substantially weaker arterial dilation across shorter stretches of the pia network (Figure 5B, Figure S7D). However, beyond this difference in magnitude, strikingly dysregulated kinetics were readily apparent in the arterial responses of mutant animals (Figure 5C). Vasodilation in connexin-knockouts exhibited dramatically slowed onset, time-to-peak, and resolution relative to controls – such that substantial arterial dilation persisted > 4.5 seconds following the cessation of optogenetic stimulation (Figure S7E–H).

Taking advantage of the spatiotemporal precision of our optogenetic stimulation, we next calculated the speed at which vasodilation propagated along arteries in mutants and controls. Across genotypes, we found reproducibly that dilations emanated outwards from stimulated areas of the brain with detectable lag, such that responses first appeared proximal to ChRmine-expressing regions and subsequently spread to encompass large stretches of arterial network. By measuring the onset of vasodilation along arteries ramifying from stimulated sites (Figure 5D, E) we calculated a propagation velocity of ~4 mm/sec in control animals, similar to previously reported speeds obtained in acute, anesthetized preparations ^11,33,34^. By contrast, propagation of vasodilation was markedly slower in aEC Cx dKO animals, proceeding at only ~1.5 mm/sec (Figure 5F). Collectively, these results demonstrate endothelial gap junction coupling determines the speed of propagated vasodilation and maximizes the extent of stimulus-evoked perfusion during neurovascular coupling.

### Arterial gap junction coupling enables flexible scaling of neurovascular coupling to meet energetic demand

**Figure 6.**
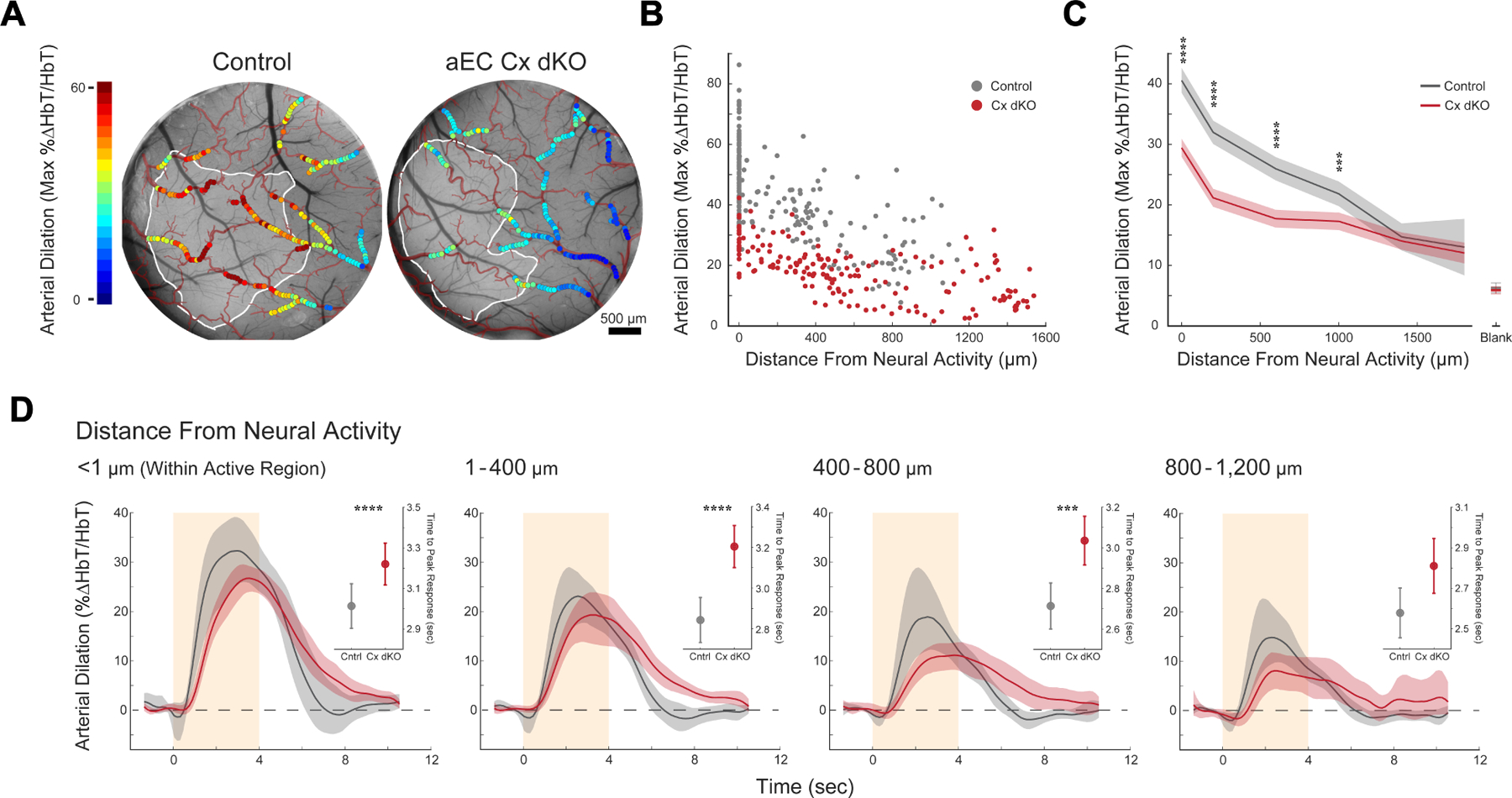
Acute metabolic demand evokes long-range arterial dilation via gap junction-mediated signaling. (**A**) Representative examples of stimulus-evoked responses captured via simultaneous GCaMP / IOS widefield imaging (also see Supplementary Movie 4). A scatterplot of these responses is shown in (**B**). (**C**) Aggregated dilation responses plotted as a function of distance from neural activity (linear mixed-effect model; mean ± 95% CI). Responses recorded during blank screen presentation are pooled into a single datapoint, irrespective of position in arterial network. (**D**) Arterial dilation trajectories at progressively increasing distances from stimulus-evoked neural activity (hierarchical bootstrap; mean ± 95% CI). Duration of stimulus presentation is demarcated in orange. Insets show time-to-peak dilation response calculated across genotypes for each bin (linear mixed-effect model; mean ± 95% CI). Statistical test results are as follows: [<1µm; p < 0.0001], [1–400µm; p < 0.0001], [400–800µm; p = 0.0005], [800–1,200µm; p = 0.0987]. For all quantification shown: *n* = 3 control / 5 connexin-knockout mice were analyzed; *** p < 0.001, **** p < 0.0001. Exact statistical test results for (**C**) reported in Supplementary Table 1.

Propagated vasodilation is thought to be particularly important during intense bouts of local activity. In these cases, dilations limited to local vessel segments are, by themselves, insufficient to answer metabolic demand ^9,35^. To create such a situation with a naturalistic stimulus, we presented mice with a full-screen visual stimulus occupying >83° of visual space. In contrast to the focal visual stimuli (18°) used in our prior experiments, this full-screen stimulus drove neuronal activation across the entirety of visual cortex. Moreover, a full-screen stimulus produced dramatically enhanced vasodilation relative to the smaller stimuli, consistent with the notion that neurovascular coupling responses flexibly scale to meet energetic demand (Figure 6A, B, Supplementary Movie 4).

Similar to our results using focal visual stimulation, aEC Cx dKO mice exhibited distance-dependent vasodilation deficits compared to controls following full-screen stimulation. However, the difference in the magnitude of dilation between control and mutant mice was markedly exacerbated following presentation of the more intense stimulus (Figure 6C). Furthermore, the mutants’ response to the full-screen stimulus revealed a perceptible slowing of neurovascular coupling kinetics, similar to our results with optogenetic stimulation. Controlled for distance from neural activity, the average dilation trajectories of arteries from aEC Cx dKO animals exhibited quantifiably slower time-to-peak, as well as apparent delays in both the initiation and resolution of vasodilation (Figure 6D). These results indicate that arterial gap junction mutants are unable to adequately scale neurovascular coupling to meet energetic demand.

## Discussion

**Figure 7.**
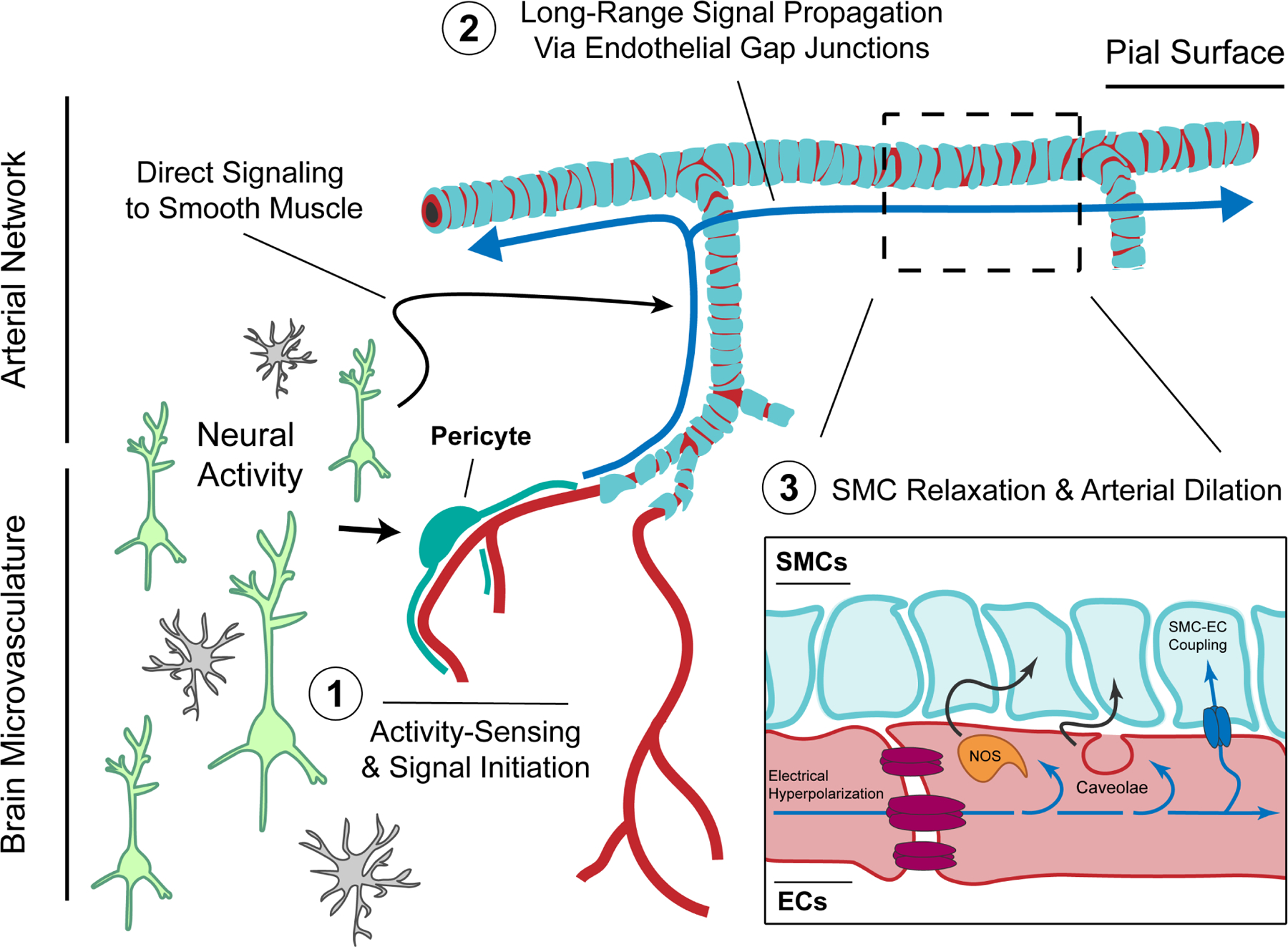
Proposed model for endothelial gap junction-mediated signaling during neurovascular coupling. Upon neural activation, neuron (green) and astrocyte (grey) derived molecules engage the nearby cerebrovasculature as a ‘signaling highway’ to rapidly produce dilation across large stretches of the arterial network: **(1)** The pericytes and capillary endothelial cells (ECs) that constitute the brain microvasculature sense local byproducts of neural activity – such as extracellular potassium or adenosine – and transduce this event into electrical hyperpolarization using specific channels and receptors ^14–17,36,37,39^. This electrical signal is subsequently (**2**) propagated upstream to - and throughout - the arterial network via endothelial-endothelial gap junctions. As hyperpolarization travels along the arterial endothelium, it (**3**) recruits smooth muscle cell (SMC) relaxation and vasodilation through several mechanisms including nitric oxide release ^65^, caveolae-mediated signaling ^19^, and/or direct charge transfer ^66,67^ via myoendothelial gap junctions (see Figure S1B). Upstream arterial dilation in turn produces dramatically increased capillary blood flow in the vicinity of neural activity. In parallel, particularly in cases where neural activity and arterial segments are closely apposed, vasoactive signals released by neurons and astrocytes act directly on smooth muscle cells and elicit vasodilation ^47,49–52^.

Our data demonstrate that CNS endothelial cells are functionally coupled by gap junctions and serve as a signaling highway to enable rapid vasodilation propagation through arteries during neurovascular coupling. By identifying connexin isoforms in CNS endothelial cells, generating mouse models, and developing visual and optogenetic imaging strategies, we establish that arterial gap junction coupling determines the speed and spatial extent of vasodilation propagation following neural activity. These long-range, propagated responses are especially essential in the context of intense neural activation, where they enable flexible scaling of stimulus-evoked blood flow to match energetic demand. Our findings fill an important gap of knowledge regarding the basic mechanism of neurovascular coupling. Previous work has shown that microvascular endothelial cells ^14–17,36,37^ and pericytes ^16,17^ are able to sense local neural activity via specific channels and receptors. Recent work from our lab and others have shown that arterial endothelial cells play an indispensable role in recruiting the smooth muscle relaxation that leads to increases in downstream perfusion seen during neurovascular coupling ^18,19^. Here we demonstrate that endothelial connexins are critical for relaying the signals sensed by the microvasculature to distal upstream arteries, allowing for acute coupling of blood flow to neural activity. Therefore, the vascular system acts as a major communication route that relays signals from neurons to smooth muscle cells (Figure 7).

What signals are passed through these gap junctions? The speed of vasodilation propagation suggests that the signal is electrical in nature. Recent studies have revealed that brain capillaries are equipped with channels (e.g. K_ir_2.1 ref. ^14^ and K_ATP_ refs. ^16,17,38,39^) able to transduce byproducts of neural activity into electrical hyperpolarization. Similarly, electrophysiological recordings from isolated peripheral arteries have shown that endothelial cells can be hyperpolarized by local vasoactive stimuli and rapidly propagate this signal to neighboring endothelia ^40^. How such electrical signals might be shaped as they travel throughout the complex, branched network of the cerebrovasculature during neurovascular coupling remains an open question – one perhaps best answered using emerging voltage imaging technologies ^41^.

While loss of arterial endothelial gap junction coupling markedly blunts the neurovascular coupling response, it does not completely abrogate activity-induced dilation. The residual response is most likely due to a parallel or redundant mechanism; one with slower kinetics operating over smaller spatial scales. One possibility is that in the absence of a strongly gap junction-coupled endothelial conduit, vasodilatory signals might be propagated along the muscular layer of arteries via smooth muscle cell (SMC)-SMC gap junctions. While smooth muscle cells are thought to form much weaker gap junction contacts than endothelial cells ^42,43^, studies in the peripheral vasculature have indicated that vasomotor signals can in principle be propagated along the muscular wall of arteries without endothelial involvement ^44–46^. Our own data supports the existence of such smooth muscle gap junctions in the cerebrovasculature: regular punctate Cx37 signals at smooth muscle contacts running orthogonal to pial arteries become evident following ablation of endothelial Cx37 in our mutant mice (Figure 3A). Additionally, it is possible that local dilation responses seen in connexin-knockout animals could stem from direct neuronal or astrocytic signaling to smooth muscle cells, bypassing an endothelial conduit in situations where the cells are closely apposed ^47–52^ (Figure 7).

We show that while all endothelial cells of the brain express gap junctions, their molecular composition and coupling strength follow an arterio-venous axis. This zonation has implications for the direction of signal propagation from the microvasculature preferentially towards arteries. To wit, the properties of individual connexin isoforms vary substantially ^53,54^. For example, the unitary conductance of channels composed of Cx37 (expressed in arteries) is nearly an order of magnitude higher than those formed by Cx45 (expressed in veins) ^55,56^. These findings suggest that isoform-switching along the arterio-venous axis indeed has functional consequences, shaping the electrotonic environment to favor efficient capillary-to-artery signal transmission. In line with this idea, we find that organization of Cx37 expression is markedly relaxed in the lung microvasculature (Figure S3H–I), indicating that sharp arterial zonation of high-conductance isoforms might be dispensable in visceral organs which lack a need for spatially precise blood flow control.

Given the broad expression and diverse functions of gap junctions in cell types across the body ^57–60^, the organizing principles and non-invasive tracing methodology described in this work might be widely applied. For example, the distinct connexin zonation along the arterio-venous axis is strikingly reminiscent of the cardiac conduction system, where variation in connexin expression appears important for shaping electrical properties of the cellular network ^61,62^. This kind of domain-specific utilization of connexin isoforms might be a shared feature of many electrically coupled networks in the body. Moreover, as an expanding repertoire of engineered viral vectors enables access to increasingly diverse cell types ^63^, our *in vivo* gap junction tracing system can likely be used to evaluate the coupling properties of a wide range of systems in a cell type-specific manner at scale.

Taken together, our findings demonstrate the importance of gap junction-mediated signaling along the vasculature in shaping blood flow responses to neural activity, a discovery with at least two consequential implications for human brain health. First, since blood flow is often used as a proxy for neural activity in brain imaging applications, disease contexts that alter endothelial gap junction coupling may lead to mistaken interpretation of neural activity. Second, blunting of neurovascular coupling is increasingly believed to contribute to neurodegeneration. The endothelial signaling axis described in this work may therefore be a promising therapeutic target in this regard ^6,7^.

### Limitations of the Study

Throughout our *in vivo* imaging experiments, we report vasodilation as a function of distance from neural activity (Figures 4 & 6). We define neural activity with a widefield recording of change in neuronal GCaMP fluorescence above a given threshold. This presents two limitations. First, widefield microscopy is heavily weighted to the upper layers of cortex and therefore cannot reliably measure activity in cortical layer IV or below. Second, even in the absence of external stimuli there is considerable cortical neural firing in awake animals. As a result, our threshold necessarily ignores sparse levels of neural activity. Therefore, we are unable to make conclusive statements about the potential contribution of low-level activity to propagated vasodilation.

Our estimations of the velocity at which vasodilation propagates throughout the arterial network are based on calculations of the onset time of dilation (Figure 5D–F). Though this approach has been used by a number of other groups ^33,34,64^, it is inherently limited by strength of the vasodilation ‘signal’ relative to baseline fluctuations in diameter and highly influenced by the exact methodology used to calculate onset ^11^. As a result, the absolute propagation velocities reported in this study are subject to uncertainty and should be interpreted as such. However, we note that any systematic biases in this calculation should apply equally across genotypes and exert minimal influence on the most salient feature of this data – a clear difference in propagation velocity between aEC Cx dKO animals and controls.

## STAR★Methods

### Experimental Model and Study Participant Details

#### Animals

All procedures were approved by the Harvard Institutional Animal Care and Use Committee (IACUC). A mix of male and female mice were used in the study. No sex-specific effects were noted. Data was collected from adult mice ranging from 8 – 41 weeks old.

The following mouse lines were used: Cx37^Flox^, Cx37^LacZ^, Cx37^KO^ (Ref ^79^ – JAX 025698), Cx40^Flox^ (Ref ^75^), Cx40^GFP^ (Ref ^24^), Cx43^Flox^ (Ref ^78^ – JAX 008039), Cx43^Flox-KI-CFP^ (Ref ^25^), Cx45^Flox-KI-GFP^ (Ref ^26^), Bmx:Cre^ERT2^ (Ref ^68^), Tie2:Cre (Ref ^80^ – JAX 008863), Actb:Cre (Ref ^81^ – JAX 019099), R26:CAG-Sun1/sfGFP (Ref ^82^ – JAX 021039), Ai65F (Ref ^83^ – JAX 032864), R26:FLPe (Ref ^84^ – JAX 009086), C57BL/6NCrl (Charles River 027), and C57BL/6J (JAX 000664). Because transgene insertion in the Cx40^GFP^ reporter disrupts the endogenous Cx40 coding sequence ^88^, mice homozygous for the allele are referred to as Cx40^KO^ in this study.

Unless otherwise stated, transgenic mice were obtained from Jackson Laboratories. Cx37^KO^ was generously shared by Dr. Ayako Makino. Cx40^Flox^ was generously shared by Dr. Gregory Morley with permission from Dr. Toon van Veen. Cx40^GFP^ was generously shared by Dr. Jason Butler with permission from Dr. Lucile Miquerol. Cx43^Flox-KI-EFCP^ was obtained from the European Mouse Mutant Archive (EMMA ID 06788). Cx45^Flox-KI-EGFP^ was generously shared by Dr. Marla Feller. BMX:Cre^ERT2^ was generously shared by Ralf Adams.

To induce Cre^ERT2^ recombination, adult mice (≥8 weeks) received a 1mg dose of tamoxifen (Sigma T5648) via intraperitoneal injection for 5 sequential days. For functional experiments, both *BMX:Cre*^*ERT2*^ *Gja4*^*fl/fl*^ *Gja5*^*fl/fl*^ animals and *Gja4*^*fl/fl*^ *Gja5*^*fl/fl*^ controls were treated with tamoxifen.

### Method Details

#### Genotyping.

Tail-snip biopsies were obtained from mice at weaning and digested 2–48 hours at 55° C in 100µL GNT-K buffer consisting of: 50mM KCl, 1.5mM MgCl_2_, 10mM Tris HCl pH 8.5, 0.01% Gelatin (Sigma G1393), 0.45% NP-40, 0.45% Tween-20 and 1:100 Proteinase K (NEB P8107S) ^89^. Next, a 10-minute 95° C incubation was performed to heat-inactivate the enzyme. 1µL of the digest was used as template for downstream genotyping with Phire Green Hot Start II DNA Polymerase (Thermo Fisher F124L). Genotyping primers and expected band sizes are described in Supplementary Table 2

#### Generation of Cx37^flox^ and Cx37^LacZ^ Mouse Lines.

A ‘knockout-first’ conditional allele embryonic stem-cell line – Gja4^tm1a(KOMP)Wtsi^ (IMPC Project 23979; KOMP ID 056038), C57BL/6N-A^tm1Brd^ strain – was obtained from the International Mouse Phenotyping Consortium (IMPC) ^90^. Chimera generation was achieved via microinjection by the Gene Manipulation & Genome Editing Core in the F.M. Neurobiology Center at Boston Children’s Hospital. Chimeric males were mated with C57BL/6NCrl females (Charles River, 027) and F1 progeny were screened for germline transmission via long-range PCR using a forward primer positioned outside the construct (5’-AAGGGTGTGGGATGAGTTGAAAAACG-3’) and a reverse primer positioned within the knock-in cassette (5’-GGTGGTGTGGGAAAGGGTTC-GAAG-3’). A single founder was identified and crossed with Actb:Cre (Ref ^81^ – JAX 019099) and R26:FLPe (Ref ^84^ – JAX 009086) females to generate Cx37^flox^ and Cx37^LacZ^ alleles, respectively. Recombinase elements were removed in subsequent crosses. Predicted sequences are reported in Supplementary Data 1.

To our surprise, we observed impaired fertility in Cx37^fl/fl^ females reminiscent of infertility previously reported in Cx37-null animals ^79^. We performed two controls to verify we had not inadvertently generated a null allele. First, we isolated the Cx37^flox^ allele (5’- CTG-GCTGGCGTTTCCTGC-3’ and 5’- GCAAGTCTGTGCAAAA TAACTGCCT-3’ were used to generate a ~3kb product) and Sanger-sequenced the CDS-containing second exon. The sequence was a perfect match to the wild-type allele (Supplementary Data 1). Second, we verified normal Cx37 expression and localization in Cx37^fl/fl^ animals – as well as susceptibility to Cre-induced deletion (Figure S2C). Collectively, these results indicate that while the Cx37^flox^ allele encodes a functional protein, its expression is hypomorphic in the context of oocyte development. We speculate this disruption could stem from incorporation of a loxP site within the 3’ UTR. To accommodate this hypomorphism, we used Cx37^fl/+^ female breeders to produce the mutant animals utilized in this study. These animals – as well as Cx37^fl/fl^ males – exhibited normal fertility.

#### Cloning.

The plasmids involved in non-invasive SERT tracing were produced as follows: pAAV-CAG-fDIO(SERT-HA)-miR122-WPRE-pA was generated by first producing a fDIO(SERT-HA)-miR122 intermediate product via iterative assembly with NEBuilder HiFi DNA Assembly Cloning Kit (NEB E5520S) using hSERT pcDNA3 (Addgene #15483, a gift from Randy Blakely ^91^), pAAV-Ef1a-fDIO mCherry (Addgene #114471, a gift from Karl Deisseroth), and pAAV-CAG-SV40NLSf-GFP-3xmiR122-WPRE-HGHpA (Addgene #183775 ^22^) as starting materials. The fragment was then inserted into the pAAV-CAG-SV40NLSf-GFP-3xmiR122-WPRE-HGHpA backbone via restriction-ligation cloning (NEB M0202S) using KpnI (NEB R3142S) and HindIII (NEB R3104S). pAAV-CAG-FLPo-miR122-WPRE-pA was similarly generated from pCAG-Flpo (Addgene #60662, a gift from Massimo Scanziani ^92^) and the pAAV-CAG-SV40NLSf-GFP-3xmiR122-WPRE-HGHpA backbone via restriction-ligation cloning using KpnI and EcoRI (NEB R3101S).

The Cx40◦GFP fusion construct used for heterologous overexpression of Cx40 in HEK293T cells was generated by digesting pAcGFP1-N2 (Clontech 632483) with SmaI (R0141S). The mouse Cx40 CDS was then amplified from a cloning vector (Sino Biological MG51614-U) and inserted into this backbone via blunt-end ligation.

#### *In Vitro* Experiments.

HEK293T cells were cultured in Dulbecco’s Modified Eagle Media (DMEM; Thermo Fisher 11995065) supplemented with 1% Penicillin/Streptomycin (Thermo Fisher 15140122) and 10% fetal bovine serum (R&D Systems S11150) For all experiments, cells were grown in 48-well plates on poly-L-lysine coated (Sigma Aldrich P4707) 12mm coverslips (Electron Microscopy Sciences 72195–12) and analyzed two days after transfection with Lipofectamine 2000 (Invitrogen 11668019) and Opti-MEM (Thermo Fisher 31985062).

For non-invasive gap junction tracing, cells were cultured in media supplemented with dialyzed fetal bovine serum (≤ 10,000 Da cut-off; Thermo Fisher A3382001) to minimize serotonin (~175 Da) uptake prior to introduction of an exogenous pulse. Cells were transfected with a 250ng / 0.1ng cocktail of pAAV-CAG-fDIO(SERT-HA)-miR122-WPRE-pA and pAAV-CAG-FLPo-miR122-WPRE-pA. Two days later, serotonin (Cayman Chemical 14332) dissolved in DMSO was added to each well to reach a final concentration of 400nM. Carbenoxolone (Cayman Chemical 18240) and escitalopram (Tocris Bioscience 4796) were dissolved in water or DMSO and added one hour prior to the serotonin pulse to reach final concentrations of 200µM or 5µM, respectively. Following serotonin application, cells were incubated for 20 minutes at 37°C and fixed in 4% PFA for 30 minutes at room temperature.

Following fixation, cells were washed 3x in PBS and blocked for 1 hour at room temperature in a 5% Donkey Serum 0.1% PBST solution. Primary antibodies were made up in blocking solution and incubated overnight at 4°C, with the exception of anti-HA immunostaining which was performed in conjunction with secondary staining. Cells were then washed 3x in 0.1% PBST and incubated in secondary antibodies made up in blocking solution for one hour at room temperature. Finally, cells were washed 3x in 0.1% PBST, 1x in PBS, and mounted on slides.

The following primary antibodies were used for immunocytochemistry: Mouse anti-HA Tag ◦ Alexa Fluor 555 (Invitrogen 26183-A555; 1:200), Rabbit anti-Cx43 (Sigma Aldrich C6219; 1:10,000), and a purified Rabbit anti-Serotonin antibody (see Serotonin Antisera Clearing). Subsequently, the following secondary antibodies were used: Donkey anti-Rabbit ◦ Alexa Fluor 488 (Jackson ImmunoResearch 711-545-152; 1:250) and Donkey anti-Rabbit ◦ Alexa Fluor 647 (Jackson ImmunoResearch 711-605-152; 1:250). Actin filaments were labeled using a phalloidin TRITC conjugate, Actin Red 555 Ready Probes Reagent (Invitrogen R37112).

#### Immunohistochemistry.

Mice were deeply anesthetized with an intraperitoneal injection of a ketamine / xylazine solution and transcardially perfused with ~15 mL of room-temperature PBS followed by ~20 mL of ice-cold 4% PFA using a peristaltic pump set to a flow rate of ~9–10 mL/min. Tissues were subsequently collected and processed as follows:

##### Tissue clearing.

Brain was removed post-fixed overnight in 4% PFA at 4°C with agitation. Samples were washed 3x with PBS and a small piece of cortex (~5mm^3^) was isolated with a razor blade. Tissue clearing and whole-mount immunolabeling were subsequently achieved via the U.Clear method previously described in *Pfau et al.* (ref. ^93^).

##### Pia vasculature.

Brain was dissected out of the skull and partially immersed in PBS in a glass dish. A razor blade was used to make a cut along the sagittal midline followed by a cut along the horizontal axis to separate each hemisphere into dorsal and ventral pieces. Dorsal-facing brain samples were then transferred into a 48-well plate and post-fixed in 4% PFA on ice for 30 minutes. To capture images, brain samples were placed dorsal face-down in a 2-well glass bottom slide (Ibidi 80287) partially filled with PBS such that the pia vasculature faced the objective on an inverted microscope. The concentration of PFA used for perfusion and post-fixation was reduced to 1% in order to detect Cx37 and Cx40 in brain (Figures 3A, S2C, S4A–B). Following immunostaining, brain samples were placed dorsal face-down in glass well-slide (Ibidi 80287) partially filled with PBS such that the pia vasculature faced the objective on an inverted microscope for imaging.

##### Aorta *en face*.

Thoracic aorta was grossly dissected and immersed in PBS. Fat, connective tissue, and arterial branches were subsequently removed via fine dissection. A single cut was made lengthwise along the vessel to expose the lumen. A post-fixation step was omitted. Following immunostaining, samples were mounted *en face* on slides endothelium-side up.

##### Retinas.

Eyes were removed and briefly post-fixed in room-temperature 4% PFA for 5–10 minutes. Retinas were then isolated via fine dissection and further post-fixed in room-temperature 4% PFA for 30 minutes. Following immunostaining, retinas were flat-mounted on glass slides.

For all whole-mount preparations samples were washed 3x with PBS, blocked with a 10% Donkey Serum / 0.5% PBST (Triton X-100) solution for 2 hours at room temperature, and then incubated with primary antibodies made up in blocking solution overnight (retina, aorta) or for 48 hours (pia vasculature) at 4°C with agitation. The samples were next washed 3x with 0.5% PBST and incubated in secondary antibodies for 1 hour at room temperature (retina, aorta) or for an additional 48 hours (pia vasculature) at 4°C. Finally, samples were washed 3x with 0.5% PBST and 1x with PBS.

##### Tissue sections.

Organs were post-fixed in 4% PFA at 4°C for 4 hours on ice. For anti-CFP staining in the *Tie2:Cre Gja1*^*fl-KI-CFP*^ reporter (Figure 2E) and anti-ßGal staining in brain and lung (Figures 2D, S2E–F, S3H–I) the concentration of PFA used for perfusion and post-fixation was reduced to 1% and post-fixation was extended overnight with agitation. Samples were next washed 3X with PBS to remove residual PFA. For cryosections, samples were incubated in a 30% sucrose solution at 4°C overnight and then frozen in NEG-50 (Richard-Allan Scientific 6502). For aortic sections (Figures 3B, S2E [aorta]), both post-fixation and cryoprotection steps were omitted. Sections were cut using a cryostat (Leica CM3050 S) at either 20µm (Figure 3B, S2E [aorta]) or 30µm (Figures 2D, S2E [brain and lung] & F, S3C–D, F). Alternatively, 50µm (Figure S7C) or 60–80µm (Figures 1C, 2E) vibratome sections were cut immediately following PBS washes using a vibrating blade microtome (Leica VT1000S).

For immunostaining, cryosections were washed 3X in PBS, permeabilized in 0.5% PBST for 10min, and then blocked with a 5% donkey serum / 0.1% PBST solution for 1hr at room temperature. Sections were subsequently incubated within primary antibodies made up in blocking solution overnight at 4°C, washed 3x with 0.1% PBST, and incubated with secondary antibodies made up in blocking solution for 1hr at room temperature. Finally, sections were washed 3x with 0.1% PBST, 1x with PBS, and coverslipped for imaging. Vibratome sections were incubated in solutions containing 0.5% PBST and immunolabeled with secondary antibodies overnight at 4°C with agitation but otherwise treated identically.

The following primary antibodies were used: Rabbit anti-Cx37 (Invitrogen, 40–4200; 1:100), Rabbit anti-Cx40 (Invitrogen 36–5000; 1:100), Mouse anti-Claudin5 ◦ Alexa Fluor 488 (Invitrogen 352588; 1:200), Mouse anti-α Smooth Muscle Actin ◦ FITC (Sigma F3777; 1:250), Mouse anti-α Smooth Muscle Actin ◦ Cy3 (Sigma C6198; 1:500), Mouse anti-α Smooth Muscle Actin ◦ Alexa Fluor 647 (Santa Cruz Biotechnology sc-32251; 1:100), Rabbit anti-ßGalactosidase (MP Biomedicals 085597-CF; 1:1000 in brain and lung, 1:5000 in aorta), Rabbit anti-Cx43 (Sigma C6219; 1:500), Rabbit anti-ERG ◦ Alexa Fluor 488 (Abcam ab196374; 1:100), Goat anti-CD31 (R&D Systems AF3628; 1:100), Rabbit anti-GFP ◦ Alexa Fluor 488 (Invitrogen A-21311; 1:200), Rat anti-CD140b/PDGFRß (Invitrogen 14-1402-82; 1:100), Mouse anti-HA Tag ◦ Alexa Fluor 555 (Invitrogen 26183-A555; 1:100), and purified Rabbit anti-Serotonin (Sigma S5545; see Serotonin Antisera Clearing). Retinal vasculature was labeled using Isolectin GS-IB4 conjugated to Alexa Fluro 488, Alexa Fluor 568, or Alexa Fluro 647 (Invitrogen I21411, I21412, I32450; 1:100). The primary antibody concentrations used for whole-mount immunolabeling via the U.Clear method were double those listed above.

The following secondary antibodies were used at 1:250 in all applications unless otherwise stated: Donkey anti-Rabbit ◦ Alexa Fluor 488 (Invitrogen A-21206), Donkey anti-Rabbit ◦ Cy3 (Jackson ImmunoResearch 711-165-152), Donkey anti-Rabbit ◦ Alexa Fluor 647 (Jackson ImmunoResearch 711-605-152), Donkey anti-Goat ◦ Alexa Fluor 488 (Jackson ImmunoResearch 705-545-147), Donkey anti-Goat ◦ Alexa Fluor Cy3 (Jackson ImmunoResearch 705-165-147), Donkey anti-Goat ◦ Alexa Fluor 647 (Jackson ImmunoResearch 705-605-147), Donkey anti-Rat ◦ Alexa Fluor 647 (Jackson ImmunoResearch 712-605-153).

Representative images were acquired with a Leica TCS SP8 confocal microscope. Relevant modifications to the immunostaining protocol were as follows: (1) Mouse anti-Claudin5 (Invitrogen 35–2500) and Rabbit anti-ERG (Abcam ab92513) were directly conjugated to fluorophores using Alexa Fluor^™^ 647 Antibody Labeling Kit (Thermo Scientific A20186) and subsequently used at 1:100 in order to achieve co-labeling with GFP in *BMX:Cre*^*ERT2*^ *R26*^*CAG-Sun1/sfGFP*^ tissues (Figure S5A). (2) The ECFP fluorophore in *Tie2:Cre Gja1*^*fl-KI-CFP*^ reporter animals was difficult to detect in brain and required specialized conditions. Amplification of ECFP was found to be highly fixation-dependent; as a result, animals were perfused with 1% PFA and post-fixed in 1% PFA at 4°C overnight. Samples were subsequently washed with PBS, embedded in 2% low melting-temperature agarose, and cut into 60µm thick sections using a vibratome. ECFP fluorescence was sequentially amplified with 1:200 Rabbit anti-GFP ◦ Alexa Fluor 488 (Invitrogen A-21311) and 1:500 Donkey anti-Rabbit ◦ Alexa Fluor 488 (Invitrogen A-21206). A notable astrocytic leak signal was apparent in many regions of the brain, but comparatively reduced in cortex. (3) To achieve co-labeling with two antibodies raised in rabbit (Rabbit anti-ERG and Rabbit anti-ßGal) the immunostaining protocol was modified as follows. Standard procedures were followed to immunolabel samples with Rabbit anti-ßGal and a corresponding anti-Rabbit secondary antibody. Following this secondary incubation, slides were washed 5x with 0.1% PBST, blocked for 1 hour at room temperature in a 10% Normal Rabbit Serum / 0.1% PBST solution, further washed 5x with 0.1% PBST, and blocked for an hour at room temperature in standard blocking solution (5% Normal Donkey Serum / 0.1% PBST). Finally, slides were incubated in Rabbit anti-ERG ◦ AF488 made up in blocking solution overnight at 4°C, washed 3x with 0.1% PBST & 1x with PBS, and mounted. Absence of cross-reactivity is verified in Figure S2F.

#### Whole-Mount X-Gal Staining.

Perfusion was performed as described using ice-cold 2% PFA. Whole brains were carefully dissected out of the skull and post-fixed in 2% PFA on ice for 30 minutes. Eyes were removed and post-fixed in 4% PFA at room temperature for 5 minutes. Retinas were finely dissected and post-fixed further in 2% PFA at room temperature for 30 minutes. From this point forward, brains and retinas were treated identically. Samples were washed 3x with PBS and 3x30 minutes with Permeabilization Solution (PBS containing 0.02% Nonidet P-40; 0.01% Sodium Deoxycholate; 2mM MgCl2). Samples were then transferred to Staining Solution (Permeabilization Solution containing 5mM Potassium Ferricyanide; 5mM Potassium Ferrocyanide; 1 mg/mL X-Gal) and incubated at 37◦C for 4 hours. Finally, samples were washed 3x with PBS, fixed in 4% PFA at room temperature for 1 hour, washed 3x with PBS once again, and bleached for 1 hour in 70% ethanol. Images of whole brains were taken with a Nikon D5200 camera with a Nikon AF-S NIKKOR 24–85mm f/3.5–4.5G ED VR lens. Retinas were flat-mounted on glass slides and imaged with an Olympus VS120 whole slide-scanning microscope with a UPLSAPO 40x/0.95 objective lens.

#### Non-Invasive Gap Junction Tracing.

To achieve sparse-but-strong overexpression of the serotonin transporter, a cocktail of AAV-BI30:CAG-FLPo-miR122-WPRE and AAV-BI30:CAG-fDIO(SERT-HA)-miR122-WPRE were intravenously co-administered to adult mice via tail vein injection. Viruses for these experiments were either generated in-house as previously described ^22,94^ or produced by Janelia’s Viral Tools core facility. It is widely acknowledged that AAV titers measured across different laboratories or production are not directly comparable due to non-standardized methodology ^95^. In line with this challenge, we observed that Janelia’s titering workflow measured viral preparations approximately one log-fold more concentrated than in-house measurements and consequently applied a correction during vial dosing (i.e. we dosed with 1x10^12^ vg as measured by Janelia to achieve transduction comparable to experiments with viruses produced in-house at a 1x10^11^ vg dose).

We empirically determined that a 1:1 – 10:1 ratio of FLP-dependent SERT to FLPo produced levels of expression suitable for the assay, with a total combined dose of 1.1 – 6 x 10^11^ vg / animal (after correction). This result was somewhat surprising, given that similar doses of our FLPo construct administered in isolation produced high levels of recombination in the FLP-dependent Ai65F reporter (Figure S1D). We speculate that this effect results from competition between the co-administered constructs or comparatively refractory recombination of the fDIO switch governing SERT expression. Perfusion-loading was performed >6 weeks after viral injection to allow time for recombination and steady-state overexpression. The assay was performed up to 18 weeks post-administration with qualitatively indistinguishable results – consistent with previous reports of stable, long-term transgene expression in CNS endothelial cells following systemic transduction with AAV vectors ^22,96^.

To achieve cell loading, a DMEM-based perfusate containing heparin (20U/mL; Sigma H4784) and 1–100µM serotonin (Cayman Chemical 14332) was freshly prepared and pre-warmed to 37°C. Mice were deeply anesthetized with intraperitoneal injection of a ketamine / xylazine solution and transcardially perfused with this solution for ~19 minutes at a flow rate of ~9–10mL /min. Next, PBS was delivered for ~1 minute to wash out excess serotonin perfusate followed by ~2–4 minutes of ice-cold 4% PFA to achieve fixation. Immunostaining was subsequently performed as described above.

We chose a perfusion-based loading approach because it allowed for the simultaneous loading of numerous endothelial cells within the intact vasculature. However, this strategy introduced caveats inherent to the technique. If perfusion quality was poor in a particular watershed, we observed poor loading of SERT-expressing cells and retention of luminal cells that stained brightly for serotonin (likely platelets ^97,98^). Alternatively, if washout was incomplete, we observed a low-level background serotonin signal throughout the vasculature. With good perfusion, true loading events were readily discernible from these artifacts. We observed substantially higher signal-to-noise detection of gap junction coupling events using a 1µM perfusate in brain.

Notably, the original report from *Hou et al.* describing a serotonin-based gap junction tracing assay speculated that SERT-expressing cells exposed to the circulation (i.e., endothelial cells) would passively accumulate serotonin, limiting temporal control over tracer introduction *in vivo*. This notion – at odds with the perfusion-loading approach utilized we used in this study – was based on the authors’ claim that serotonin is present at micromolar concentrations in blood plasma (0.5–17µM) ^20^. In fact, platelets in the bloodstream robustly express the serotonin transporter ^98^ and actively sequester blood-borne serotonin ^97^. As a result, while serotonin is abundant in whole blood, concentrations in plasma only reach ~2nM ^99^.

#### Quantification of SERT-Based Gap Junction Tracing.

Retinal vascular whole-mounts selected for quantification were exhaustively examined for SERT^+^ probe cells expressing high levels of the transporter. Confocal z-stacks of the local vasculature within a given two-dimensional vascular plexus were acquired for analysis. Serotonin intensity was highly variable across cells, an observation likely explained by variation in the strength of transporter expression, quality of perfusion-based cell loading, or extent of intercellular diffusion to neighboring cells. As a result, laser power used for serotonin acquisition was modulated on a per field-of-view basis to avoid oversaturation and optimize dynamic range. Cells identified in regions of the vasculature (i) damaged during sample preparation, (ii) exhibiting minimal serotonin tracer-loading, or (iii) spanning multiple vascular plexuses were excluded from analysis.

Isolectin was used to define the bounds of the vasculature. String vessels, discontiguous vasculature captured in the z-projection, and artifactual debris captured in this channel were manually removed from images. Cell-cell tracer transfer was then quantified using a custom Python script to calculate a ‘coupling index’ metric, defined as the background-corrected intensity ratio of serotonin signal within a given SERT^+^ probe cell / serotonin signal within the most proximal 𝑎 × 3 pixels in a mask of contiguous vasculature, where 𝑎 was set as area of the probe cell. Of note, the boundaries of SERT^+^ probe cells or vasculature were occasionally annotated manually if automatic segmentation failed; this issue was particularly pronounced in the deep plexus due to punctate non-specific HA reactivity in the parenchyma of this layer.

Arterial, capillary, and venous endothelial cells were distinguished using morphological criteria. Vessels > 7µm in diameter (estimated manually) were identified as arteries or veins based on stereotyped appearance and position in the retinal vasculature. Vessels < 5.5µm in diameter were identified as capillaries. Vessels 5.5–7µm in diameter were classified as follows: (i) all such cases in the intermediate plexus were identified as capillaries and (ii) in the superficial or deep plexuses, segments immediately proximal (<60µm traced along the vasculature) to a clearly defined artery or vein were classified as arterial or venous, respectively. Segments further from large vessels were classified as capillaries.

#### Serotonin Antisera Clearing Workflow.

Because the serotonin molecule is too small to elicit an immunogenic response alone, it has conventionally been coupled to bovine serum albumin (BSA) with formaldehyde for immunization ^100^. In order to improve signal-to-noise on serotonin detection, we developed a pre-clearing workflow to remove non-specific reactivity. One milliliter of rabbit anti-serotonin whole antisera (Sigma, S5545) – raised against a serotonin ◦ BSA complex, as described above – was diluted 1:10 in PBS and supplemented with BSA to a final concentration of 15 mg/mL.

To generate a substrate for negative purification, HEK-293 cells were grown to confluency in PLL-coated 10cm^2^ dishes in DMEM supplemented with small-molecule (< 10kDa) dialyzed Fetal Bovine Serum (Gibco, 26400044). HEK-293 cells do not express Aromatic L-amino Acid Decarboxylase (DDC) ^101^, an essential enzyme for serotonin synthesis ^102^. Therefore, cell culture in media made up with small-molecule dialyzed FBS was assumed to be nearly serotonin-free. Plates were fixed in 4% PFA for 30 minutes at room temperature, washed 3x in PBS, permeabilized for 15 minutes in 0.1% PBST, and stored in PBS at 4°C until use.

The diluted serotonin antisera was then added to a HEK-coated plate and incubated for ~24 hours at 4°C with agitation to sequester non-specific elements. This process was repeated for 10 consecutive days, followed by centrifugation at 17,000 x g for 30 minutes at 4°C to eliminate cellular debris. The partly cleared antisera was next passed over a BSA ◦ agarose affinity column (Alpha Diagnostic, BSA15-AS) to remove BSA-reactive antibodies. Finally, the antisera was passed over HEK-coated plates for an additional 10 days, centrifugated, stored at −20°C, and used at 1:100 in downstream immunostaining applications.

#### Aortic Endothelial Cell Electrophysiology & Neurobiotin Tracer Experiments.

Mice were anesthetized with isoflurane and euthanized. The thoracic aorta was acutely isolated and transferred to external solution used for electrophysiology containing (in mM): 134 NaCl, 6 KCl, 1 MgCl2, 2 CaCl2, 10 Glucose, 10 Hepes, with pH adjusted to 7.3 using NaOH as described in ^103^. Periaortic fat and connective tissue were removed via fine dissection, and each aorta was divided into four segments. Immediately before use, a segment was opened *en face* and held flat in a recording chamber using a U-shaped platinum frame strung with nylon fiber.

Patch electrodes (11–13MΩ after filling with solution) were pulled from borosilicate capillaries (BF150-86-10, Sutter Instrument) and filled with internal solution containing (in mM): 110 KCl, 13 NaCl, 2 MgCl2, 1 CaCl2, 10 HEPES, 10 EGTA, with pH adjusted to 7.3 using KOH. 20µM Alexa Fluor 568 Hydrazide (Invitrogen A10437) was dialyzed through the recording pipette to reveal cellular morphology and confirm cell type. A 1% m/v ratio of Neurobiotin (Vector Laboratories SP-1120) was included in the internal solution for gap-junction tracing. Final osmolarity of the internal solution was ~300mOsm/L. Whole-cell patch-clamp recordings were made at room temperature with an Axon Instruments Axopatch 200B amplifier. The series resistance of patch electrodes was 30–50MΩ. Endothelial cells were voltage-clamped at −40mV ^14^. A 400ms +10mV voltage step to −30mV was applied to measure membrane input resistance and capacitance, whose values were calculated using Clampfit software.

After a 10-minute cell-fill to allow for intercellular diffusion of Neurobiotin, aortic segments were fixed in 4% PFA for 30 minutes at room temperature. The tissue was immunostained with 1:100 Goat anti-CD31 (R&D Systems AF3628) and 1:500 Streptavidin◦AF647 (Thermo Fisher S32357) and mounted *en face* as described above. A Leica TCS SP8 confocal microscope was used to tile-scan the full extent of Neurobiotin spread, and tracer-positive endothelial cells (CD31^+^ cells with perinuclear accumulation of tracer) were manually quantified using (Fiji is Just) ImageJ’s Cell Counter plugin.

#### Cranial Window Implantation.

The cranial window implantation workflow was based on the protocol described by *Goldey et al*. (ref. ^104^)The craniotomy was centered over visual cortex at approximately 3.1mm posterior and 2.8mm lateral to bregma. The perimeter of the craniotomy was traced using a 4mm circular biopsy punch (VWR 21909–140) marked with a surgical marker (Aspen Surgical 1000-00-PDG). Next, a micro-motor drill (Foredom MH-170) outfitted with a 0.45mm carbide burr bit (Stoelting 514551) was used to carefully remove the bone along the circumference of the craniotomy trace. The resulting circular bone flap was subsequently removed with fine forceps while continuously irrigating with saline to avoid damage to the pia vasculature. A cranial window composed of three pieces of round cover glass (two 4mm, one 5mm; Warner Instruments CS-4, 64–0724 & CS-5R, 64–0700) glued together with a UV-curable adhesive (Norland Products NOA68) was carefully lowered onto the exposed brain and bonded to surrounding regions of the skull with dental cement (C&B Metabond; Parkell S396, S398, S371). Finally, a custom-made titanium headplate and circular well were centered on this window and attached to the skull with dental cement to enable head-fixation and facilitate use of a water-immersion objective.

#### Visual Stimulus Presentation.

Visual presentation was performed using Psychophysics Toolbox Version 3 and a 17” LCD monitor (Dell E1715S E Series) placed 15cm from the mouse’s left eye. Monitor position relative to the mouse was identical across widefield and two-photon imaging modalities. All visual stimuli were 4 seconds long with a 29 second inter-trial interval (ITI). In experiments where different stimuli were presented to mice, the order of presentation was randomized.

18° blinking-circle stimuli (Figure 4) consisted of gray screen with an ellipse (placed in one of four positions in a 2x2 grid) alternating between black and white with a frequency of 2Hz. Blank screen control trials maintained this gray background but omitted an ellipse. Stimuli were spherically corrected on the screen to maintain the appearance of a 9° radius circle from the perspective of the mouse’s left eye, regardless of their location in visual space ^30^. For experiments with a full-screen stimulus presentation (Figure 6) we used a bandpass filtered noise stimulus (with a maximum spatial frequency cut-off of 0.08 cycles per degree and a maximum temporal frequency cut-off of 3Hz; ref ^105^) to avoid large changes in luminance throughout the trial. As with the smaller blinking-circle stimuli, bandpass-filtered noise was binarized (i.e. black and white with no grays).

#### Widefield Imaging.

Imaging was performed similar to Bouchard et. al (ref ^106^). Briefly, synchronization between LED lights and camera acquisition were controlled by an Arduino Uno Rev 3 microcontroller. Blue (470nm; Thorlabs M470L4) and green (530nm; Thorlabs M530L4) LEDs were used to acquire GCaMP and HbT IOS signals, respectively. A 500nm long-pass filter (Edmund Optics 84–756) was placed in the collection path to block reflected blue light. LEDs began alternating 5 seconds prior to the start of each recording to mitigate neural activity triggered by the imaging light itself. After this 5-second interval, LED illumination was synchronized to triggering of camera chip exposure (Dalsa Nano #G5-GM30-M4040), alternating between GCaMP frames (90ms long, 50ms exposure) and IOS frames (50ms long, 10ms exposure) for a total time of 140ms for each set of images. No GCaMP was expressed in optogenetic stimulation experiments, so the blue LED was omitted; in these sessions the green LED was used alone at 55ms/frame (5ms exposure time). A custom tandem-lens macroscope system (Rokinon #135M-N and #85M-C lenses producing ~1.6x magnification) was used for imaging.

#### Stimulus-Evoked Neural Response Mapping.

To achieve pan-neuronal GCaMP overexpression throughout the brain, a 3.5x10^11^ vg dose of AAV-PHP.eB (ref. ^21^) packaged with pAAV-Syn-jGCaMP8m-WPRE (Addgene #162375, a gift from the GENIE Project ^85^) was intravenously delivered via tail vein injection prior to tamoxifen administration. Preparations of this virus were produced by the Boston Children’s Hospital Viral Core or Janelia’s Viral Tools core facility.

Mice were habituated to head-fixation for four consecutive days before collecting any form of imaging data. After habituation, signal from neuronally-expressed GCaMP was used to map stimulus-evoked cortical activity across three days of widefield imaging. Each session consisted of 15 repeats of each stimulus (including a blank-screen control), randomly interleaved. Evoked responses to small (18°) and large (full-screen) stimuli were recorded in separate sessions and considered independently during normalization. Each movie was motion-corrected and registered to a common coordinate system shared across all sessions. Next, GCaMP signals were hot pixel corrected, smoothed with a 90µm Gaussian kernel, and corrected to account for hemoglobin absorption at 470nm using the simultaneously acquired HbT signal ^31^. Each pixel’s value was then normalized to its mean value recorded immediately prior to visual stimulation across all trials in a given session. Finally, maximum values were normalized to the maximum response recorded in response to any stimulus across the entire session. Signal detected outside the cranial window was excluded from consideration during rescaling.

Next, the average GCaMP signal in a 1.12 second window following the onset of stimulus presentation was taken as the evoked GCaMP response for each trial. A total of 45 responses (15 trials x 3 sessions) were then averaged to map the pattern of cortical activation produced by each visual stimulus. A final normalization step was performed, scaling the maximum and minimum value of this image to the 99^th^ and 15^th^ percentiles of the signal observed across all pixels in all trials for a given mouse. To calculate the distance-to-activity metric, the image was binarized, setting all pixel values >0.5 as “activated” areas.

#### Two-Photon Microscopy.

Two-photon imaging was performed with a custom-built microscope using a Ti:sapphire laser (MaiTai HP DS, Spectra-Physics) tuned to 800nm. Laser intensity was modulated by a Pockels cell (Conoptics), and beam position was controlled by resonant (x-dimension) and galvo (y-dimension) scan mirrors. A 25X, 1.1NA objective (Nikon CFI75 Apochromat 25XC W) was used for functional measurements. ScanImage 2022 was used to control all microscope components. Immediately prior to imaging, 50–100µL of a FITC ◦ 2MDa Dextran solution (1.5mg/100µL; Sigma FD2000S) was intravenously injected via the tail vein to identify the vasculature. During each trial, pupil diameter was recorded using a camera (FLIR FL3-U3-13E4M-C) and locomotion was tracked using a rotary encoder (Sparkfun COM-10932) attached to the free-moving ball the mouse was mounted on. Imaging sessions lasted no longer than 90 minutes.

For arterial dilation measurements, measurements were acquired at each region-of-interest across three separate days of imaging. All imaging data were motion-corrected with rigid image registration and spatially smoothed using a 1µm Gaussian kernel. Centerlines of individual arteries were manually demarcated. Vessel width was then measured perpendicular to the centerline by fitting the edges of the vessel. Width was measured at regularly spaced intervals of 10 pixels along the centerline, yielding multiple dilation trajectories for a single vessel on a given trial. Trajectories with poor edge fitting (due to motion artifacts or out-of-focus imaging) were discarded. The remaining trajectories were averaged to produce a single trajectory per vessel, per trial. For diving vessels, which are oriented perpendicular to the image plane of the microscope, diameters were quantified via thresholding in radon space as described by *Gao & Drew* (ref ^107^).

Baseline arterial diameter and cerebral blood flow measurements (Figure S6C, D) were acquired in the absence of a visual stimulus presentation. To measure baseline arterial diameter, all arteries visible within the cranial window were sampled across a single imaging session and resting width was quantified as described above. To measure resting cerebral perfusion, capillaries were sampled throughout the cranial window at ~100µm below the pia surface. Measurements were taken in three sessions across separate days, with ~5–15 capillaries imaged per session. Across recordings, a total of 23.2 ± 1.2 (mean ± s.e.m.) measurements were obtained per animal. Capillary blood flow was quantified using an iterative Randon transform as described by *Chhatbar & Kara* (ref ^108^). Flow velocity for each trial was taken as the median velocity measured across 10 seconds of imaging.

#### Trajectory Filtering & Behavioral Correction.

During two-photon imaging, recordings at different physical locations in the arterial network were necessarily made asynchronously, meaning that stimulus-independent, state-related influences on vasomotion present on any given trial were not necessarily shared across measurements. Accordingly, to reduce variability arising from changes in diameter linked to internal state, we performed both trajectory filtering and behavioral corrections on two-photon functional imaging data:

##### Trajectory Filtering.

Two forms of filtering were performed. First, trajectories with high amounts of vasomotion prior to visual stimulus presentation were excluded. This was achieved by calculating a mean baseline diameter value for a given vessel during a given imaging session, defined as the average diameter prior to visual stimulation across all trials in that session. For each trial, vessel diameter in the pre-stimulus period was then Z-scored by subtracting this mean baseline value and dividing by its standard deviation. Trials where the 96^th^ percentile of values in the pre-stimulus period exceeded an absolute Z-score of 3 were excluded from analysis.

Second, trajectories reporting noise-level stimulus-evoked dilation were set to zero (i.e. ∆D/D = 0 across the entire the measurement). To do this, we calculated a spectrogram of each vasomotion trajectory and calculated the mean power in a band spanning 0.5 – 1.3 Hz, which corresponds to typical dilations. A threshold for true dilations was empirically set at 3 dB/Hz. Trajectories whose power measured less than this threshold in a 2.3 second period following stimulus onset were counted as zero dilation.

##### Behavioral Correction.

It is well-established that neural activity in visual cortex correlates with locomotion ^109,110^ and pupil diameter ^111^. Consequently, we accounted for their influence on vasodilation on a per-mouse basis. Both locomotion and pupil diameter were taken as the deviation of these values from the mean across all trials for a given animal. To estimate their effect, we employed a linear mixed-effect model using the MATLAB function fitlm as follows:

$$y_{i,j}=\beta_{00}+\beta_{10}l_{i,j}+\beta_{20}p_{i,j}+U_{0j}+\varepsilon_{i,j}$$

Where $\beta_{00}$ is the global intercept for the individual mouse; $U_{0j}$ is the mean response of a given vessel-stimulus combination, j; $l_{i,j}$ and $p_{i,j}$ are the total locomotion and maximum pupil diameter measured on trial i for vessel-stimulus combination j; $\beta_{10}$ and $\beta_{20}$ are the slope coefficients for locomotion and pupillometry terms; and $\varepsilon_{ij}$ is the residual. If the slope terms were not found to be statistically significant (p < 0.01) no correction was applied. However, if a significant slope term was found then the locomotion and/or pupil effects were regressed from the vasomotion measurements.

#### Quantification of Simultaneously Acquired GCaMP / IOS Measurements.

Movies of simultaneously acquired GCaMP / IOS signals recorded on the widefield were processed as follows. Background movies were captured with excitation LED power set to zero, but otherwise stimulus-identical to experimental trials. These values were subtracted pixelwise and framewise from each trial’s data. Following background subtraction, rigid body registration was performed to account for slight motion artifacts during imaging. This was followed by registration to a single reference image of the vasculature to account for minor rotations and/or translations of the window relative to the camera across sessions.

Centerlines of well-resolved arteries were then manually selected in each cranial window by an observer blinded to genotype. Segments that overlapped with other vessels (predominantly large veins), appeared out of focus during the imaging session, or occurred at tortuous collaterals were excluded. Vasomotion was then quantified for vessel segments spaced at 20µm intervals along these centerlines. Because hemoglobin (in both its oxygenated and deoxygenated forms) strongly absorbs 530nm light ^31^, areas with greater blood concentration appeared darker in IOS images acquired with this wavelength. Consequently, images were inverted such that higher values represented greater values of HbT.

For each segment, the immediate region encompassing the vessel was smoothed via convolution with a one-dimension Gaussian kernel (5.6µm in width) oriented parallel to the vessel’s course. This orientation avoided smearing the vessel’s edges but allowed for averaging along the vessel’s length. Next, the intensity profile of the image perpendicular to the vessel direction was taken and smoothed with a symlet to reduce pixel noise, while preserving the relatively sharp boundary between the pial artery and surrounding issue. The length of this profile was 70µm and was interpolated to be 200 points long. Intensity of parenchymal tissue immediately surrounding the vessel was taken as the average of the intensity minima on each side of the vessel and subtracted from the intensity profile to isolate an arterial signal. Finally, vessel width was determined as the full width at half maximum (FWHM) of the intensity profile, and HbT signal was quantified as the sum of the intensity profile across the FWHM.

Unlike our two-photon-based approach, synchronous GCaMP / IOS imaging provided a direct readout of neural activity on a trial-by-trial basis. As a result, rather than referencing measurements to an average stimulus-evoked pattern of neural activity, stimulus evoked responses could be computed for each individual trial as follows.

After image registration, a mask of the pial vasculature was estimated from IOS images using a Frangi vesselness filter. These masked pixels were excluded during downstream processing of the GCaMP signal. Additionally, a hemoglobin absorption correction was applied (as previously described). Following these steps, 4x4 pixel binning was applied to the adjusted GCaMP image and spatial smoothing was performed via convolution with a 100µm Gaussian kernel. Baseline signal for each pixel was determined as the average value measured across all trials in a given session prior to visual stimulus display. Subsequently, movies were temporally smoothed with a 5-frame moving average. Each pixel was then divided by its baseline value and normalized to a range spanning the 15^th^ to 99^th^ percentile of all GCaMP values measured during that imaging session. Stimulus-evoked activity maps could then be generated on a trial-by-trial basis by taking the average GCaMP signal in a 1.12 second window immediately following stimulus triggering on each trial and identifying all pixel values >0.5 as “activated” areas.

Because simultaneous GCaMP / IOS imaging allowed us to measure neural activity in real-time, we did not include proxies for internal state (locomotion and pupillometry) in the analysis as with two-photon microscopy. Instead, we directly quantified neural activation local to each arterial segment (i.e. $l\left(s,t\right)$ giving the GCaMP value local to vessel segment s at time t) and excluded observations associated with high levels of neural activity prior to visual stimulus presentation. This was done by first thresholding the GCaMP signal with a more conservative threshold than that used for calculating distance-to-activity (0.2 rather than 0.5) with the goal of capturing low-grade neural activity. Next, this map was convolved with a two-dimensional kernel to give:

$$l=e^{-\frac{D^{4}}{2\sigma^{4}}}$$

Where D is the distance (in microns) from the vessel segment and σ is a distance weighting parameter, which we set to 400µm. Observations of individual vessel segments where the average value of log{l(s, t)} measured > 6 in the pre-stimulus period on a given trial were omitted from analysis.

#### Optogenetic Stimulation.

For optogenetic stimulation experiments, cranial window implantation was shifted to somatosensory cortex and centered at approximately 1.8mm posterior and 2.65mm lateral to bregma. After drilling and removal of bone, a pulled glass needle (fabricated from a 1mm OD / 0.58 ID borosilicate capillary; Sutter Instrument BF100-58-10) was attached to a 5µL Model 75 RN Syringe (Hamilton 7634-01) using a 1mm RN Compression Fitting (Hamilton 55750-01). The apparatus was backfilled with mineral oil using a priming syringe (Hamilton PRMKIT), secured to a syringe pump (Harvard Apparatus 70-4507), and front-loaded with an AAV9 preparation of pAAV-Syn-ChRmine-mScarlet-Kv2.1-WPRE (Addgene #130995, a gift from Karl Deisseroth ^32^) produced by the Boston Children’s Hospital Viral Core. The glass needle was positioned adjacent of a branch of the middle cerebral artery and inserted slowly into the brain to a depth of 600µm. After allowing 5 minutes for equilibration, the needle was retreated to a depth of 250µm and ~100nL of AAV (~1x10^9^ viral genomes) was injected over the course of 10 minutes. The injection was followed by an additional 5-minute equilibration step before needle withdrawal, cranial window placement, and headplate attachment.

Animals were allowed to recover for 10 days before to being habituated to head-fixation in the four days prior to data acquisition. Next, imaging was performed on three consecutive days (14 – 16 post-AAV injection), with each session consisting of 30 trials at each stimulus power (0, 2.5, 10 mW/cm^2^) randomly interleaved. Optogenetic stimulation was achieved using a 530nm laser diode (Thorlabs L520P50). Power at the cranial window was measured using a digital power meter (Thorlabs PM100D). For both 2.5 and 10 mW/cm^2^ conditions, stimulation was performed at 9Hz with a 0.09 duty ratio. Stimuli were delivered for 2 seconds on each trial, with optogenetic pulses timed to occur during camera readout. Because optogenetic stimuli drove substantial dilations, we did not use a fixed ITI in order to avoid potential entrainment of vasomotor oscillations ^112^. Instead, each ITI was selected from a Gaussian distribution centered at 30 seconds with a width of 5 seconds and a set minimum of 20 seconds.

#### Derived Vasomotion Parameter Quantification.

For each experimental trial in our functional imaging experiments, one or more of the following parameters were derived from vasomotion trajectories: maximum dilation, time-to-peak, and dilation onset. Maximum dilation was measured as the maximum of the vasomotion parameter (either ∆D/D or ∆HbT/HbT) during the stimulus (visual) or a period encompassing the stimulus and the four seconds following (optogenetic). Time-to-peak was measured as the length of time after stimulus onset this maximum value is reached. Finally, dilation onset was measured by first averaging all trajectories obtained at each vessel position for a given stimulus. A sigmoid function was then fitted to this average trajectory, with the onset defined as the time at which the fit reached 10% of its maximum value. Across all functional experiments, only arteries > 15µm in diameter at baseline were analyzed.

#### Calculation of Propagation Velocity.

The velocity of arterial dilation propagation was calculated from dilation onset times recorded during optogenetic stimulation experiments. ΔHbT/HbT was recorded at 45µm intervals along a pial artery emanating outwards from the center of cortical ChRmine transduction (i.e. Figure 5D). Dilation trajectories at each location were then averaged across 30 technical replicates (3 imaging sessions of 10 repeats each). The portion of this average trajectory from 1.5 seconds prior to stimulus onset to 0.5 seconds after maximum dilation was reached was next fit to a sigmoid function as follows:

$$\frac{\Delta HbT\left(s,t\right)}{HbT}=a+\frac{b}{1+e^{\frac{c-t}{d}}}$$

Where t is time and s is the position along a sampled artery relative to the area of ChRmine transduction. The equation was then solved for the time at which the function reached 10% of its maximum value at each position. This gave onset time as a function of distance, a relationship that was well-fit with a straight line (Figure 5E). The inverse of the slope of this line was taken as the propagation velocity for a given mouse.

#### Linear Mixed-Effect Modeling.

Throughout the study, functional data was acquired on a per-field of view (two-photon) or per-trial basis (widefield). These measurements were further nested within individual mice of a given genotype. Moreover, as there are multiple influences on arterial diameter in awake mice aside from those experimentally controlled ^113^, random effects should be accounted for. Consequently, to analyze our data we opted for linear mixed-effect modeling ^114^ using a hierarchical random effects analysis of variance model ^115^ with the MATLAB function fitlme.

This model made no assumptions about the functional form of how distance from neural activity influences vasomotion. Instead, data were first sorted into discrete distance-from-activity bins. Initially, mice of each genotype were modeled separately. The vasomotion for each bin (e.g. max ΔD/D) was modeled as the intercept with a random effects terms encompassing trial-to-trial variability, which was nested within each mouse as follows:

$$y_{i,j,k,m}=\beta_{00}+{\text{U}}_{0ijk}+\varepsilon_{i,j,k,m}$$

Where y is the measured vasomotion parameter on trial m of vessel k, nested in field of view j, nested in mouse i; $\beta_{00}$ is the intercept; $U_{0ijk}$ is the random effect term; and ε is the remaining residual for the observation. For widefield measurements, because all vessels were measured simultaneously, the random effect term was structured for a given trial, nested within a given mouse.

Next, to estimate differences between the genotypes, all mice were pooled and collectively modeled as above - but now including a dummy variable encoding the genotype to scale the intercept. To account for the influence of differences in baseline diameter (Figure S6G), an additional slope term was included for vessel diameter (measured as deviation from the mean vessel diameter across all mice).

#### Blood Pressure Recordings.

Systolic and diastolic blood pressure were measured in awake mice via volume-pressure recordings (VPR) from a tail-cuff ^116^ using the CODA8 Non-Invasive Blood Pressure System (Kent Scientific Corporation). To obtain measurements, mice were acclimatized to a quiet area for ≥ 30 minutes before being gently encouraged to walk into a tube placed atop a heating pad. Restraints were then positioned within the tube to prevent excessive movement during recordings, and occlusion / VPR cuffs were placed on the tail. The animal was next warmed ~10 minutes until the temperature at the base of the tail measured 32 – 35°C using a non-contact infrared thermometer. This temperature range was maintained for the duration of the recording. Mice were habituated to restraint, heating, and tail-cuff inflation for three days prior to data acquisition. Subsequent recording sessions consisted of 5 ‘acclimation trials’ excluded from downstream analysis followed by experimental trials. Trials with motion artifacts or a tail volume deflection of < 15 µL were discarded. A minimum of 5 accepted trials (mean of 14.9) were obtained within a recorded session. CODA Data Acquisition Software Version 4.1.0.0 (Kent Scientific Corporation) was used to calculate systolic and diastolic values from pressure traces. All recordings were performed between 12 – 4:30PM (12:12 light-dark cycle; ZT 5 – 9:30) in randomized order to minimize circadian effects ^117,118^. Four days of recordings were obtained per animal and averaged on a per-day basis to obtain a final measurement. Experimenters were blinded to genotype during data acquisition and processing steps.

#### Post-Hoc Arterial Network Registration.

Brains were isolated from mice utilized for live imaging after transcardial perfusion, cut into halves along the sagittal midline, and post-fixed in 4% PFA on ice for 30 minutes. The cortical region captured beneath each animal’s cranial window was visually identified and precisely isolated using a razor blade. Subcortical structures were carefully removed from the underside of the preparation, and the sample was subsequently post-fixed for an additional 15–30 minutes in 4% PFA on ice. Immunostaining was then carried out as described above for pia vasculature whole mounts. A custom-made spacer (a 24x40mm rectangle with a 2mm wall width, forming a 20x36 well) was laser-cut from 1/16” acrylic (McMaster Carr 8560K172) and adhered to a glass slide using nail polish. The slide-well was filled with ~1.5mL of PBS and the sample was added dorsal-side up. Finally, a 24x40mm coverslip was gently pressed against the cortex to flatten the preparation and sealed in place using nail polish.

The cortical vasculature was next tile-scanned using a Leica TCS SP8 confocal microscope. The composite histology was then registered to an image of the live vasculature acquired on the widefield imaging setup via non-rigid transformation. Specifically, MATLAB’s implementation of a local weighted mean transformation (fitgeotrans) was employed using 20–25 arterial vertices as control point pairs. The resulting image was next manually thresholded to isolate the SMA^+^ arterial network and iteratively smoothed using (Fiji is Just) ImageJ’s Otsu Threshold and 3D Gaussian Blur functions. Finally, if necessary, instances of damage in the binarized image were manually resolved using the widefield image as a guide to generate a complete map of the network.

For optogenetic experiments, an image of mScarlet signal – acquired and transformed alongside the SMA^+^ arterial network – was binarized using CellProfiler 4.2.4 (ref. ^87^) to identify ChRmine^+^ areas of cortex. Two binarization strategies were used to generate overlay images included in the figures: a strict threshold (Otsu) was used to identify the centermost transduced region, and a permissive threshold (Robust Background) was used to identify the full extent of neuronal processes. The latter, more permissive region was used to calculate distances from ChRmine^+^ neurons associated with functional imaging measurements. An example of the entire workflow is illustrated in Figure S7I–J.

#### Image Processing and Data Analysis.

(Fiji is Just) ImageJ 2.0.0-rc-69 and Adobe Illustrator 24.2 were used to process images displayed in figures throughout the manuscript. GraphPad Prism v.9.1.1 and MATLAB (version 2023b) were used for statistical analysis and graph generation. BioRender was used to generate the schematic shown in Figure 4A.

##### Quantification and Statistical Analysis

No statistical method was used to predetermine sample size. Sample size was instead designed to approximately match similar studies of neurovascular coupling ^8,17,19^. Investigators were blinded to genotype during data acquisition in all functional imaging experiments. Animals exhibiting limited cranial window visibility, aberrant viral transduction, or apparent damage to pial arteries during stereotaxic injection were excluded from analysis prior to unblinding. Barring these exceptions, all attempts at replication were successful. See figure legends for the statistical test, definition of center & precision, sample size (*n*), and biological definition of *n* (i.e. cells, mice) associated with each experiment. Exact statistical test results for Figure 4E & H, Figure 5B, Figure 6C, and Figure S6G are reported in Supplementary Table 1. All other statistical test results and definitions of significance are specified in the figure legends.

## Resource Availability

### Lead Contact

Correspondence and material requests should be addressed to the lead contact, Chenghua Gu (chenghua_gu@hms.harvard.edu).

### Materials Availability

The pAAV-CAG-fDIO(SERT-HA)-miR122-WPRE-pA and pAAV-CAG-FLPo-miR122-WPRE-pA plasmids generated in this study have been deposited in the Addgene repository under identification numbers 220937 and 220938, respectively.

The *Gja4* ^*Flox*^ and *Gja4* ^*LacZ*^ strains generated in this study have been deposited to the Jackson Laboratory under stock numbers 039605 and 039606, respectively. In addition, the *Gja5* ^*Flox*^ strain originally described by *Chadjichristos et al.* (ref. ^75^) has been deposited under stock number 039730 with permission from Drs. van Veen, Kwak, and Chanson.

### Data and Code Availability

- All data reported in this paper will be shared by the lead contact upon request.
- All original code generated for this study has been deposited at Harvard Dataverse and is publicly available as of the date of publication (https://doi.org/10.7910/DVN/XRRGRI).
- Any additional information required to reanalyze the data reported in this paper is available from the lead contact upon request.

## Supplementary Material

1 **Supplementary Movie 1**. Vasodilatory responses to focal visual stimuli in aEC Cx dKO mutants and controls. (Related to Figure 4.)

2 **Supplementary Movie 2**. Vasodilatory responses to optogenetic stimuli of varying strength in control mice. (Related to Figure S7.)

3 **Supplementary Movie 3**. Vasodilatory responses to optogenetic stimuli in aEC Cx dKO mutants and controls. (Related to Figure 5.)

4 **Supplementary Movie 4**. Vasodilatory responses to full-screen visual stimuli in aEC Cx dKO mutants and controls. (Related to Figure 6.)

5 **Supplementary Table 1**. Exact statistical test results for live imaging experiments. (Related to Figure 4E & H, Figure 5B, Figure 6C, and Figure S6G.)

6 **Supplementary Table 2**. Genotyping primers and expected band sizes for transgenic mice. (Related to the STAR Methods.)

7 Figure S1. Additional characterization of CNS endothelial gap junction coupling via non-invasive SERT-based tracing. (Related to Figure 1).
**(A)** A cocktail of AAV-BI30 capsids packaged with FLPo or FLP-dependent SERT was intravenously administered to adult mice to achieve sparse-but-strong expression of the transporter in the retinal vasculature. After allowing >6 weeks for overexpression, animals were perfused with a serotonin-containing solution to achieve cell loading. The positions of SERT-HA expressing probe cells are demarcated with red arrowheads. Note attenuation of gap junction coupling strength with progression through the arterio-venous axis. Images are representative of results acquired from *n* = 5 animals.
**(B)** High-magnification image of serotonin spread from a SERT-HA^+^ arterial endothelial cell in retina. Note conspicuous labeling of abluminal-facing mural cell (red arrowhead), indicative of gap junction coupling between endothelial and mural layers of the vessel wall.
**(C)** For quantification of variation in cell-cell coupling strength along the arterio-venous axis, SERT^+^ probe endothelial cells from *n* = 4 retina whole mounts isolated from *n* = 3 mice were exhaustively examined. The coupling index presented was defined as the background-corrected intensity ratio of serotonin signal within a given probe cell / serotonin signal within the most proximal 𝑎 × 3 pixels in a mask of contiguous vasculature, where 𝑎 was set as area of the probe cell. A total of *n* = 10 arterial, 40 capillary, and 14 venous endothelial cells were included in the analysis. Mean ± s.e.m.; one-way ANOVA with Tukey’s multiple comparisons test (** p < 0.01, *** p < 0.001, **** p < 0.0001). Adjusted p values are as follows: arterial vs. capillary, 0.0002; arterial vs. venous, < 0.0001; capillary vs. venous, 0.0092.
**(D)** AAV-BI30:CAG-FLPo-miR122-WPRE was intravenously administered to adult Ai65F FLP-dependent reporter mice (*Rosa26:CAG-FRT-Stop-FRT-tdTomato*) and recombination was assessed after 14–16 days. Robust, endothelial-specific recombination was observed in the retina vasculature, validating efficacy of the FLPo construct. Similar results were obtained across *n* = 3 animals using three independent viral preparations.
Scale bars are as follows: 50µm in (**A**); 10µm in (**B**); 100µm in tdTomato channel, 50µm in merge of (**D**).

8 Figure S2. Generation and validation of Cx37 conditional knockout and reporter alleles. (Related to Figure 2).
**(A)** Schematic of International Mouse Phenotyping Consortium (IMPC) ‘knockout-first’ *Gja4* conditional allele used to generate Cx37^Flox^ and Cx37^LacZ^ mouse lines. Mating with a germline FLP driver removed the *LacZ* reporter and *Neo* selection cassette, producing the conditional Cx37^flox^ allele. Cre-dependent recombination of the Cx37^flox^ allele removes the entire *Gja4* coding sequence. The 3’ loxP site is inserted within the 3’ UTR. Importantly, this does not affect the *Gja4* coding sequence. Mating with a germline Cre driver removed the *Neo* selection element and the entire *Gja4* coding sequence, producing the Cx37^LacZ^ allele. Because the LacZ reporter’s expression is controlled by the endogenous promoter and enhancer elements present within the *Gja4* locus, X-Gal staining and ß-galactosidase immunodetection provide high-fidelity readouts of Cx37 expression.
**(B)** PCR genotyping of Cx37^Flox^ and Cx37^LacZ^ alleles. Primer sequences are described in Supplementary Table 2.
**(C)** Single confocal z-planes of pial arteries in Cx37^fl/fl^ and Tie2:Cre Cx37^fl/fl^ mice demonstrate that Cx37 protein expression from the Cx37^Flox^ allele is overtly indistinguishable from the Cx37^WT^ allele and as expected, the Cx37^Flox^ allele is susceptible to efficient Cre-mediated recombination.
(**D, E**) Comparison of identically treated samples obtained from Cx37^LacZ^ reporter and wild-type mice following enzymatic (**D**) or immunohistochemical (**E**) detection. Enzymatic data from Cx37^LacZ^ brain and retina same as shown in Figure 2D and G, respectively. Immunohistochemical detection of ß-Gal in Cx37^LacZ^ aorta and lung same as shown in Figure 3B and S3H–I, respectively.
(**F**) Control immunostaining conditions indicate that the co-localization of ERG and ß-Gal signals observed in lung capillaries is not an artifact of cross-reactivity between their respective primary antibodies, which were both raised in rabbit hosts.
Scale bars are as follows: 10µm in (**C**); 50µm in brain & lung, 5µm in aorta panel of (**E**); 50µm in (**F**). All images are representative of *n* = 3 animals per genotype except data shown in (**F**), which was acquired using serial cryosections obtained from a single Cx37^LacZ^ sample.

9 Figure S3. Expression of connexin isoforms across the cerebrovasculature. (Related to Figure 2).
(**A, B**) Published single-cell transcriptomics of the cerebrovasculature corroborate patterns of connexin expression observed with reporter lines. (**A**) The dataset acquired by *Jeong et al.* ^27^ (https://single-cell.mpi-muenster.mpg.de, UMAP seed #42) was used to analyze connexin expression in endothelial cells. Left: to orient the dataset along the arterio-venous axis, arterial-(*Bmx* ^68^ and *Gkn3* ^23^), capillary- (*Mfsd2a* ^19,23^), and venous-specific (*Ackr1* ^69^ and *Gm5127* ^70^) markers are shown. Right: expression of connexin isoforms Cx40 (*Gja5*), Cx37 (*Gja4*), Cx43 (*Gja1*), and Cx45 (*Gjc1*) reveals a stepwise pattern of arterio-venous zonation. (**B**) Data reported by *Vanlandewijck et al.* ^23^ (https://betsholtzlab.org/VascularSingleCells/database.html) shows similar patterns of connexin expression in endothelial cells (blue underline) and further reveals expression of these isoforms in other cell types of the neurovascular unit: mural cells (pericytes, aSMCs, and vSMCs; green underline) and astrocytes (orange underline). Cx43 (*Gja1*) was robustly expressed in astrocytes and both Cx37 (*Gja4*) and Cx45 (*Gjc1*) were expressed in mural cells. None of the other 16 connexin isoforms expressed in the mouse genome (*Gja3*, *Gja6*, *Gja8*, *Gja10*, *Gjb1*, *Gjb2*, *Gjb3*, *Gjb4*, *Gjb5*, *Gjb6*, *Gjc2*, *Gjc3*, *Gjd2*, *Gjd3*, *Gjd4*, *Gje1*) were detectable in endothelial or mural cells at levels above noise in either dataset. Abbreviations shown in (**B**) are as follows: PC – pericytes (red); SMC – smooth muscle cells (green); MG – microglia (grey); FB – vascular fibroblast-like cells (pink); OL – oligodendrocytes (tan); EC – endothelial cells (blue); ac – Astrocytes (orange); v – venous; capil – capillary; a – arterial; aa – arteriolar.
(**C, D**) Low- (**C**) and high- (**D**) magnification images of constitutive Cx37 (*Gja4*^*LacZ/+*^) and Cx45 (*Actb:Cre Gjc1*^*fl-KI-GFP/+*^) reporter expression in the brain microvasculature. Strong expression of both isoforms was invariantly observed in mural cells across the arterio-venous axis (i.e. smooth muscle cells in arteries and veins, pericytes in capillaries), contrasting the stepwise zonation seen in endothelial cells. Pericyte from Cx37^LacZ^ reporter shown in (**B**) same as displayed in Figure 2D.
(**E**) Constitutive Cx45 reporter expression in the pia vasculature confirms that this pattern is generalizable to the brain’s superficial arteries and veins.
(**F**) Constitutive Cx43 (*Actb:Cre Gja1*^*fl-KI-CFP/+*^) reporter expression in the cortex demonstrates robust expression in astrocytes, as previously shown ^25^. Note that strong fluorescence signals from mural cells and astrocytes obscure endothelial zonation of Cx43 and Cx45 evident in reporter crosses with the endothelial-specific *Tie2:Cre* driver.
(**G**) Schematic depicts organization of connexin expression throughout the neurovascular unit.
(**H**) Widespread expression of ß-Galactosidase is observed in the lung microvasculature of Cx37^LacZ/+^ reporter animals, consistent with reporters of robust Cx37 enrichment in this organ ^23,71,72^.
(**I**) High-magnification images of lung arterioles and capillaries reveal robust Cx37 expression in both endothelial subpopulations, contrasting arterial-specific zonation observed in CNS vasculature.
Scale bars are as follows: 50µm in (**C**); 10µm in (**D**); 75µm in (**E**); 50µm in the center and 20µm in the rightmost column of (**F**); 25µm in (**H**); 5µm in (**I**). Images shown are representative of *n* = 3 animals per genotype.

10 Figure S4. Cx37 and Cx40 are robustly expressed at arterial endothelial cell junctions, while reports of Cx43 expression in arterial endothelium likely stem from antibody cross- reactivity. (Related to Figure 3).
(**A**) Whole-mount immunostaining of the pia vasculature reveals robust expression of Cx37 and Cx40 at arterial (SMA^+^) but not venous (SMA^−^) endothelial-endothelial junctions. The signal associated with each connexin isoform was abolished in its respective knockout line, verifying specificity of the antibody reagents. Cx37 expression was strikingly diminished in Cx40 knockout animals.
(**B**) A reduced Cx37 signal was still apparent in individual z-planes of Cx40 knockout arteries acquired at higher laser power. Three observations – (i) complete loss of Cx37 signal in Cx37 knockout animals, (ii) complete loss of Cx40 signal in Cx40 knockout animals, and (iii) persistence of Cx40 signal in Cx37 knockout animals – collectively indicate that Cx37 downregulation is not an artifact of antibody cross-reactivity between the related isoforms.
(**C**) Schematic depicting coordinated regulation of arterial connexins. Cx37 appears to require the presence of Cx40 for its full expression in cerebrovascular arteries, a result consistent with prior reports in the peripheral vasculature ^29,45,73–75^. Previous studies report that arterial endothelial cells in the aorta ^76^, periphery ^43^, and brain ^77^ robustly express Cx43. These results contrast zonation we observe using an endothelial-specific Cx43 reporter (*Tie2:Cre Gja1* ^*flox-KI-CFP*^) where expression is specifically excluded from large arterial segments of the vasculature. This discrepancy is most likely explained by cross-reactivity of the antisera used in the aforementioned studies, as demonstrated by the following data.
(**D**) *En face* preparation of aortic endothelium isolated from adult Tie2:Cre Cx43^fl/fl^ mice and Cre- negative controls. Immunostaining with Sigma Anti-Cx43 (C6219) produces an indistinguishable signal at endothelial-endothelial junctions in both genotypes.
(**E**)PCR amplification of a 686 bp *Gja1* deletion-specific product from tail biopsies of Tie2:Cre Cx43^fl/fl^ animals using previously described primers ^78^, confirming Cre-mediated recombination.
(**F**) Aortic endothelium isolated from adult *Cx40*^*+/+*^ and *Cx40*^*-/-*^ mice. Junctional signal detected with Sigma Anti-Cx43 (C6219) is completely abolished in the Cx40 knockout, suggesting cross- reactivity of the antibody *in vivo*.
(**G**) Heterologous overexpression of a mouse Cx40◦GFP C-terminus fusion protein in HEK293 cells. Robust co-localization of Cx40◦GFP and Sigma Anti-Cx43 (C6219) signal at cell-cell interfaces demonstrates cross-reactivity of the antibody with the closely related Cx40 isoform.
(**H**)Amino acid sequence alignment of the peptide antigen used to generate the Sigma Anti-Cx43 (C6219) antibody and the C-terminus of the mouse Cx40 protein reveals substantial homology. Identical amino acids in the alignment are highlighted in red.
Scale bars are as follows: 50µm in (**A**); 5µm in (**B**); 20µm in (**D**) and (**F**); 5µm in (**G**). All images shown are representative of *n* = 3 animals per genotype or *n* = 3 experimental replicates.

11 Figure S5. Non-invasive gap junction tracing reveals loss of arterial endothelial-endothelial gap junction coupling in the CNS vasculature following inducible deletion of Cx37 and Cx40. (Related to Figure 3).
**(A)** Validation of the *BMX:Cre*^*ERT*2^ driver used for conditional loss-of-function studies. Adult *BMX:Cre*^*ERT*2^ *SUN1◦GFP* reporter animals (*Rosa26:CAG-LSL-Sun1◦GFP)* received 1mg / day tamoxifen for five consecutive days. Tissues were harvested two weeks after the final dose to assess recombinase activity, indicated by overexpression of an inner nuclear membrane-localized GFP. Efficient, predominantly arterial endothelial cell-specific recombination was observed in brain, retina, and aorta. Images are representative of *n* = 3 animals.
(**B**) A cocktail of AAV-BI30 capsids packaged with FLPo or FLP-dependent SERT was intravenously administered to adult *BMX:Cre*^*ERT2*^ *Gja4*^*fl/fl*^ *Gja5*^*fl/fl*^ and *Gja4*^*fl/fl*^ *Gja5*^*fl/fl*^ controls. Animals were subsequently treated with tamoxifen, and non-invasive gap junction tracing was performed ~6 – 9 weeks following the final dose in *n* = 4 animals per genotype. The positions of SERT-HA expressing probe cells in the retinal vasculature are demarcated with red arrowheads. Uppermost examples for each genotype same as shown in Figure 3G. Note robust intercellular serotonin diffusion in the large arteries of control animals. By contrast, serotonin accumulation is restricted to individual arterial endothelial cells in connexin-knockout animals, indicative of a near-complete loss of gap junction coupling. Gap junction coupling reappears in the small arterioles and capillaries of these animals, such that the two genotypes phenotypically converge with progression along the arterio-venous axis. Compelling tracer-spreading events in the large arteries of control animals were rare and observed in *n* = 2/4 mice examined; strong cell-cell coupling at these sites required both high levels of SERT overexpression and efficient cell-loading during intracardiac perfusion to produce a resolvable serotonin gradient. Conversely, single-cell fills in connexin-knockouts were commonly observed, even in arterial endothelial cells expressing relatively low levels of SERT: these events were observed in *n* = 4/4 mutants examined and never seen in controls. Small arteriole and capillary cell fills were seen in all animals examined, irrespective of genotype.
Scale bars are as follows: 200µm in low-magnification image of pia vasculature, 10µm in high- magnification image of pia artery, 75µm in retina, 25µm in aorta for (**A**); 50µm in (**B**).

12 Figure S6. Additional data and analyses relevant to long-range vasodilation propagation deficits observed in endothelial gap junction coupling mutants. (Related to Figure 4).
**(A)** Left: Example single-trial vasodilation responses evoked by presentation of 18° visual stimuli at sites proximal to activity (<1µm). Blue arrows indicate maximum dilation calculated from each trajectory. Duration of visual stimulation is demarcated in gray. Center: Example responses captured during blank screen control trials. Spontaneous vasomotion results in non-zero detected maximum dilations. Note that identification of maximum response is restricted to the 4-second window normally associated with stimulus presentation. Right: Averaging trajectories smooths out spontaneous vasomotion while preserving stimulus-evoked vasodilation (hierarchical bootstrap; mean ± 95% CI) because the former is not correlated with trial structure.
(**B**) Two-dimensional histograms of calculated maximum dilation versus time show clear time- locking to stimulus in visual-presentation but not blank screen trials. Data shown was acquired from tamoxifen-treated *Gja4*^*fl/fl*^ *Gja5*^*fl/fl*^ control animals via two-photon imaging (*n* = 9 mice, 344 trajectories for stimulus-presentation trials; *n* = 6 mice, 705 trajectories for blank screen control trials).
(**C**) Resting pial arterial diameter of tamoxifen-treated *BMX:Cre*^*ERT2*^ *Gja4*^*fl/fl*^ *Gja5*^*fl/fl*^ and *Gja4*^*fl/fl*^ *Gja5*^*fl/fl*^ controls measured *in vivo* via two-photon microscopy. Swarmplots show all measurements; each circle represents an artery, each color an individual mouse (*n* = 6 mutant / 4 control mice). Boxplots depict the same data, treating all vessels as independent observations. Midline is plotted at the median, with boxes extending to the 25^th^ and 75^th^ percentiles of the data. Outliers are plotted as ‘+’ symbols. Comparison with a Wilcoxon rank-sum test indicated a significant difference between the populations (p = 0.0235).
(**D**) Resting capillary red blood cell (RBC) velocity was similarly recorded in microvessels ~100µm below the pial surface. The median velocity measured in each animal and s.e.m. of these data are plotted (*n* = 6 mutant / 3 control mice). Comparison with a two-sample t-test indicated no significant difference between groups (p = 0.5040).
(**E**) Representative blood pressure recording obtained via volume-pressure recording (VPR) tail cuff sensor. Occlusion cuff, initially inflated to obstruct blood flow to the tail, is gradually relaxed across the trial duration. The occlusion pressure at first appearance of a pressure wave associated with blood re-entry is taken as the systolic blood pressure. The occlusion pressure at the inflection point of the curve – the point at which the rate of blood flow into the tail stops increasing – is taken as the diastolic blood pressure.
(**F**) Quantification of systemic blood pressure across genotypes. An average blood pressure of [153±4 / 129±4] versus [149±5 / 122±5] mmHg ([systolic / diastolic]; mean ± s.e.m.) was measured in control and connexin-knockout mice, respectively (*n* = 5 animals of each genotype). Comparisons performed with a two-sample t-test indicated no significant differences between groups (systolic: p = 0.4936; diastolic: p = 0.3943).
(**G**) To account for differences in resting diameter across genotypes – which could, in principle, blunt changes in diameter measured relative to baseline – a diameter term was added to analyses of dilation as a function of distance from activity (described in detail in the **Linear Mixed Effect Modeling** section of the **Methods**). Plots show the measured contribution of genotype to vasomotion in each imaging paradigm described in the study, with and without inclusion of this diameter term (mean ± 95% CI). Positive values indicate greater activity-evoked arterial dilation in controls relative to connexin-knockouts. Statistical test results associated with these analyses are reported in Supplementary Table 1.

13 Figure S7. Optogenetic stimulation enables precise and scalable control of neurovascular coupling, revealing aberrant vasodilation kinetics in endothelial gap junction-deficient mutants. (Related to Figure 5).
**(A)** Representative example of scalable responses in a control mouse (tamoxifen-treated *Gja4*^*fl/fl*^ *Gja5*^*fl/fl*^). Leftmost image shows arterial network in red and extent of ChRmine-expressing neurons in orange. Arrowhead indicates center of transduction. Maximum dilation responses at each location during the imaging window are shown for 0, 2.5, and 10 mW/cm^2^ conditions. Note long- range recruitment of arterial responses at highest stimulation power (also see Supplementary Movie 2).
(**B**) Average optogenetically-evoked dilation trajectories at the same three stimulation intensities, controlling for distance from ChRmine expression (*n* = 6 tamoxifen-treated *Gja4*^*fl/fl*^ *Gja5*^*fl/f*^ controls; 10mW/cm cm^2^ responses same as shown in Figure 6C).
(**C**) Left: representative sagittal brain section, demonstrating focal ChRmine overexpression in somatosensory cortex following stereotaxic injection. Right: a column of transduced neurons in the opsin-expressing region.
(**D)** Aggregated dilation responses of tamoxifen-treated *BMX:Cre*^*ERT2*^ *Gja4*^*fl/fl*^ *Gja5*^*fl/fl*^ mice and *Gja4*^*fl/fl*^ *Gja5*^*fl/f*^ controls at 0, 2.5, and 10mW/cm^2^ stimulation intensities (linear mixed-effect model; mean ± 95% CI; *n* = 6 animals of each genotype; 0 and 10mW/cm^2^ responses same as shown in Figure 5B,C). Control (0 mW/cm^2^) responses are pooled into a single datapoint, irrespective of position in arterial network.
(**E**)Average arterial dilation trajectories obtained from individual mice at locations within <1µm of ChRmine expression (by-genotype average trajectories are shown in Figure 5C). Data from each mouse is plotted as a separate trajectory. Note highly stereotyped differences in trajectory shape across genotypes.
(**F**)Comparison of time-to-peak dilation across genotypes, controlling for distance from ChRmine expression (linear mixed-effect model; mean ± 95% CI). Statistical test results are as follows: [<1µm; p < 0.0001], [1–400µm; p < 0.0001], [400–800µm; p < 0.0001], [800–1,200µm; p < 0.0001], [1,200–1,600µm; p = 0.0025], [1,600–2,000µm; p = 0.5588].
(**G**) Empirical cumulative distribution function (ECDF) plots of dilation onset for arterial segments ≤ 500µm from ChRmine^+^ neurons. Both individual animals (dotted lines) and by-genotype averages (solid lines) are shown. Note highly reproducible onset delay in aEC Cx dKO mutants.
(**H**)Average dilation onset time plotted as a function of distance from ChRmine expression for all arterial segments sampled in optogenetic experiments. High-power optogenetic stimulus (10 mW/cm^2^) was used for all data shown in (**E-H**); *n* = 6 animals of each genotype analyzed.
(**I**) Workflow for post-hoc arterial network registration. Left: a reference image of the cortical vasculature underlying the cranial window is acquired. After functional imaging is completed, the cortex is harvested, immunostained, and flat-mounted on a slide. A confocal microscope is subsequently used to tile-scan the entire cortical surface. Center: ~20–25 common arterial vertices are identified in the widefield and histology images. Right: These landmarks are used to transform the histological network into the *in vivo* coordinate space. The image is finally binarized and manually refined
(**J**) Identification of ChRmine-expressing regions of cortex. Left: a registered image of mScarlet signal – acquired and transformed alongside that of the arterial network – closely matches the relative position of fluorescence seen in the living animal. Center: this signal is binarized to define the spatial bounds of ChRmine expression. Right: images of the arterial network and viral transduction – identified in histology – are overlaid on a widefield capture of the living vasculature to produce a composite image. Pial arteries are now easily distinguished from pial veins and dural vessels visible in the window. Orange arrowhead depicts the center of viral transduction, defined as the center-of-mass of the mScarlet signal identified with a strict threshold.

14 **Supplementary Data 1**. Predicted maps of alleles generated in this study and sequence-validation of the Cx37^Flox^ CDS. (Related to the STAR Methods.)

## Figures and Tables

**Table:** Key Resource Table

REAGENT or RESOURCE	SOURCE	IDENTIFIER
**Antibodies**		
Rabbit anti-Connexin 37	Invitrogen	Cat: 40–4200 Lot: RC234271 RRID: AB_2533464
Rabbit anti-Connexin 40	Invitrogen	Cat: 36–5000 Lot: TE268368 RRID: AB_2533264
Mouse anti-Claudin 5	Invitrogen	Cat: 35–2500 Clone: 4C3C2 RRID: AB_2533200
Mouse anti-Claudin 5 ◦ AF488	Invitrogen	Cat: 352588 Clone: 4C3C2 RRID: AB_2532189
Mouse anti-αSMA ◦ FITC	Sigma	Cat: F3777 Clone: 1A4 RRID: AB_476977
Mouse anti-αSMA ◦ Cy3	Sigma	Cat: C6198 Clone: 1A4 RRID: AB_476856
Mouse anti-αSMA ◦ AF647	Santa Cruz	Cat: sc-32251 Clone: 1A4 RRID: AB_3661645
Rabbit anti-ßGalactosidase	MP Biomedicals	Cat: 085597-CF Lot: 08211 RRID: AB_3675281
Goat anti-CD31	R&D Systems	Cat: AF3628 Lot: Various RRID: AB_2161028
Rabbit anti-Cx43	Sigma	Cat: C6219 Lot: 0000122916 RRID: AB_476857
Rabbit anti-ERG	Abcam	Cat: ab92513 Clone: EPR3864 RRID: AB_2630401
Rabbit anti-ERG ◦ AF488	Abcam	Cat: ab196374 Clone: EPR3864 RRID: AB_2889273
Rabbit anti-GFP ◦ AF488	Invitrogen	Cat: A-21311 Lot: Various RRID: AB_221477
Rat anti-CD140b/PDGFRß	Invitrogen	Cat: 14–1402-82 Clone: APB5 RRID: AB_467493
Mouse anti-HA Tag ◦ AF555	Invitrogen	Cat: 26183-A555 Clone: 2–2.2.14 RRID: AB_2610625
Rabbit anti-Serotonin	Sigma	Cat: S5545 Lot: 0000152041 RRID: AB_477522
**Bacterial and virus strains**		
AAV-BI30: CAG-fDIO(SERT-HA)-miR122-WPRE-pA	This Study / Janelia Viral Tools	N/A
AAV-BI30: CAG-FLPo-miR122-WPRE-pA	This Study / Janelia Viral Tools	N/A
AAV-PHP.eB: pAAV-Syn-jGCaMP8m-WPRE	BCH Viral Core / Janelia Viral Tools	N/A
AAV9: pAAV-Syn-ChRmine-mScarlet-Kv2.1-WPRE	BCH Viral Core	N/A
**Biological samples**		
N / A		
**Chemicals, peptides, and recombinant proteins**		
Tamoxifen	Sigma	Cat: T5648
Heparin	Sigma	Cat: H4784
Serotonin	Cayman Chemical	Cat: 14332
Carbenoxolone	Cayman Chemical	Cat: 18240
Escitalopram	Tocris Bioscience	Cat: 4796
Small-Molecule (< 10kDa) Dialyzed Fetal Bovine Serum	Gibco	Cat: 26400044
Actin Red 555 Ready Probes Reagent	Invitrogen	Cat: R37112
Isolectin GS-IB_4_ ◦ AF488	Invitrogen	Cat: I21411
Isolectin GS-IB_4_ ◦ AF568	Invitrogen	Cat: I21412
Isolectin GS-IB_4_ ◦ AF647	Invitrogen	Cat: I32450
Neurobiotin	Vector Laboratories	Cat: SP-1120
Streptavidin ◦ AF647	Thermo Fisher	Cat: S32357
FITC ◦ 2MDa Dextran	Sigma	Cat: FD2000S
**Critical commercial assays**		
CODA8 Non-Invasive Blood Pressure System	Kent Scientific	N/A
Alexa Fluor 647 Antibody Labeling Kit	Thermo Scientific	Cat: A20186
**Deposited data**		
Custom Code for *In Vivo* Imaging Data Analysis, Post-Hoc Arterial Network Registration, and Quantification of SERT-Based Gap Junction Coupling	This Study, Harvard Dataverse	https://doi.org/10.7910/DVN/XRRGRI
**Experimental models: Cell lines**		
HEK293T	ATCC	Cat: CRL-3216
**Experimental models: Organisms/strains**		
C57BL/6NCrl	Charles River	Strain: 027
C57BL/6J	JAX	Strain: 000664
Cx37^Flox^	This Study / JAX	Strain: 039605
Cx37^LacZ^	This Study / JAX	Strain: 039606
Cx37^KO^	Simon et al. ^79^ / JAX	Strain: 025698
Cx40^Flox^	Chadjichristos et al. ^75^ / JAX	Strain: 039730
Cx40^GFP^	Miquerol et al. ^24^	N/A
Cx43^Flox^	Liao et al. ^78^ / JAX	Strain: 008039
Cx43^Flox-KI-CFP^	Degen et al. ^25^ / EMMA	Strain: 06788
Cx45^Flox-KI-GFP^	Maxeiner et al. ^26^ / EMMA	Strain: 01647
Bmx:Cre^ERT2^	Ehling et al. ^68^ / EMMA	Strain: 15026
Tie2:Cre	Kisanuki et al. ^80^ / JAX	Strain: 008863
Actb:Cre	Lewandoski et al. ^81^ / JAX	Strain: 019099
R26:CAG-Sun1/sfGFP	Mo et al. ^82^ / JAX	Strain: 021039
Ai65F	Daigle et al. ^83^ / JAX	Strain: 032864
R26:FLPe	Farley et al. ^84^ / JAX	Strain: 009086
**Oligonucleotides**		
Primers for Genotyping; see Table S2	Eton Biosciences	N/A
**Recombinant DNA**		
pAAV-CAG-fDIO(SERT-HA)-miR122-WPRE-pA	This Study / Addgene	Cat: 220937
pAAV-CAG-FLPo-miR122-WPRE-pA	This Study / Addgene	Cat: 220938
pAAV-Syn-jGCaMP8m-WPRE	Zhang et al. ^85^ / Addgene	Cat: 162375
pAAV-Syn-ChRmine-mScarlet-Kv2.1-WPRE	Marshel et al. ^32^ / Addgene	Cat: 130995
CMV-Cx40◦GFP	This Study	N/A
**Software and algorithms**		
(Fiji is Just) ImageJ 2.0.0-rc-69	Schindelin et al. ^86^	https://fiji.sc/
Adobe Illustrator 24.2	Adobe	https://www.adobe.com/products/illustrator.html
MATLAB (Version 2023b)	MathWorks	https://www.mathworks.com/products/matlab.html
Python (3.9.12)	Python Software Foundation	https://www.python.org/
CellProfiler (4.2.4)	McQuin et al. ^87^	https://cellprofiler.org/
GraphPad Prism (9.1.1)	GraphPad	https://www.graphpad.com/
Clampfit (10.7)	Molecular Devices	https://www.moleculardevices.com/products/axon-patch-clamp-system/acquisition-and-analysis-software/pclamp-software-suite#overview
ScanImage (2022)	MBF Bioscience	https://www.mbfbioscience.com/products/scanimage/
Psychophysics Toolbox (Version 3)	Psychtoolbox	http://psychtoolbox.org/
CODA Data Acquisition Software (4.1.0.0)	Kent Scientific	https://www.kentscientific.com/
**Other**		
N / A		

## Highlights

- Non-invasive tracing method for *in vivo* visualization of gap junction coupling
- Connexin expression and cell-cell coupling strength vary along arterio-venous axis
- Arterial gap junction coupling enables neurally evoked long-range dilations
- Endothelial gap junctions set the speed and spatial extent of vasodilation spread

## References

[1] Attwell D, and Laughlin SB (2001). An Energy Budget for Signaling in the Grey Matter of the Brain. J Cereb Blood Flow Metab 21, 1133–1145. 11598490 10.1097/00004647-200110000-00001

[2] Lennie P (2003). The Cost of Cortical Computation. Curr Biol 13, 493–497. 12646132 10.1016/s0960-9822(03)00135-0

[3] Sokoloff L, Mangold R, Wechsler RL, Kennedy C, and Kety SS (1955). The Effect of Mental Arithmetic on Cerebral Circulation and Metabolism. J Clin Invest 34, 1101–1108. 14392225 10.1172/JCI103159 PMC438861

[4] Bruckmaier M, Tachtsidis I, Phan P, and Lavie N (2020). Attention and Capacity Limits in Perception: A Cellular Metabolism Account. J Neurosci 40, 6801–6811. 32747442 10.1523/JNEUROSCI.2368-19.2020 PMC7455219

[5] Hillman EMC (2014). Coupling Mechanism and Significance of the BOLD signal: A Status Report. Annu Rev Neurosci 37, 161–181. 25032494 10.1146/annurev-neuro-071013-014111 PMC4147398

[6] Iadecola C (2017). The Neurovascular Unit Coming of Age: A Journey through Neurovascular Coupling in Health and Disease. Neuron 96, 17–42. 28957666 10.1016/j.neuron.2017.07.030 PMC5657612

[7] Sweeney MD, Kisler K, Montagne A, Toga AW, and Zlokovic BV (2018). The Role of Brain Vasculature in Neurodegenerative Disorders. Nat Neurosci 21, 1318–1331. 30250261 10.1038/s41593-018-0234-x PMC6198802

[8] Hill RA, Tong L, Yuan P, Murikinati S, Gupta S, and Grutzendler J (2015). Regional Blood Flow in the Normal and Ischemic Brain Is Controlled by Arteriolar Smooth Muscle Cell Contractility and Not by Capillary Pericytes. Neuron 87, 95–110. 26119027 10.1016/j.neuron.2015.06.001 PMC4487786

[9] Segal SS (1992). Communication Among Endothelial and Smooth Muscle Cells Coordinates Blood Flow Control During Exercise. Physiology 7, 152–156.

[10] Iadecola C, Yang G, Ebner TJ, and Chen AG (1997). Local and Propagated Vascular Responses Evoked by Focal Synaptic Activity in Cerebellar Cortex. J Neurophysiol 78, 651–659. 9307102 10.1152/jn.1997.78.2.651

[11] Chen BR, Bouchard MB, McCaslin AFH, Burgess SA, and Hillman EMC (2011). High-Speed Vascular Dynamics of the Hemodynamic Response. Neuroimage 54, 1021–1030. 20858545 10.1016/j.neuroimage.2010.09.036 PMC3018836

[12] Berwick J, Johnston D, Jones M, Martindale J, Martin C, Kennerley AJ, Redgrave P, and Mayhew JEW (2008). Fine Detail of Neurovascular Coupling Revealed by Spatiotemporal Analysis of the Hemodynamic Response to Single Whisker Stimulation in Rat Barrel Cortex. J Neurophysiol 99, 787–798. 18046008 10.1152/jn.00658.2007 PMC2652198

[13] Goodenough DA, and Paul DL (2009). Gap Junctions. Cold Spring Harb Perspect Biol 1, a002576. 20066080 10.1101/cshperspect.a002576 PMC2742079

[14] Longden TA, Dabertrand F, Koide M, Gonzales AL, Tykocki NR, Brayden JE, Hill-Eubanks D, and Nelson MT (2017). Capillary K+-Sensing Initiates Retrograde Hyperpolarization to Increase Local Cerebral Blood Flow. Nat Neurosci 20, 717–726. 28319610 10.1038/nn.4533 PMC5404963

[15] Thakore P, Alvarado MG, Ali S, Mughal A, Pires PW, Yamasaki E, Pritchard HAT, Isakson BE, Tran CHT, and Earley S (2021). Brain Endothelial Cell TRPA1 Channels Initiate Neurovascular Coupling. Elife 10, e63040. 33635784 10.7554/eLife.63040 PMC7935492

[16] Sancho M, Klug NR, Mughal A, Koide M, Huerta de la Cruz S, Heppner TJ, Bonev AD, Hill-Eubanks D, and Nelson MT (2022). Adenosine Signaling Activates ATP-Sensitive K+ Channels in Endothelial Cells and Pericytes in CNS Capillaries. Sci Signal 15, eabl5405. 35349300 10.1126/scisignal.abl5405 PMC9623876

[17] Hariharan A, Robertson CD, Garcia DCG, and Longden TA (2022). Brain Capillary Pericytes are Metabolic Sentinels That Control Blood Flow Through a KATP Channel-Dependent Energy Switch. Cell Rep 41, 111872. 36577387 10.1016/j.celrep.2022.111872 PMC10187957

[18] Chen BR, Kozberg MG, Bouchard MB, Shaik MA, and Hillman EMC (2014). A Critical Role for the Vascular Endothelium in Functional Neurovascular Coupling in the Brain. J Am Heart Assoc 3, e000787. 24926076 10.1161/JAHA.114.000787 PMC4309064

[19] Chow BW, Nuñez V, Kaplan L, Granger AJ, Bistrong K, Zucker HL, Kumar P, Sabatini BL, and Gu C (2020). Caveolae in CNS Arterioles Mediate Neurovascular Coupling. Nature 579, 106–110. 32076269 10.1038/s41586-020-2026-1 PMC7060132

[20] Hou M, Li Y, and Paul DL (2013). A Novel, Highly Sensitive Method for Assessing Gap Junctional Coupling. J Neurosci Methods 220, 18–23. 23958747 10.1016/j.jneumeth.2013.08.007 PMC3808728

[21] Chan KY, Jang MJ, Yoo BB, Greenbaum A, Ravi N, Wu WL, Sánchez-Guardado L, Lois C, Mazmanian SK, Deverman BE, (2017). Engineered AAVs for Efficient Noninvasive Gene Delivery to the Central and Peripheral Nervous Systems. Nat Neuro 20, 1172–1179. 10.1038/nn.4593 PMC5529245 28671695

[22] Krolak T, Chan KY, Kaplan L, Huang Q, Wu J, Zheng Q, Kozareva V, Beddow T, Tobey IG, Pacouret S, (2022). A High-Efficiency AAV for Endothelial Cell Transduction Throughout the Central Nervous System. Nature Cardiovascular Research 1, 389–400. 10.1038/s44161-022-00046-4 PMC9103166 35571675

[23] Vanlandewijck M, He L, Mäe MA, Andrae J, Ando K, Del Gaudio F, Nahar K, Lebouvier T, Laviña B, Gouveia L, (2018). A Molecular Atlas of Cell Types and Zonation in the Brain Vasculature. Nature 554, 475–480. 29443965 10.1038/nature25739

[24] Miquerol L, Meysen S, Mangoni M, Bois P, Van Rijen HVM, Abran P, Jongsma H, Nargeot J, and Gros D (2004). Architectural and Functional Asymmetry of the His-Purkinje System of the Murine Heart. Cardiovasc Res 63, 77–86. 15194464 10.1016/j.cardiores.2004.03.007

[25] Degen J, Dublin P, Zhang J, Dobrowolski R, Jokwitz M, Karram K, Trotter J, Jabs R, Willecke K, Steinhäuser C, (2012). Dual Reporter Approaches for Identification of Cre Efficacy and Astrocyte Heterogeneity. FASEB Journal 26, 4576–4583. 22859373 10.1096/fj.12-207183

[26] Maxeiner S, Dedek K, Janssen-Bienhold U, Ammermüller J, Brune H, Kirsch T, Pieper M, Degen J, Krüger O, Willecke K, (2005). Deletion of Connexin45 in Mouse Retinal Neurons Disrupts the Rod/Cone Signaling Pathway Between AII Amacrine and ON Cone Bipolar Cells and Leads to Impaired Visual Transmission. J Neurosci 25, 566–576. 15659592 10.1523/JNEUROSCI.3232-04.2005 PMC6725315

[27] Jeong HW, Diéguez-Hurtado R, Arf H, Song J, Park H, Kruse K, Sorokin L, and Adams RH (2022). Single-Cell Transcriptomics Reveals Functionally Specialized Vascular Endothelium in Brain. Elife 11. 10.7554/eLife.57520 PMC9566870 36197007

[28] Krüger O, Bény J-L, Chabaud F, Traub O, Theis M, Brix K, Kirchhoff S, and Willecke K (2002). Altered Dye Diffusion and Upregulation of Connexin37 in Mouse Aortic Endothelium Deficient in Connexin40. J Vasc Res 39, 160–172. 12011587 10.1159/000057764

[29] Simon AM, and McWhorter AR (2003). Decreased Intercellular Dye-Transfer and Downregulation of Non-Ablated Connexins in Aortic Endothelium Deficient in Connexin37 or Connexin40. J Cell Sci 116, 2223–2236. 12697838 10.1242/jcs.00429

[30] Zhuang J, Ng L, Williams D, Valley M, Li Y, Garrett M, and Waters J (2017). An Extended Retinotopic Map of Mouse Cortex. Elife 6, e18372. 28059700 10.7554/eLife.18372 PMC5218535

[31] Ma Y, Shaik MA, Kim SH, Kozberg MG, Thibodeaux DN, Zhao HT, Yu H, and Hillman EMC (2016). Wide-Field Optical Mapping of Neural Activity and Brain Haemodynamics: Considerations and Novel Approaches. Philos Trans R Soc Lond B Biol Sci 371. 10.1098/rstb.2015.0360 PMC5003860 27574312

[32] Marshel JH, Seok Kim Y, Machado TA, Quirin S, Benson B, Kadmon J, Raja C, Chibukhchyan A, Ramakrishnan C, Inoue M, (2019). Cortical Layer-Specific Critical Dynamics Triggering Perception. Science (1979) 365, eaaw5202. 10.1126/science.aaw5202 PMC6711485 31320556

[33] Tian P, Teng IC, May LD, Kurz R, Lu K, Scadeng M, Hillman EMC, De Crespigny AJ, D’Arceuil HE, Mandeville JB, (2010). Cortical Depth-Specific Microvascular Dilation Underlies Laminar Differences in Blood Oxygenation Level-Dependent Functional MRI Signal. Proc Natl Acad Sci U S A 107, 15246–15251. 20696904 10.1073/pnas.1006735107 PMC2930564

[34] Uhlirova H, Kılıç K, Tian P, Thunemann M, le Desjardins M, Saisan PA, Mateo C, Cheng Q, Weldy KL, Razoux F, (2016). Cell Type Specificity of Neurovascular Coupling in Cerebral Cortex. Elife, e14315. 27244241 10.7554/eLife.14315 PMC4933561

[35] Berg BR, Cohen KD, and Sarelius IH (1997). Direct Coupling Between Blood Flow and Metabolism at the Capillary Level in Striated Muscle. Am J Physiol 272, H2693–2700. 9227548 10.1152/ajpheart.1997.272.6.H2693

[36] Hogan-Cann AD, Lu P, and Anderson CM (2019). Endothelial NMDA Receptors Mediate Activity-Dependent Brain Hemodynamic Responses in Mice. Proc Natl Acad Sci U S A 116, 10229–10231. 31061120 10.1073/pnas.1902647116 PMC6535036

[37] Rosehart AC, Longden TA, Weir N, Fontaine JT, Joutel A, and Dabertrand F (2021). Prostaglandin E2 Dilates Intracerebral Arterioles When Applied to Capillaries: Implications for Small Vessel Diseases. Front Aging Neurosci 13. 10.3389/fnagi.2021.695965 PMC8414797 34483880

[38] Ando K, Tong L, Peng D, Vázquez-Liébanas E, Chiyoda H, He L, Liu J, Kawakami K, Mochizuki N, Fukuhara S, (2022). KCNJ8/ABCC9-Containing K-ATP Channel Modulates Brain Vascular Smooth Muscle Development and Neurovascular Coupling. Dev Cell 57, 1383–1399.e7. 35588738 10.1016/j.devcel.2022.04.019

[39] Isaacs D, Xiang L, Hariharan A, and Longden TA (2024). KATP Channel-Dependent Electrical Signaling Links Capillary Pericytes to Arterioles During Neurovascular Coupling. Proc Natl Acad Sci U S A 121, e2405965121. 39630860 10.1073/pnas.2405965121 PMC11648664

[40] Emerson GG, and Segal SS (2000). Endothelial Cell Pathway for Conduction of Hyperpolarization and Vasodilation Along Hamster Feed Artery. Circ Res 86, 94–100. 10625310 10.1161/01.res.86.1.94

[41] Bando Y, Grimm C, Cornejo VH, and Yuste R (2019). Genetic Voltage Indicators. BMC Biol 17. 10.1186/s12915-019-0682-0 PMC6739974 31514747

[42] Yamamoto Y, Klemm MF, Edwards FR, and Suzuki H (2001). Intercellular Electrical Communication Among Smooth Muscle and Endothelial cells in Guinea-Pig Mesenteric Arterioles. J Physiol 535, 181–195. 11507168 10.1111/j.1469-7793.2001.00181.x PMC2278769

[43] Sandow SL, Looft-Wilson R, Doran B, Grayson TH, Segal SS, and Hill CE (2003). Expression of Homocellular and Heterocellular Gap Junctions in Hamster Arterioles and Feed Arteries. Cardiovasc Res 60, 643–653. 14659810 10.1016/j.cardiores.2003.09.017

[44] Welsh DG, Segal SS, and Segal Endothelial and SS (1998). Endothelial and Smooth Muscle Cell Conduction in Arterioles Controlling Blood Flow. Am J Physiol, H178–86. 9458866 10.1152/ajpheart.1998.274.1.H178

[45] De Wit C (2010). Different Pathways with Distinct Properties Conduct Dilations in the Microcirculation In Vivo. Cardiovasc Res 85, 604–613. 19820254 10.1093/cvr/cvp340

[46] Bartlett IS, and Segal SS (2000). Resolution of Smooth Muscle and Endothelial Pathways for Conduction Along Hamster Cheek Pouch Arterioles. Am J Physiol Heart Circ Physiol 278, H604–12. 10666093 10.1152/ajpheart.2000.278.2.H604

[47] Niwa K, Araki E, Morham SG, Ross ME, and Iadecola C (2000). Cyclooxygenase-2 Contributes to Functional Hyperemia in Whisker-Barrel Cortex. J Neurosci 20, 763–770. 10632605 10.1523/JNEUROSCI.20-02-00763.2000 PMC6772412

[48] Yang G, Zhang Y, Ross ME, Iadecola C, Yang Y, Zhang ME, and Ross C (2003). Attenuation of Activity-Induced Increases in Cerebellar Blood Flow in Mice Lacking Neuronal Nitric Oxide Synthase. Am J Physiol Heart Circ Physiol 285, 298–304. 10.1152/ajpheart.00043.2003 12623792

[49] Attwell D, Buchan AM, Charpak S, Lauritzen M, MacVicar BA, and Newman EA (2010). Glial and Neuronal Control of Brain Blood Flow. Preprint, 10.1038/nature09613. PMC3206737 21068832

[50] Lacroix A, Toussay X, Anenberg E, Lecrux C, Ferreirós N, Karagiannis A, Plaisier F, Chausson P, Jarlier F, Burgess SA, (2015). COX-2-Derived Prostaglandin E2 Produced by Pyramidal Neurons Contributes to Neurovascular Coupling in the Rodent Cerebral Cortex. J Neurosci 35, 11791–11810. 26311764 10.1523/JNEUROSCI.0651-15.2015 PMC6705452

[51] Zhang D, Ruan J, Peng S, Li J, Hu X, Zhang Y, Zhang T, Ge Y, Zhu Z, Xiao X, (2024). Synaptic-Like Transmission Between Neural Axons and Arteriolar Smooth Muscle Cells Drives Cerebral Neurovascular Coupling. Nat Neurosci 27, 232–248. 38168932 10.1038/s41593-023-01515-0 PMC10849963

[52] Hosford PS, and Gourine AV (2019). What is the Key Mediator of the Neurovascular Coupling Response? Neurosci Biobehav Rev 96, 174–181. 30481531 10.1016/j.neubiorev.2018.11.011 PMC6331662

[53] Elfgang C, Eckert R, Lichtenberg-Frat~ H, Butterweck A, Traub O, Klein RA, Hiilser DE, and Willecke K (1995). Specific Permeability and Selective Formation of Gap Junction Channels in Connexin-Transfected HeLa Cells. J Cell Biol 129, 805–817. 7537274 10.1083/jcb.129.3.805 PMC2120441

[54] Söhl G, and Willecke K (2004). Gap Junctions and the Connexin Protein Family. Cardiovasc Res 62, 228–232. 15094343 10.1016/j.cardiores.2003.11.013

[55] Moreno AP, Laing JG, Beyer EC, and Spray DC (1995). Properties of Gap Junction Channels Formed of Connexin 45 Endogenously Expressed in Human Hepatoma (SKHepl) Cells. Am J Physiol 268, C356–65. 7532358 10.1152/ajpcell.1995.268.2.C356

[56] Traub O, Hertleina B, Kasper M, Eckertc R, Krisciukaitisc A, Htilser D, Willecke K, and Traub O (1998). Characterization of the Gap Junction Protein Connexin37 in Murine Endothelium, Respiratory Epithelium, and After Transfection in Human HeLa Cells. Eur J Cell Biol 77, 313–322. 9930656 10.1016/S0171-9335(98)80090-3

[57] Stümpel F, Ott T, Willecke K, and Jungermann K (1998). Connexin 32 Gap Junctions Enhance Stimulation of Glucose Output by Glucagon and Noradrenaline in Mouse Liver. Hepatology 28, 1616–1620. 9828226 10.1002/hep.510280622

[58] Ablasser A, Schmid-Burgk JL, Hemmerling I, Horvath GL, Schmidt T, Latz E, and Hornung V (2013). Cell Intrinsic Immunity Spreads to Bystander Cells via the Intercellular Transfer of cGAMP. Nature 503, 530–534. 24077100 10.1038/nature12640 PMC4142317

[59] Trenholm S, and Awatramani GB (2019). Myriad Roles for Gap Junctions in Retinal Circuits. In Webvision: The Organization of the Retina and Visual System. 31765113

[60] Monterisi S, Michl J, Hulikova A, Koth J, Bridges EM, Hill AE, Abdullayeva G, Bodmer WF, and Swietach P (2022). Solute Exchange Through Gap Junctions Lessens the Adverse Effects of Inactivating Mutations in Metabolite-Handling Genes. Elife 11, e78425. 36107487 10.7554/eLife.78425 PMC9534548

[61] Kreuzberg MM, Willecke K, and Bukauskas FF (2006). Connexin-Mediated Cardiac Impulse Propagation: Connexin 30.2 Slows Atrioventricular Conduction in Mouse Heart. Trends Cardiovasc Med 16, 266–272. 17055382 10.1016/j.tcm.2006.05.002 PMC3615414

[62] Verheule S, and Kaese S (2013). Connexin Diversity in the Heart: Insights From Transgenic Mouse Models. Front Pharmacol 4, 10.3389/fphar.2013.00081. PMC3694209 23818881

[63] Challis RC, Kumar SR, Chen X, Goertsen D, Coughlin GM, Hori AM, Chuapoco MR, Otis TS, Miles TF, and Gradinaru V (2022). Adeno-Associated Virus Toolkit to Target Diverse Brain Cells. Annu Rev Neurosci 8, 447–469. 10.1146/annurev-neuro-111020-100834 35440143

[64] Broggini T, Duckworth J, Ji X, Liu R, Xia X, Mächler P, Shaked I, Munting LP, Iyengar S, Kotlikoff M, (2024). Long-Wavelength Traveling Waves of Vasomotion Modulate the Perfusion of Cortex. Neuron 112, 2349–2367.e8. 38781972 10.1016/j.neuron.2024.04.034 PMC11257831

[65] Zhao Y, Vanhoutte PM, and Leung SWS (2015). Vascular Nitric Oxide: Beyond eNOS. J Pharmacol Sci 129, 83–94. 26499181 10.1016/j.jphs.2015.09.002

[66] Nelson MT, Patlak JB, Worley JF, and Standen NB (1990). Calcium Channels, Potassium Channels, and Voltage Dependence of Arterial Smooth Muscle Tone. Am J Physiol 259, C3–18. 2164782 10.1152/ajpcell.1990.259.1.C3

[67] Knot HJ, and Nelson MT (1998). Regulation of Arterial Diameter and Wall [Ca2+] in Cerebral Arteries of Rat by Membrane Potential and Intravascular Pressure. J Physiol 508, 199–209. 9490839 10.1111/j.1469-7793.1998.199br.x PMC2230857

[68] Ehling M, Adams S, Benedito R, and Adams RH (2013). Notch Controls Retinal Blood Vessel Maturation and Quiescence. Development 140, 3051–3061. 23785053 10.1242/dev.093351

[69] Thiriot A, Perdomo C, Cheng G, Novitzky-Basso I, McArdle S, Kishimoto JK, Barreiro O, Mazo I, Triboulet R, Ley K, (2017). Differential DARC/ACKR1 Expression Distinguishes Venular From Non-Venular Endothelial Cells in Murine Tissues. BMC Biol 15. 10.1186/s12915-017-0381-7 PMC5438556 28526034

[70] Lee HW, Xu Y, He L, Choi W, Gonzalez D, Jin SW, and Simons M (2021). Role of Venous Endothelial Cells in Developmental and Pathologic Angiogenesis. Circulation 144, 1308–1322. 34474596 10.1161/CIRCULATIONAHA.121.054071 PMC9153651

[71] Willecke K, Heynkes R, Dahl E, Stutenkemper R, Hennemann H, Jungbluth S, Suchyna T, and Nicholson BJ (1991). Mouse Connexin37: Cloning and Functional Expression of a Gap Junction Gene Highly Expressed in Lung. J Cell Biol 114, 1049–1057. 1651942 10.1083/jcb.114.5.1049 PMC2289105

[72] Kalucka J, de Rooij LPMH, Goveia J, Rohlenova K, Dumas SJ, Meta E, Conchinha NV, Taverna F, Teuwen LA, Veys K, (2020). Single-Cell Transcriptome Atlas of Murine Endothelial Cells. Cell 180, 764–779.e20. 32059779 10.1016/j.cell.2020.01.015

[73] Alonso F, Boittin F-X, Bény J-L, and Haefliger J-A (2010). Loss of Connexin40 is Associated with Decreased Endothelium-Dependent Relaxations and eNOS Levels in the Mouse Aorta. Am J Physiol Heart Circ Physiol 299, 1365–1373. 10.1152/ajpheart.00029.2010 20802140

[74] Meens MJ, Alonso F, Le Gal L, Kwak BR, and Haefliger JA (2015). Endothelial Connexin37 and Connexin40 Participate in Basal but Not Agonist-Induced NO Release. Cell Commun Signal 13. 10.1186/s12964-015-0110-1 PMC4510910 26198171

[75] Chadjichristos CE, Scheckenbach KEL, Van Veen TAB, Richani Sarieddine MZ, De Wit C, Yang Z, Roth I, Bacchetta M, Viswambharan H, Foglia B, (2010). Endothelial-Specific Deletion of Connexin40 Promotes Atherosclerosis by Increasing CD73-Dependent Leukocyte Adhesion. Circulation 121, 123–131. 20026782 10.1161/CIRCULATIONAHA.109.867176

[76] Isakson BE, Damon DN, Day KH, Liao Y, and Duling BR (2006). Connexin40 and Connexin43 in Mouse Aortic Endothelium: Evidence for Coordinated Regulation. Am J Physiol Heart Circ Physiol 290, 1199–1205. 10.1152/ajpheart.00945.2005 16284228

[77] Zechariah A, Tran CHT, Hald BO, Sandow SL, Sancho M, Kim MSM, Fabris S, Tuor UI, Gordon GRJ, and Welsh DG (2020). Intercellular Conduction Optimizes Arterial Network Function and Conserves Blood Flow Homeostasis During Cerebrovascular Challenges. Arterioscler Thromb Vasc Biol 40, 733–750. 31826653 10.1161/ATVBAHA.119.313391 PMC7058668

[78] Liao Y, Day KH, Damon DN, and Duling BR (2001). Endothelial Cell-Specific Knockout of Connexin 43 Causes Hypotension and Bradycardia in Mice. Proc Natl Acad Sci U S A 98, 9989–9994. 11481448 10.1073/pnas.171305298 PMC55565

[79] Simon AM, Goodenough DA, Li E, and Paul DL (1997). Female Infertility in Mice Lacking Connexin 37. Nature 385, 525–529. 9020357 10.1038/385525a0

[80] Kisanuki YY, Hammer RE, Miyazaki J ichi, Williams SC, Richardson JA, and Yanagisawa M (2001). Tie2-Cre Transgenic Mice: A New Model for Endothelial Cell-Lineage Analysis In Vivo. Dev Biol 230, 230–242. 11161575 10.1006/dbio.2000.0106

[81] Lewandoski M, Meyers EN, and Martin GR (1997). Analysis of Fgf8 Gene Function in Vertebrate Development. Cold Spring Harb Symp Quant Biol 62, 159–168. 9598348

[82] Mo A, Mukamel EA, Davis FP, Luo C, Henry GL, Picard S, Urich MA, Nery JR, Sejnowski TJ, Lister R, (2015). Epigenomic Signatures of Neuronal Diversity in the Mammalian Brain. Neuron 86, 1369–1384. 26087164 10.1016/j.neuron.2015.05.018 PMC4499463

[83] Daigle TL, Madisen L, Hage TA, Valley MT, Knoblich U, Larsen RS, Takeno MM, Huang L, Gu H, Larsen R, (2018). A Suite of Transgenic Driver and Reporter Mouse Lines with Enhanced Brain-Cell-Type Targeting and Functionality. Cell 174, 465–480.e22. 30007418 10.1016/j.cell.2018.06.035 PMC6086366

[84] Farley FW, Soriano P, Steffen LS, and Dymecki SM (2000). Widespread Recombinase Expression Using FLPeR (Flipper) Mice. Genesis 28, 106–110. 11105051

[85] Zhang Y, Rózsa M, Liang Y, Bushey D, Wei Z, Zheng J, Reep D, Broussard GJ, Tsang A, Tsegaye G, (2023). Fast and Sensitive GCaMP Calcium Indicators for Imaging Neural Populations. Nature 615, 884–891. 36922596 10.1038/s41586-023-05828-9 PMC10060165

[86] Schindelin J, Arganda-Carreras I, Frise E, Kaynig V, Longair M, Pietzsch T, Preibisch S, Rueden C, Saalfeld S, Schmid B, (2012). Fiji: An Open-Source Platform for Biological-Image Analysis. Nat Methods 9, 676–682. 22743772 10.1038/nmeth.2019 PMC3855844

[87] McQuin C, Goodman A, Chernyshev V, Kamentsky L, Cimini BA, Karhohs KW, Doan M, Ding L, Rafelski SM, Thirstrup D, (2018). CellProfiler 3.0: Next-Generation Image Processing for Biology. PLoS Biol 16. 10.1371/journal.pbio.2005970 PMC6029841 29969450

[88] Benes J Jr, Ammirabile G, Sankova B, Campione M, Krejci E, Kvasilova A, and Sedmera D (2014). The Role of Connexin40 in Developing Atrial Conduction. FEBS Lett 588, 1465–1469. 24486905 10.1016/j.febslet.2014.01.032

[89] Malumbres M, Mangues R, Ferrer N, Lu S, and Pellicer A (1997). Isolation of High Molecular Weight DNA for Reliable Genotyping of Transgenic Mice. Biotechniques 22, 1114–1119. 9187761 10.2144/97226st03

[90] Skarnes WC, Rosen B, West AP, Koutsourakis M, Bushell W, Iyer V, Mujica AO, Thomas M, Harrow J, Cox T, (2011). A Conditional Knockout Resource for the Genome-Wide Study of Mouse Gene Function. Nature 474, 337–344. 21677750 10.1038/nature10163 PMC3572410

[91] Ramamoorthy S, Baumant AL, Mooret KR, Han4 H, Yang-Feng T, Chang AS, Ganapathy V, and Blakelyt RD (1993). Antidepressant- and Cocaine-Sensitive Human Serotonin Transporter: Molecular Cloning, Expression, and Chromosomal Localization. Proc Natl Acad Sci USA 90, 2542–2546. 7681602 10.1073/pnas.90.6.2542 PMC46124

[92] Xue M, Atallah BV, and Scanziani M (2014). Equalizing Excitation-Inhibition Ratios Across Visual Cortical Neurons. Nature 511, 596–600. 25043046 10.1038/nature13321 PMC4117808

[93] Pfau SJ, Langen UH, Fisher TM, Prakash I, Nagpurwala F, Lozoya RA, Lee WCA, Wu Z, and Gu C (2024). Characteristics of Blood–Brain Barrier Heterogeneity Between Brain Regions Revealed by Profiling Vascular and Perivascular Cells. Nat Neurosci10.1038/s41593-024-01743-y PMC11452347 39210068

[94] Challis RC, Ravindra Kumar S, Chan KY, Challis C, Beadle K, Jang MJ, Kim HM, Rajendran PS, Tompkins JD, Shivkumar K, (2019). Systemic AAV Vectors for Widespread and Targeted Gene Delivery in Rodents. Nat Protoc 14, 379–414. 30626963 10.1038/s41596-018-0097-3 PMC13333184

[95] Ayuso E, Blouin V, Lock M, Mcgorray S, Leon X, Alvira MR, Auricchio A, Bucher S, Chtarto A, Clark KR, (2014). Manufacturing and Characterization of a Recombinant Adeno-Associated Virus Type 8 Reference Standard Material. Hum Gene Ther 25, 977–987. 25275822 10.1089/hum.2014.057 PMC4236062

[96] Körbelin J, Dogbevia G, Michelfelder S, Ridder DA, Hunger A, Wenzel J, Seismann H, Lampe M, Bannach J, Pasparakis M, (2016). A Brain Microvasculature Endothelial Cell‐ Specific Viral Vector with the Potential to Treat Neurovascular and Neurological Diseases. EMBO Mol Med 8, 609–625. 27137490 10.15252/emmm.201506078 PMC4888852

[97] Sneddon JM (1973). Blood Platelets as a Model for Monoamine-Containing Neurones. Prog Neurobiol 1, 151–198. 4273118 10.1016/0301-0082(73)90019-1

[98] Chen JJ, Li Z, Pan H, Murphy DL, Tamir H, Koepsell H, and Gershon MD (2001). Maintenance of Serotonin in the Intestinal Mucosa and Ganglia of Mice that Lack the High-Affinity Serotonin Transporter: Abnormal Intestinal Motility and the Expression of Cation Transporters. J Neurosci 21, 6348–6361. 11487658 10.1523/JNEUROSCI.21-16-06348.2001 PMC6763151

[99] Anderson GM, Feibel FC, and Cohen DJ (1987). Determination of Serotonin in Whole Blood, Platelet-Rich Plasma, Platelet-Poor Plasma and Plasma Ultrafiltrate. Life Sci 40, 1063–1070. 3821372 10.1016/0024-3205(87)90568-6

[100] Steinbusch HW, Verhofstad AA, and Joosten HW (1978). Localization of Serotonin in the Central Nervous System by Immunohistochemistry: Description of a Specific and Sensitive Technique and Some Applications. Neuroscience 3, 811–819. 362232 10.1016/0306-4522(78)90033-7

[101] Thul PJ, Akesson L, Wiking M, Mahdessian D, Geladaki A, Ait Blal H, Alm T, Asplund A, Björk L, Breckels LM, (2017). A Subcellular Map of the Human Proteome. Science (1979) 356, eaal3321. 10.1126/science.aal3321 28495876

[102] Boadle-Biber MC (1993). Regulation of Serotonin Synthesis. Prog Biophys Mol Biol 60, 1–15. 8480026 10.1016/0079-6107(93)90009-9

[103] Sonkusare SK, Dalsgaard T, Bonev AD, and Nelson MT (2016). Inward Rectifier Potassium (Kir2.1) Channels as End-Stage Boosters of Endothelium-Dependent Vasodilators. J Physiol 594, 3271–3285. 26840527 10.1113/JP271652 PMC4908010

[104] Goldey GJ, Roumis DK, Glickfeld LL, Kerlin AM, Reid RC, Bonin V, Schafer DP, and Andermann ML (2014). Removable Cranial Windows for Long-Term Imaging in Awake Mice. Nat Protoc 9, 2515–2538. 25275789 10.1038/nprot.2014.165 PMC4442707

[105] Liang L, Fratzl A, Goldey G, Ramesh RN, Sugden AU, Morgan JL, Chen C, and Andermann ML (2018). A Fine-Scale Functional Logic to Convergence from Retina to Thalamus. Cell 173, 1343–1355.e24. 29856953 10.1016/j.cell.2018.04.041 PMC6003778

[106] Bouchard MB, Chen BR, Burgess SA, C Hillman EM, Kwong KK, Belliveau JW, Chesler DA, Goldberg IE, Weisskoff RM, Poncelet BP, (2009). Ultra-Fast Multispectral Optical Imaging of Cortical Oxygenation, Blood Flow, and Intracellular Calcium Dynamics. Opt Express 17, 15670–15678. 19724566 10.1364/OE.17.015670 PMC2760073

[107] Gao YR, and Drew PJ (2014). Determination of Vessel Cross-Sectional Area by Thresholding in Radon Space. J Cereb Blood Flow Metab 34, 1180–1187. 24736890 10.1038/jcbfm.2014.67 PMC4083381

[108] Chhatbar PY, and Kara P (2013). Improved Blood Velocity Measurements with a Hybrid Image Filtering and Iterative Radon Transform Algorithm. Front Neurosci 7. 10.3389/fnins.2013.00106 PMC3684769 23807877

[109] Niell CM, and Stryker MP (2010). Modulation of Visual Responses by Behavioral State in Mouse Visual Cortex. Neuron 65, 472–479. 20188652 10.1016/j.neuron.2010.01.033 PMC3184003

[110] Eyre B, Shaw K, Sharp P, Boorman L, Lee L, Shabir O, Berwick J, and Howarth C (2022). The Effects of Locomotion on Sensory-Evoked Haemodynamic Responses in the Cortex of Awake Mice. Sci Rep 12. 10.1038/s41598-022-10195-y PMC9010417 35422473

[111] Strauch C, Wang CA, Einhäuser W, Van der Stigchel S, and Naber M (2022). Pupillometry as an Integrated Readout of Distinct Attentional Networks. Trends Neurosci 45, 635–647. 35662511 10.1016/j.tins.2022.05.003

[112] Sirotin YB, and Das A (2009). Anticipatory Haemodynamic Signals in Sensory Cortex Not Predicted by Local Neuronal Activity. Nature 457, 475–479. 19158795 10.1038/nature07664 PMC2705195

[113] Das A, Murphy K, and Drew PJ (2021). Rude Mechanicals in Brain Haemodynamics: Non-Neural Actors That Influence Blood Flow. Philos Trans R Soc Lond B Biol Sci 376. 10.1098/rstb.2019.0635 PMC7741032 33190603

[114] Aarts E, Verhage M, Veenvliet JV, Dolan CV, and Van Der Sluis S (2014). A Solution to Dependency: Using Multilevel Analysis to Accommodate Nested Data. Nat Neurosci 17, 491–496. 24671065 10.1038/nn.3648

[115] Snijders TAB, and Bosker RJ (2012). Multilevel Analysis: An Introduction to Basic and Advanced Multilevel Modeling, second edition (American Psychological Association Inc.).

[116] Feng M, Whitesall S, Zhang Y, Beibel M, D’Alecy L, and DiPetrillo K (2008). Validation of Volume-Pressure Recording Tail-Cuff Blood Pressure Measurements. Am J Hypertens 21, 1288–1291. 18846043 10.1038/ajh.2008.301

[117] Li P, Sur SH, Mistlberger RE, and Morris M (1999). Circadian Blood Pressure and Heart Rate Rhythms in Mice. Am J Physiol 276, R500–4. 9950930 10.1152/ajpregu.1999.276.2.R500

[118] Sheward WJ, Naylor E, Knowles-Barley S, Armstrong JD, Brooker GA, Seckl JR, Turek FW, Holmes MC, Zee PC, and Harmar AJ (2010). Circadian Control of Mouse Heart Rate and Blood Pressure by the Suprachiasmatic Nuclei: Behavioral Effects are More Significant Than Direct Outputs. PLoS One 5, e9783. 20339544 10.1371/journal.pone.0009783 PMC2842429

