# Supplementary material for: Brain Endothelial Gap Junction Coupling Enables Rapid Vasodilation Propagation During Neurovascular Coupling
Source: Cell. Author manuscript; Available in PMC 2025 Aug 11. (PMC12337775; doi:10.1016/j.cell.2025.06.030)
Supplement: 5 — Supplementary Table 1. Exact statistical test results for live imaging experiments. (Related to Figure 4E & H, Figure 5B, Figure 6C, and Figure S6G.) [file NIHMS2097217-supplement-5.pdf]

Supplementary Table 1. Exact statistical test results for live imaging experiments. (Related to **Figure 4E & H**, **Figure 5B**, **Figure 6C**, and **Figure S6G**.)

		**Baseline Diameter Correction Term?**	
**Paradigm**	**Distances (µm)**	**No**	**Yes**
18° Visual Stimulus (Two-Photon)	<1	0.0412	0.0014
[**Figure 4E**]	1-400	0.0004	0.0001
	400-800	0.0467	0.0017
	800-1200	0.0186	0.0141
	1200-1600	0.1038	0.0079
	1600-2000	0.8534	0.1644
	Blank Screen	0.7320	0.6776
18° Visual Stimulus (Widefield IOS)	<1	0.0003	0.0692
[**Figure 4H**]	1-400	< 0.0001	0.0009
	400-800	< 0.0001	0.0004
	800-1200	< 0.0001	0.2455
	1200-1600	0.0341	0.8571
	1600-2000	0.6923	0.3152
	2000-2500	0.9839	0.2203
	>2500	0.4524	0.3056
	Blank Screen	0.3863	0.4045
80mW/cm^2 Optogenetic Stimulus	<1	< 0.0001	< 0.0001
[**Figure 5B**]	1-500	< 0.0001	< 0.0001
	500-1000	< 0.0001	< 0.0001
	1000-1500	< 0.0001	0.0004
	1500-2000	0.0328	0.6822
	2000-2500	0.0641	0.7213
	0 mW/cm^2 Control Stim.	0.0013	0.0851
Full-Screen Visual Stimulus	<1	< 0.0001	< 0.0001
[**Figure 6C**]	1-400	< 0.0001	< 0.0001
	400-800	< 0.0001	< 0.0001
	800-1200	< 0.0001	0.0373
	1200-1600	0.1882	0.8886
	1600-2000	0.6427	0.6681
	Blank Screen	0.8968	0.2130
