# Supplementary material for: Brain Endothelial Gap Junction Coupling Enables Rapid Vasodilation Propagation During Neurovascular Coupling
Source: Cell. Author manuscript; Available in PMC 2025 Aug 11. (PMC12337775; doi:10.1016/j.cell.2025.06.030)
Supplement: 6 — Supplementary Table 2. Genotyping primers and expected band sizes for transgenic mice. (Related to the STAR Methods.) [file NIHMS2097217-supplement-6.pdf]

Supplementary Table 2. Genotyping primers and expected band sizes for transgenic mice. (Related to the **STAR Methods**.)

Strain	Ref.	F/R	Primer Sequence	Approx. Products
Cx37 KO	[79]	F	TTC CTG GAG AAG TTG CTA GAC C	400 bp = Wild Type<br>250 bp = Knockout
		R	CGA GAT CTT GGC CAT CTG A	
		R	GAT CTC TCG TGG GAT CAT TG	
Cx37 Flox		F	CAC CAC ACA TCA CCA CGT TTT CAC	535 bp = Floxed<br>385 bp = Wild Type
		R	CTT CCT CCC TTT TGT TCA AGG CTG	
Cx37 LacZ		F	CAC CAC ACA TCA CCA CGT TTT CAC	565 bp = LacZ<br>385 bp = Wild Type
		R	CTT CCT CCC TTT TGT TCA AGG CTG	
		F	GCT ACC ATT ACC AGT TGG TCT GGT GTC	
		R	CCT CAG CAA CAG GTG AAA AGG ATC G	
Cx40 Flox	[75]	F	GCC ATC CTC TGC TAC ATA TGC AG	700 bp = Floxed<br>555 bp = Wild Type
		R	GTG ACA TGA CCT GGA TCT CTG GAG	
Cx40 GFP	[24]	F	CTC CAA TTA ACT CCT TGT GAG CC	510 bp = GFP<br>420 bp = Wild Type
		R	AGG CTG AAT GGT ATC GCA CC	
		R	CTT GCC GAA TAT CAT GGT GG	
Cx43 Flox	[78]	F	CTT TGA CTC TGA TTA CAG AGC TTA A	580 bp = Floxed<br>490 bp = Wild Type
		R	GTC TCA CTG TTA CTT AAC AGC TTG A	
Cx43 Flox-KI-CFP	[25]	F	GCA CTT GGT AGG TAG AGC CTG TCA GGT C	700 bp = CFP<br>385 bp = Floxed<br>340 bp = Wild Type
		R	GCT TCC CCA AGG CGC TCC AGT CAC CC	
		R	AAG AAG TCG TGC TGC TTC ATG TGG	
Cx45 Flox-KI-GFP	[26]	F	GGA TTA AAG GCA TAT GTC ACC ACT CTT GGC	620 bp = GFP<br>475 bp = Floxed<br>390 bp = Wild Type
		R	CTC TAG GAA CAC TGT AAC CTG AGA TGT CCC	
		R	AAG AAC GGC CAC AAC TCT GGT AAC AGG AAG	
Tie2:Cre	[80]	F	CCC TGT GCT CAG ACA GAA ATG AGA	560 bp = Cre^+
		R	CGC ATA ACC AGT GAA ACA GCA TTG C	
Actb:Cre	[81]	F	ACC AGT TTC CAG TCC TTC TGG	240 bp = Wild Type<br>190 bp = Cre^+
		F	GTC CTT ACC CAG AGT GCA GGT	
		R	TGC AAT CCC TTG ACA CAG A	
BMX:Cre^{ERT2}	[68]	F	AGG AAG ATG GCG CAA ACA TGG	650 bp = Cre^+
		R	CCT GTT TTG CAC GTT CAC CG	
R26:FLPe	[84]	F	TGC CGG TCC TAT TTA CTC GT	240 bp = Wild Type<br>100 bp = FLP^+
		R	TAC TTC TTT AGC GCA AGG GGT AG	
		F	AAG GGA GCT GCA GTG GAG TA	
		R	CAG GAC AAC GCC CAC ACA	
R26:Sun1/sfGFP	[82]	F	ACA CTT GCC TCT ACC GGT TC	240 bp = Wild Type<br>225 bp = Transgene
		R	CTG AAC TTG TGG CCG TTT AC	
		F	AAG GGA GCT GCA GTG GAG TA	
		R	CAG GAC AAC GCC CAC ACA	
Ai65F	[83]	F	AAG GGA GCT GCA GTG GAG TA	300 bp = Wild Type<br>195 bp = Transgene
		R	CCG AAA ATC TGT GGG AAG TC	
		F	CTG TTC CTG TAC GGC ATG G	
		R	GGC ATT AAA GCA GCG TAT CC	
