# Supplementary figures and images for: Brain Endothelial Gap Junction Coupling Enables Rapid Vasodilation Propagation During Neurovascular Coupling
Source: Cell. Author manuscript; Available in PMC 2025 Aug 11. (PMC12337775; doi:10.1016/j.cell.2025.06.030)

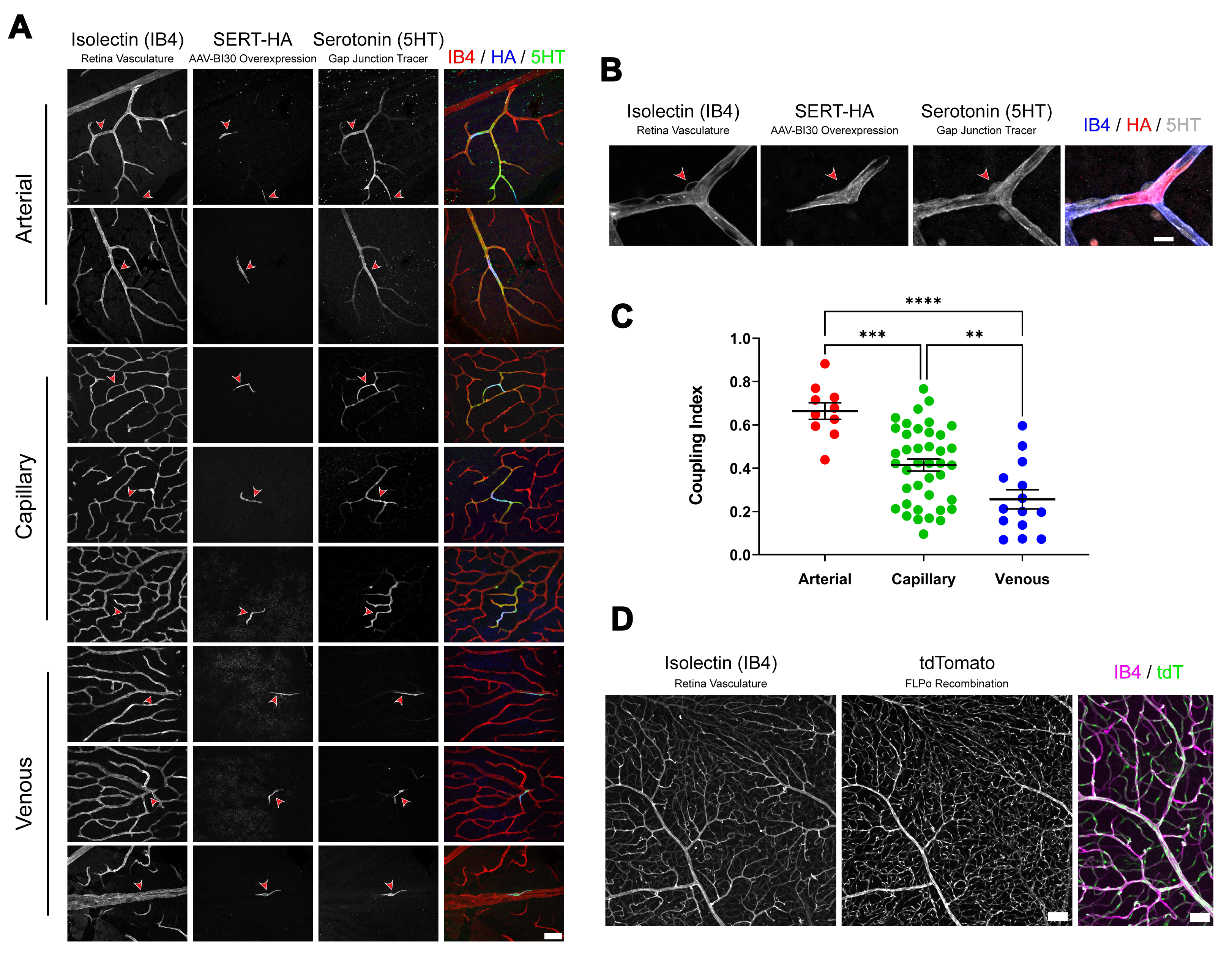

Supplement: 7 — Figure S1. Additional characterization of CNS endothelial gap junction coupling via non-invasive SERT-based tracing. (Related to Figure 1). (A) A cocktail of AAV-BI30 capsids packaged with FLPo or FLP-dependent SERT was intravenously administered to adult mice to achieve sparse-but-strong expression of the transporter in the retinal vasculature. After allowing >6 weeks for overexpression, animals were perfused with a serotonin-containing solution to achieve cell loading. The positions of SERT-HA expressing probe cells are demarcated with red arrowheads. Note attenuation of gap junction coupling strength with progression through the arterio-venous axis. Images are representative of results acquired from n = 5 animals. (B) High-magnification image of serotonin spread from a SERT-HA+ arterial endothelial cell in retina. Note conspicuous labeling of abluminal-facing mural cell (red arrowhead), indicative of gap junction coupling between endothelial and mural layers of the vessel wall. (C) For quantification of variation in cell-cell coupling strength along the arterio-venous axis, SERT+ probe endothelial cells from n = 4 retina whole mounts isolated from n = 3 mice were exhaustively examined. The coupling index presented was defined as the background-corrected intensity ratio of serotonin signal within a given probe cell / serotonin signal within the most proximal 𝑎 × 3 pixels in a mask of contiguous vasculature, where 𝑎 was set as area of the probe cell. A total of n = 10 arterial, 40 capillary, and 14 venous endothelial cells were included in the analysis. Mean ± s.e.m.; one-way ANOVA with Tukey’s multiple comparisons test (** p < 0.01, *** p < 0.001, **** p < 0.0001). Adjusted p values are as follows: arterial vs. capillary, 0.0002; arterial vs. venous, < 0.0001; capillary vs. venous, 0.0092. (D) AAV-BI30:CAG-FLPo-miR122-WPRE was intravenously administered to adult Ai65F FLP-dependent reporter mice (Rosa26:CAG-FRT-Stop-FRT-tdTomato) and recombination was assessed aft [file NIHMS2097217-supplement-7.tif]

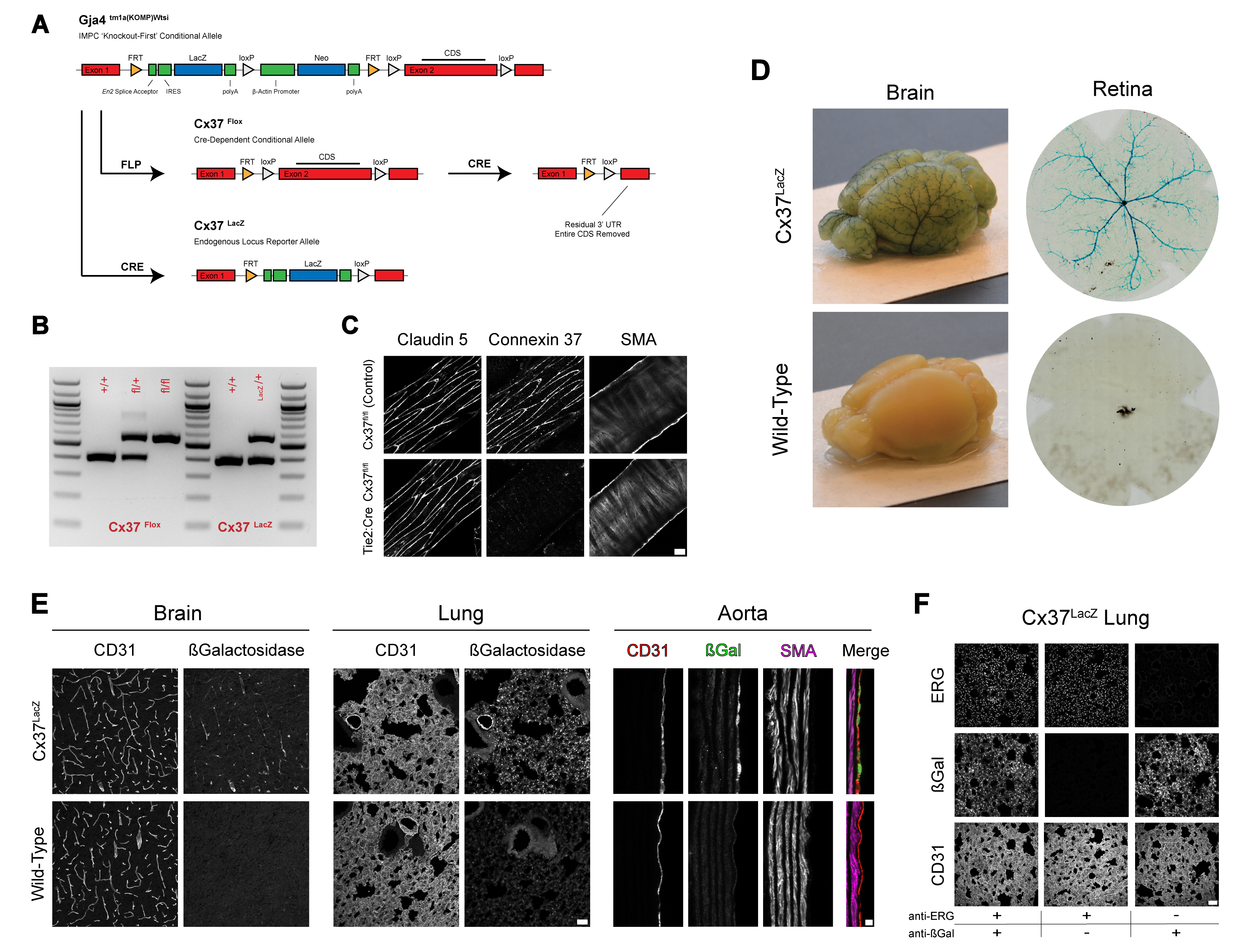

Supplement: 8 — Figure S2. Generation and validation of Cx37 conditional knockout and reporter alleles. (Related to Figure 2). (A) Schematic of International Mouse Phenotyping Consortium (IMPC) ‘knockout-first’ Gja4 conditional allele used to generate Cx37Flox and Cx37LacZ mouse lines. Mating with a germline FLP driver removed the LacZ reporter and Neo selection cassette, producing the conditional Cx37flox allele. Cre-dependent recombination of the Cx37flox allele removes the entire Gja4 coding sequence. The 3’ loxP site is inserted within the 3’ UTR. Importantly, this does not affect the Gja4 coding sequence. Mating with a germline Cre driver removed the Neo selection element and the entire Gja4 coding sequence, producing the Cx37LacZ allele. Because the LacZ reporter’s expression is controlled by the endogenous promoter and enhancer elements present within the Gja4 locus, X-Gal staining and ß-galactosidase immunodetection provide high-fidelity readouts of Cx37 expression. (B) PCR genotyping of Cx37Flox and Cx37LacZ alleles. Primer sequences are described in Supplementary Table 2. (C) Single confocal z-planes of pial arteries in Cx37fl/fl and Tie2:Cre Cx37fl/fl mice demonstrate that Cx37 protein expression from the Cx37Flox allele is overtly indistinguishable from the Cx37WT allele and as expected, the Cx37Flox allele is susceptible to efficient Cre-mediated recombination. (D, E) Comparison of identically treated samples obtained from Cx37LacZ reporter and wild-type mice following enzymatic (D) or immunohistochemical (E) detection. Enzymatic data from Cx37LacZ brain and retina same as shown in Figure 2D and G, respectively. Immunohistochemical detection of ß-Gal in Cx37LacZ aorta and lung same as shown in Figure 3B and S3H–I, respectively. (F) Control immunostaining conditions indicate that the co-localization of ERG and ß-Gal signals observed in lung capillaries is not an artifact of cross-reactivity between their respective primary antibodies, which were both raised in rabbit ho [file NIHMS2097217-supplement-8.tif]

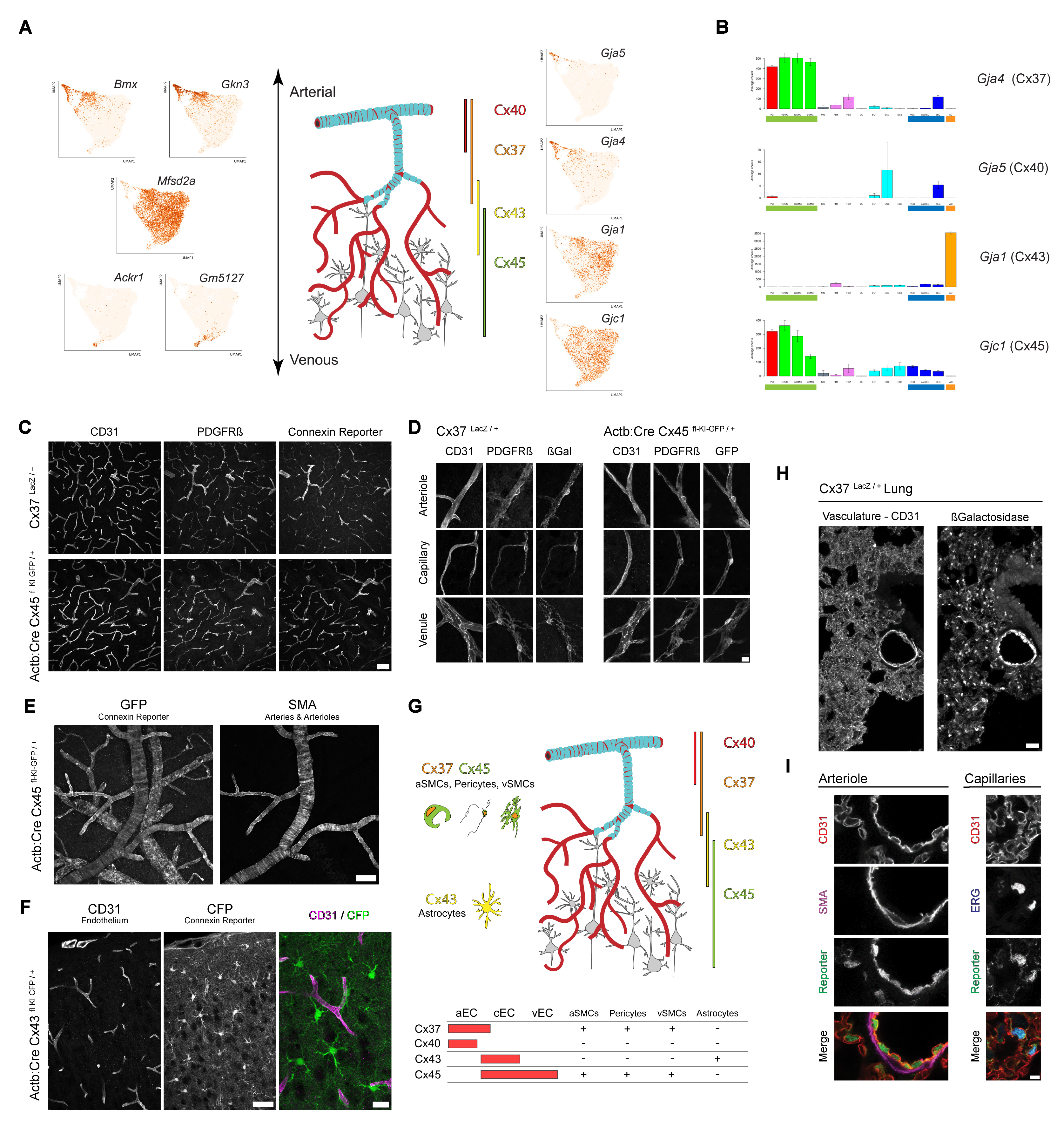

Supplement: 9 — Figure S3. Expression of connexin isoforms across the cerebrovasculature. (Related to Figure 2). (A, B) Published single-cell transcriptomics of the cerebrovasculature corroborate patterns of connexin expression observed with reporter lines. (A) The dataset acquired by Jeong et al. 27 (https://single-cell.mpi-muenster.mpg.de, UMAP seed #42) was used to analyze connexin expression in endothelial cells. Left: to orient the dataset along the arterio-venous axis, arterial-(Bmx 68 and Gkn3 23), capillary- (Mfsd2a 19,23), and venous-specific (Ackr1 69 and Gm5127 70) markers are shown. Right: expression of connexin isoforms Cx40 (Gja5), Cx37 (Gja4), Cx43 (Gja1), and Cx45 (Gjc1) reveals a stepwise pattern of arterio-venous zonation. (B) Data reported by Vanlandewijck et al. 23 (https://betsholtzlab.org/VascularSingleCells/database.html) shows similar patterns of connexin expression in endothelial cells (blue underline) and further reveals expression of these isoforms in other cell types of the neurovascular unit: mural cells (pericytes, aSMCs, and vSMCs; green underline) and astrocytes (orange underline). Cx43 (Gja1) was robustly expressed in astrocytes and both Cx37 (Gja4) and Cx45 (Gjc1) were expressed in mural cells. None of the other 16 connexin isoforms expressed in the mouse genome (Gja3, Gja6, Gja8, Gja10, Gjb1, Gjb2, Gjb3, Gjb4, Gjb5, Gjb6, Gjc2, Gjc3, Gjd2, Gjd3, Gjd4, Gje1) were detectable in endothelial or mural cells at levels above noise in either dataset. Abbreviations shown in (B) are as follows: PC – pericytes (red); SMC – smooth muscle cells (green); MG – microglia (grey); FB – vascular fibroblast-like cells (pink); OL – oligodendrocytes (tan); EC – endothelial cells (blue); ac – Astrocytes (orange); v – venous; capil – capillary; a – arterial; aa – arteriolar. (C, D) Low- (C) and high- (D) magnification images of constitutive Cx37 (Gja4LacZ/+) and Cx45 (Actb:Cre Gjc1fl-KI-GFP/+) reporter expression in the brain microvasculature. Strong expression of both i [file NIHMS2097217-supplement-9.tif]

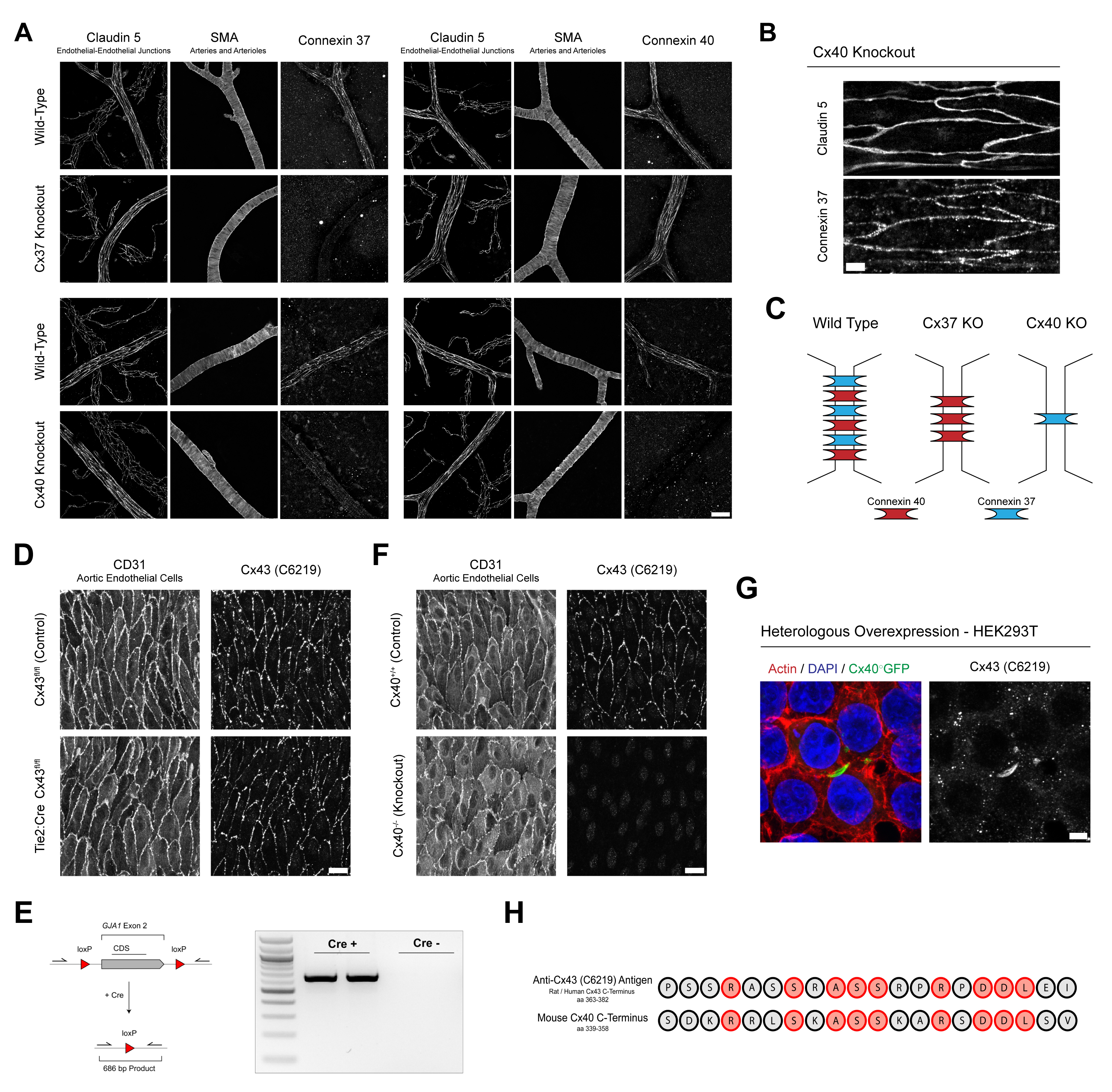

Supplement: 10 — Figure S4. Cx37 and Cx40 are robustly expressed at arterial endothelial cell junctions, while reports of Cx43 expression in arterial endothelium likely stem from antibody cross- reactivity. (Related to Figure 3). (A) Whole-mount immunostaining of the pia vasculature reveals robust expression of Cx37 and Cx40 at arterial (SMA+) but not venous (SMA−) endothelial-endothelial junctions. The signal associated with each connexin isoform was abolished in its respective knockout line, verifying specificity of the antibody reagents. Cx37 expression was strikingly diminished in Cx40 knockout animals. (B) A reduced Cx37 signal was still apparent in individual z-planes of Cx40 knockout arteries acquired at higher laser power. Three observations – (i) complete loss of Cx37 signal in Cx37 knockout animals, (ii) complete loss of Cx40 signal in Cx40 knockout animals, and (iii) persistence of Cx40 signal in Cx37 knockout animals – collectively indicate that Cx37 downregulation is not an artifact of antibody cross-reactivity between the related isoforms. (C) Schematic depicting coordinated regulation of arterial connexins. Cx37 appears to require the presence of Cx40 for its full expression in cerebrovascular arteries, a result consistent with prior reports in the peripheral vasculature 29,45,73–75. Previous studies report that arterial endothelial cells in the aorta 76, periphery 43, and brain 77 robustly express Cx43. These results contrast zonation we observe using an endothelial-specific Cx43 reporter (Tie2:Cre Gja1 flox-KI-CFP) where expression is specifically excluded from large arterial segments of the vasculature. This discrepancy is most likely explained by cross-reactivity of the antisera used in the aforementioned studies, as demonstrated by the following data. (D) En face preparation of aortic endothelium isolated from adult Tie2:Cre Cx43fl/fl mice and Cre- negative controls. Immunostaining with Sigma Anti-Cx43 (C6219) produces an indistinguishable signal at endothelial-e [file NIHMS2097217-supplement-10.tif]

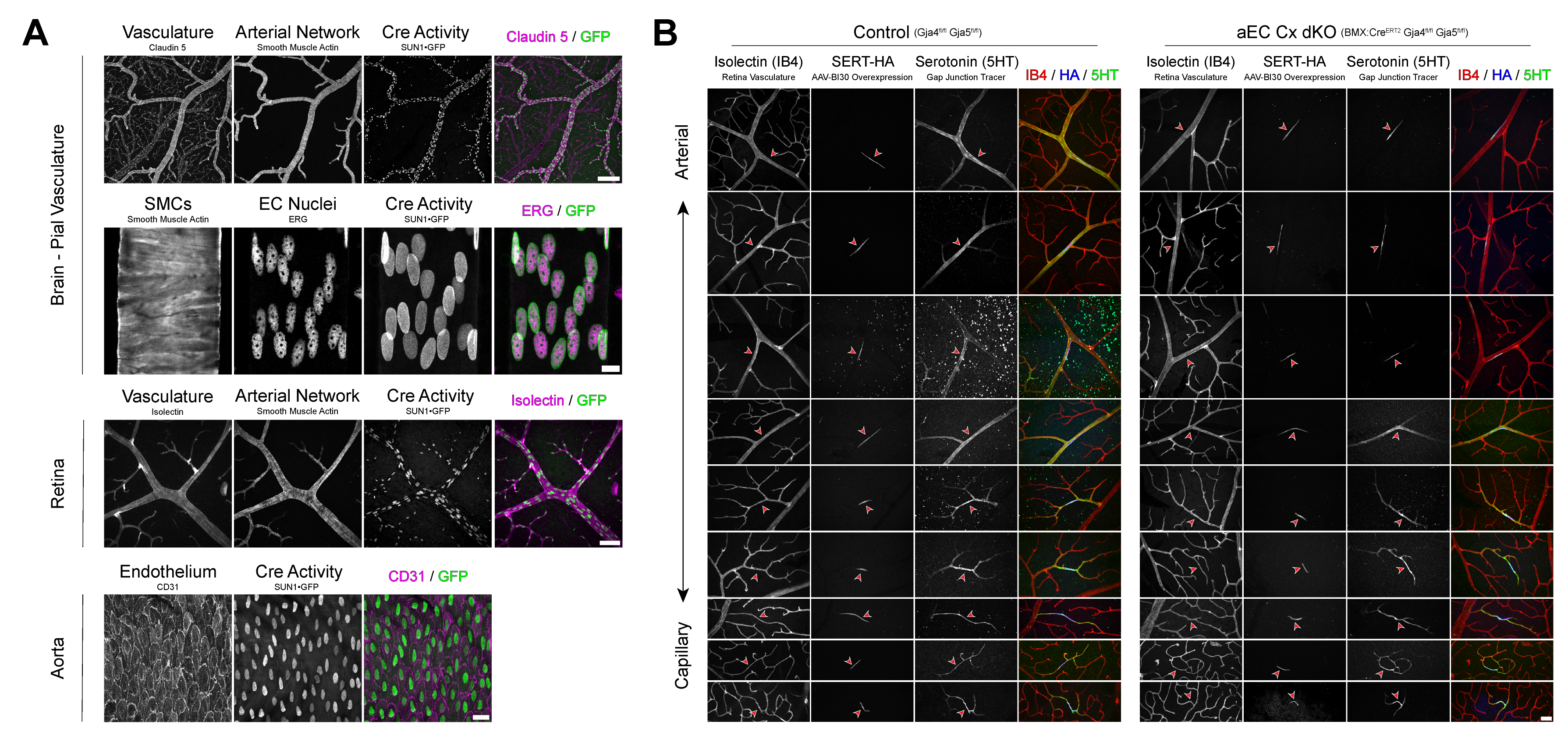

Supplement: 11 — Figure S5. Non-invasive gap junction tracing reveals loss of arterial endothelial-endothelial gap junction coupling in the CNS vasculature following inducible deletion of Cx37 and Cx40. (Related to Figure 3). (A) Validation of the BMX:CreERT2 driver used for conditional loss-of-function studies. Adult BMX:CreERT2 SUN1◦GFP reporter animals (Rosa26:CAG-LSL-Sun1◦GFP) received 1mg / day tamoxifen for five consecutive days. Tissues were harvested two weeks after the final dose to assess recombinase activity, indicated by overexpression of an inner nuclear membrane-localized GFP. Efficient, predominantly arterial endothelial cell-specific recombination was observed in brain, retina, and aorta. Images are representative of n = 3 animals. (B) A cocktail of AAV-BI30 capsids packaged with FLPo or FLP-dependent SERT was intravenously administered to adult BMX:CreERT2 Gja4fl/fl Gja5fl/fl and Gja4fl/fl Gja5fl/fl controls. Animals were subsequently treated with tamoxifen, and non-invasive gap junction tracing was performed ~6 – 9 weeks following the final dose in n = 4 animals per genotype. The positions of SERT-HA expressing probe cells in the retinal vasculature are demarcated with red arrowheads. Uppermost examples for each genotype same as shown in Figure 3G. Note robust intercellular serotonin diffusion in the large arteries of control animals. By contrast, serotonin accumulation is restricted to individual arterial endothelial cells in connexin-knockout animals, indicative of a near-complete loss of gap junction coupling. Gap junction coupling reappears in the small arterioles and capillaries of these animals, such that the two genotypes phenotypically converge with progression along the arterio-venous axis. Compelling tracer-spreading events in the large arteries of control animals were rare and observed in n = 2/4 mice examined; strong cell-cell coupling at these sites required both high levels of SERT overexpression and efficient cell-loading during intracardiac perfusio [file NIHMS2097217-supplement-11.tif]

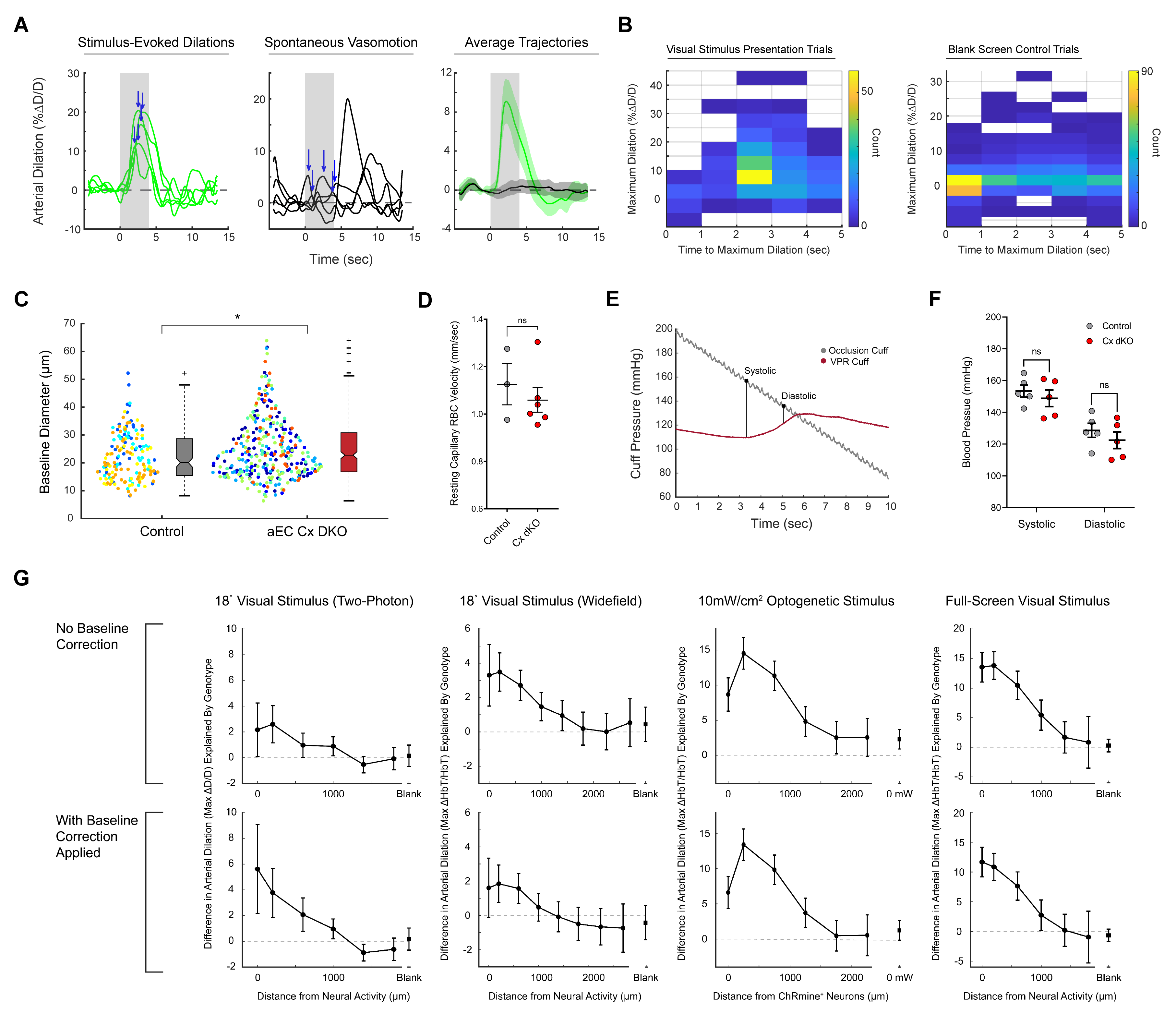

Supplement: 12 — Figure S6. Additional data and analyses relevant to long-range vasodilation propagation deficits observed in endothelial gap junction coupling mutants. (Related to Figure 4). (A) Left: Example single-trial vasodilation responses evoked by presentation of 18° visual stimuli at sites proximal to activity (<1µm). Blue arrows indicate maximum dilation calculated from each trajectory. Duration of visual stimulation is demarcated in gray. Center: Example responses captured during blank screen control trials. Spontaneous vasomotion results in non-zero detected maximum dilations. Note that identification of maximum response is restricted to the 4-second window normally associated with stimulus presentation. Right: Averaging trajectories smooths out spontaneous vasomotion while preserving stimulus-evoked vasodilation (hierarchical bootstrap; mean ± 95% CI) because the former is not correlated with trial structure. (B) Two-dimensional histograms of calculated maximum dilation versus time show clear time- locking to stimulus in visual-presentation but not blank screen trials. Data shown was acquired from tamoxifen-treated Gja4fl/fl Gja5fl/fl control animals via two-photon imaging (n = 9 mice, 344 trajectories for stimulus-presentation trials; n = 6 mice, 705 trajectories for blank screen control trials). (C) Resting pial arterial diameter of tamoxifen-treated BMX:CreERT2 Gja4fl/fl Gja5fl/fl and Gja4fl/fl Gja5fl/fl controls measured in vivo via two-photon microscopy. Swarmplots show all measurements; each circle represents an artery, each color an individual mouse (n = 6 mutant / 4 control mice). Boxplots depict the same data, treating all vessels as independent observations. Midline is plotted at the median, with boxes extending to the 25th and 75th percentiles of the data. Outliers are plotted as ‘+’ symbols. Comparison with a Wilcoxon rank-sum test indicated a significant difference between the populations (p = 0.0235). (D) Resting capillary red blood cell (RBC) velocity was [file NIHMS2097217-supplement-12.tif]

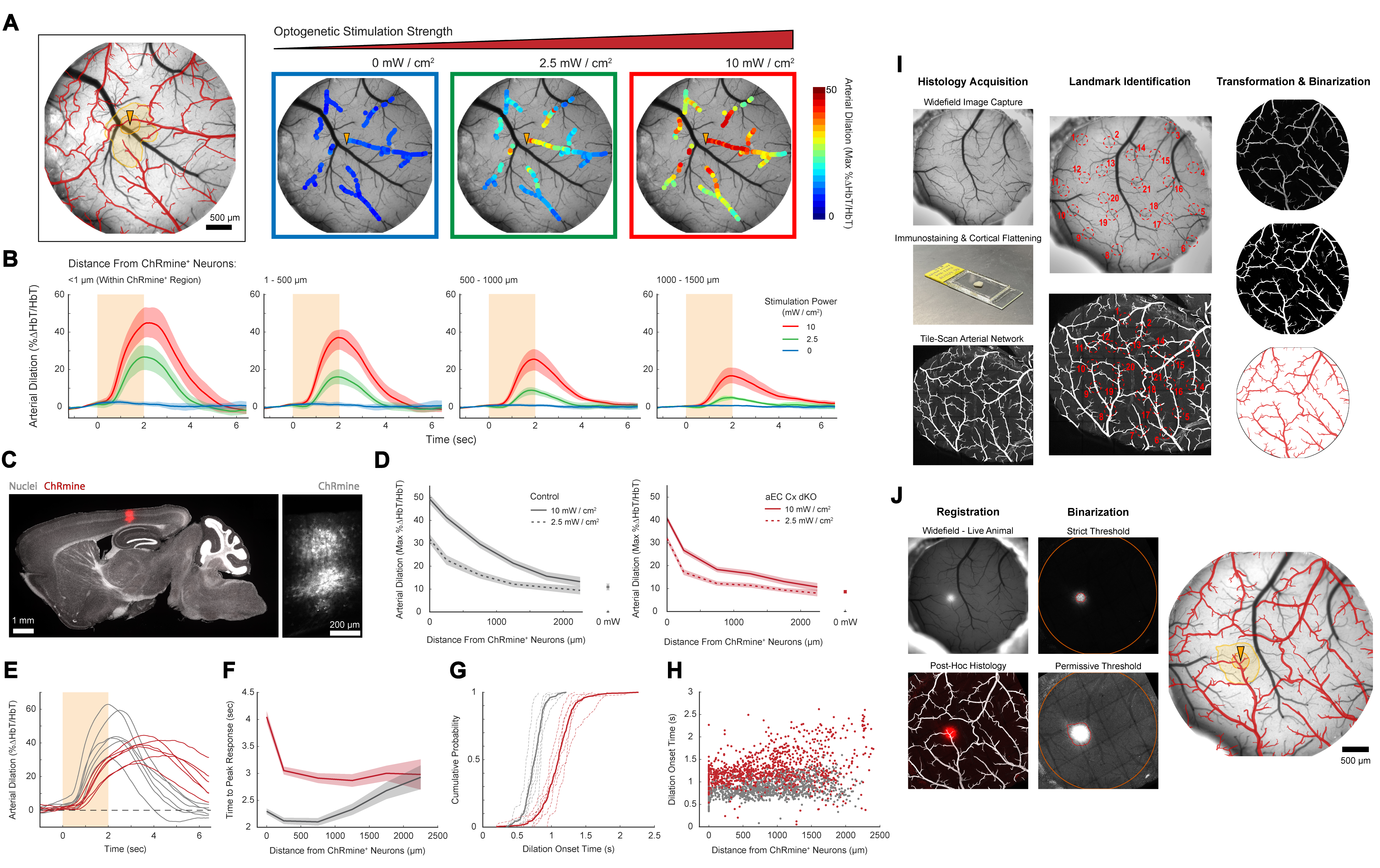

Supplement: 13 — Figure S7. Optogenetic stimulation enables precise and scalable control of neurovascular coupling, revealing aberrant vasodilation kinetics in endothelial gap junction-deficient mutants. (Related to Figure 5). (A) Representative example of scalable responses in a control mouse (tamoxifen-treated Gja4fl/fl Gja5fl/fl). Leftmost image shows arterial network in red and extent of ChRmine-expressing neurons in orange. Arrowhead indicates center of transduction. Maximum dilation responses at each location during the imaging window are shown for 0, 2.5, and 10 mW/cm2 conditions. Note long- range recruitment of arterial responses at highest stimulation power (also see Supplementary Movie 2). (B) Average optogenetically-evoked dilation trajectories at the same three stimulation intensities, controlling for distance from ChRmine expression (n = 6 tamoxifen-treated Gja4fl/fl Gja5fl/f controls; 10mW/cm cm2 responses same as shown in Figure 6C). (C) Left: representative sagittal brain section, demonstrating focal ChRmine overexpression in somatosensory cortex following stereotaxic injection. Right: a column of transduced neurons in the opsin-expressing region. (D) Aggregated dilation responses of tamoxifen-treated BMX:CreERT2 Gja4fl/fl Gja5fl/fl mice and Gja4fl/fl Gja5fl/f controls at 0, 2.5, and 10mW/cm2 stimulation intensities (linear mixed-effect model; mean ± 95% CI; n = 6 animals of each genotype; 0 and 10mW/cm2 responses same as shown in Figure 5B,C). Control (0 mW/cm2) responses are pooled into a single datapoint, irrespective of position in arterial network. (E)Average arterial dilation trajectories obtained from individual mice at locations within <1µm of ChRmine expression (by-genotype average trajectories are shown in Figure 5C). Data from each mouse is plotted as a separate trajectory. Note highly stereotyped differences in trajectory shape across genotypes. (F)Comparison of time-to-peak dilation across genotypes, controlling for distance from ChRmine expression (line [file NIHMS2097217-supplement-13.tif]
